# Supplementary material for: Synthesis of precisely functionalizable curved nanographenes via graphitization-induced regioselective chlorination in a mechanochemical Scholl Reaction
Source: Nat Commun. 2023 Feb 13;14:803. doi: 10.1038/s41467-023-36470-8 (PMC9925806; doi:10.1038/s41467-023-36470-8)
Supplement: Supplementary file 1 — Supplementary Information [file 41467_2023_36470_MOESM1_ESM.pdf]

Supplementary Information

Synthesis of Precisely Functionalizable Curved Nanographenes via Graphitization-Induced Regioselective Chlorination in a Mechanochemical Scholl Reaction

Jovana Stanojkovic, Ronny William, Zhongbo Zhang, Israel Fernández, Jingsong Zhou, Richard D. Webster, and Mihaiela C. Stuparu*
*Email:mstuparu@ntu.edu.sg

Supplementary Methods

All solution phase reactions were carried out in oven-dried glassware under argon atmosphere unless stated otherwise. All mechanochemical reactions were prepared in an argon-filled glove box using ZrO_2 (15 mL, Form-Tech Scientific) grinder jars along with ZrO_2 miller balls. The milling process was performed on Retsch Mixer Mill MM400 machine. All microwave-assisted reactions were carried out with Anton Paar Monowave 400 microwave reactor. Anhydrous 1,2-dichloroethane, acetonitrile, and DMI were purchased from commercial suppliers and used without prior distillation. Anhydrous dichloromethane was distilled over $CaCl_2$ and 1,4-dioxane over sodium under nitrogen atmosphere. Thin layer chromatography was performed on silica-gel coated glass plates (60 F_{254}, Merck). Visualization of TLC was achieved by means of irradiation with *uv* light at 254 nm and/or 365 nm. Product purification by flash column chromatography was accomplished using Davisil® silica gel 60 (40-63μm) or Florisil® (100-200 mesh) with technical grade solvents that were distilled prior to use. Preparative layer chromatography (PLC) was carried out on silica-gel coated glass plates (60 F_{254}, Merck) with a thickness of 0.5 mm.

High resolution masses were obtained by MALDI-TOF using ABI 4800. ^{1}H and ^{13}C NMR spectra recorded at room temperature was performed on Bruker DPX 400, and Bruker AMX 500 nuclear magnetic spectrometers. ^{13}C NMR spectra at elevated temperature was obtained using JEOL ECA400. NMR spectra were recorded at room temperature or low temperature on a 400 MHz and 500 MHz (and the corresponding frequencies for ^{13}C) Bruker and Jeol ECA NMR spectrometers. Chemical shifts for ^{1}H NMR spectra are reported as δ in units of parts per million (ppm) relative to residual solvent peak of chloroform-d1 (δ 7.26, singlet) or tetrachloroethane-d2 (δ 6.00,

singlet) and coupling constant (*J*) are reported in Hz. Multiplicities are reported as follows: s (singlet); brs (broad singlet); d (doublet); t (triplet); q (quartet); dd (doublet of doublet); ddd (doublet of doublet of doublet); dt (doublet of triplet); m (multiplet) and etc. Carbon nuclear magnetic resonance spectra (^{13}C NMR) are reported as δ in units of parts per million as referenced to residual solvent peaks (chloroform-d1: δ 77.23, triplet; tetrachloroethane-d2: δ 73.78, triplet). X-ray crystallographic data was collected by using a Bruker X8Apex diffractometer with Mo K/α radiation (graphite monochromator). UV-Vis absorption spectra were recorded on Ailgent Cary 8454 diode-array spectrophotometer and Cary 300 UV/vis spectrophotometer in CH_2Cl_2 at room temperature. Fluorescence emission data were recorded on a Cary Eclipse spectrophotometer in CH_2Cl_2 at room temperature.

Diffraction intensity data were measured either at 103 K with a Bruker Kappa diffractometer equipped with a CCD detector, employing either Mo Kα (λ = 0.71073 Å) radiation, with the SMART suite of programs (SMART version 5.628; Bruker AXS Inc., Madison, WI, USA, 2001). Structural solution and refinement were carried out with the SHELXTL suite of programs (Sheldrick, G. M. University of Göttingen: Göttingen, Germany, 2014). The intensities were corrected for Lorentz and polarization effects. The non-hydrogen atoms were refined anisotropically. Hydrogen atoms were placed using AFIX instructions. The crystal structure of **7** was solved by Squeeze/Platon (A. L. Spek, Acta Cryst. (2015). C71, 9-18) and refined as a two-component non-merohedral twin with BASF parameter and HKLF 5 reflection file obtained by TwinRotMat routine of PLATON (A. L. Spek, Acta Cryst. (1990). A46, c34).

Voltammetric measurements were performed using a Metrohm Autolab PGSTAT302N potentiostat in a three-electrode setup. A 1 mm diameter planar glassy carbon (GC) disk (eDAQ) was used as a working electrode in conjunction with a platinum wire counter electrode (Metrohm) and a silver wire miniature reference electrode (eDAQ) connected to the test solution via a salt bridge containing 0.5 M tetra-*n*-butylammonium hexafluorophosphate (*n*-Bu_4NPF_6) in CH_2Cl_2. Tetra-*n*-butylammonium hexafluorophosphate was prepared[1] by reacting equal molar amounts of aqueous solutions of tetrabutylammonium hydroxide (40%) with hexafluorophosphoric acid (65%) (Sigma-Aldrich). The resulting white precipitate was washed with hot ultrapure water until the filtrate tested as neutral pH. The *n*-Bu_4NPF_6 was recrystallized three times from hot analytical grade ethanol and then

dried under vacuum for at 140 °C for 12 hours using a Buchi B-585 glass oven before use as the supporting electrolyte. Cyclic voltammetry (CV) was performed at a scan rate of 0.1 V s^{-1}, while square-wave voltammetry (SWV) was recorded with a pulse period (τ) of 25 Hz, a potential step of 2 mV, and a pulse amplitude of 20 mV. All voltammetric experiments were conducted under an argon atmosphere, at room temperature in a Faraday cage. Prior to each scan, the working electrode was cleaned by polishing with alumina oxide (grain size 0.3 μm) slurry on a Buehler Ultra-pad polishing cloth, rinsing with acetone, and then dried with a lint free tissue. In accordance with IUPAC recommendations, the absolute potentials were calibrated using ferrocene (Fc) as an internal reference, which was added to the test solution at the end of the measurements.

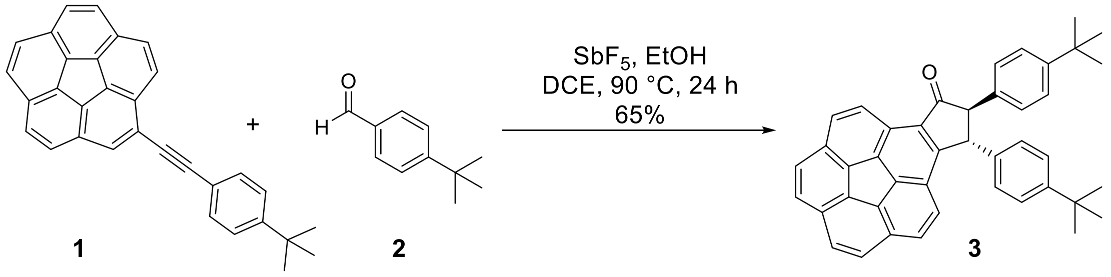

To a solution of 1-[(4-*tert*-butylphenyl)ethynyl]corannulene **1** (485 mg, 1.2 mmol, 1equiv) and 4-*tert*-butylbenzaldehyde **2** (239 μL, 1.4 mmol, 1.2 equiv) in anhydrous 1,2-dichloroethane (12 mL) was added a solution of SbF_5 (8.6 μL, 0.12 mmol, 0.1 equiv) in EtOH (69.5 μL, 1.2 mmol, 1 equiv) at room temperature. The reaction mixture was stirred at 90 °C for 24 hours before being quenched by addition of saturated aqueous $NaHCO_3$ solution (3 mL). The resulting mixture was extracted with CH_2Cl_2 (3 × 15 mL). The combined organic layers were washed with brine (5 mL), dried over Na_2SO_4, and evaporated under reduced pressure. The crude residue was purified by flash column chromatography on silica gel (hexane/CH_2Cl_2= 3:1) to afford **3** (444.6 mg, 65%) as yellow solid: HRMS (MALDI-TOF): m/z $[M+H]^+$ calcd for $C_{43}H_{37}O$ 569.2839, found 569.2846. ^1H NMR (400 MHz, $CDCl_3$) δ 8.63 (d, *J* = 8.8 Hz, 1H), 7.93 (d, *J* = 8.8 Hz, 1H), 7.87 – 7.79 (m, 3H), 7.76 (d, *J* = 8.8 Hz, 1H), 7.69 (d, *J* = 8.8 Hz, 1H), 7.50 (d, *J* = 8.8 Hz, 1H), 7.38 (d, *J* = 8.1 Hz, 2H), 7.29 (dd, *J* = 8.1, 5.4 Hz, 4H), 7.03 (d, *J* = 8.1 Hz, 2H), 4.98 (d, *J* = 3.3 Hz, 1H), 4.02 (d, *J* = 3.3 Hz, 1H), 1.34 (s, 9H), 1.28 (s, 9H). ^{13}C NMR (101 MHz, $CDCl_3$) δ 206.42, 162.82, 150.28, 150.00, 140.48, 139.95, 136.71, 136.69, 135.96, 135.48, 135.15, 134.17, 132.22, 131.44, 130.92, 129.01, 128.84, 127.85, 127.75, 127.71, 127.64, 127.61,

127.56, 127.29, 126.98, 126.20, 126.14, 125.95, 125.59, 64.47, 54.12, 34.69, 34.59, 31.53, 31.46.

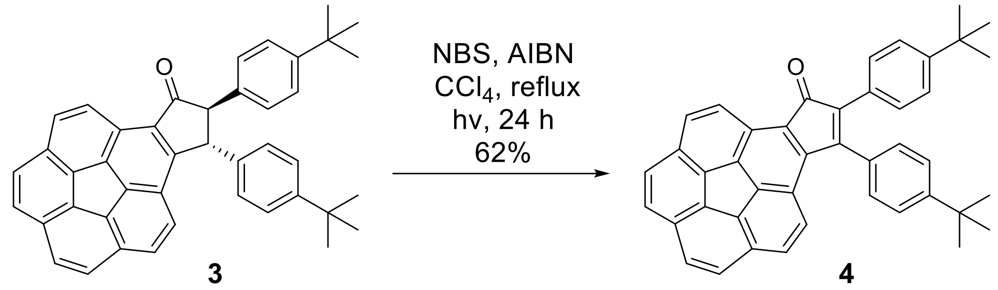

To a solution of **3** (56.8 mg, 0.1 mmol, 1 equiv) in CCl_4 (1 mL) was added *N*-bromosuccinimide (19.6 mg, 0.11 mmol, 1.1 equiv) and azobisisobutyronitrile (0.17 mg, 0.001 mmol, 0.01 equiv) at room temperature. The reaction mixture was then stirred at reflux overnight with irradiation using 500W tungsten lamp as light source. Upon completion of the reaction, the reaction mixture was cooled down to room temperature and subsequently filtered through a short pad of celite, washed with CH_2Cl_2 (3 × 5 mL) and evaporated under reduced pressure. The crude residue was purified by flash column chromatography on silica gel (hexane/CH_2Cl_2= 2:1) to afford **4** (35.1 mg, 62%) as purple solid: HRMS (MALDI-TOF): m/z $[M]^+$ calcd for $C_{43}H_{34}O$ 566.2604, found 566.2578. ^1H NMR (400 MHz, $CDCl_3$) δ 8.32 (d, *J* = 8.9 Hz, 1H), 7.77 (d, *J* = 2.4 Hz, 1H), 7.75 (d, *J* = 2.2 Hz, 1H), 7.72 (d, *J* = 3.0 Hz, 2H), 7.63 (d, *J* = 8.7 Hz, 1H), 7.59 - 7.53 (m, 4H), 7.45 (d, *J* = 9.0 Hz, 1H), 7.33 - 7.26 (m, 4H), 6.76 (d, *J* = 9.0 Hz, 1H), 1.46 (s, 9H), 1.31 (s, 9H). ^{13}C NMR (101 MHz, $CDCl_3$) δ 199.22, 154.39, 152.76, 150.64, 149.96, 138.99, 136.44, 135.52, 135.46, 135.39, 132.08, 131.35, 130.96, 130.46, 130.09, 129.81, 129.26, 128.61, 127.87, 127.45, 127.40, 127.23, 126.97, 126.52, 126.31, 125.91, 125.86, 125.64, 125.11, 124.68, 35.13, 34.76, 31.57, 31.40.

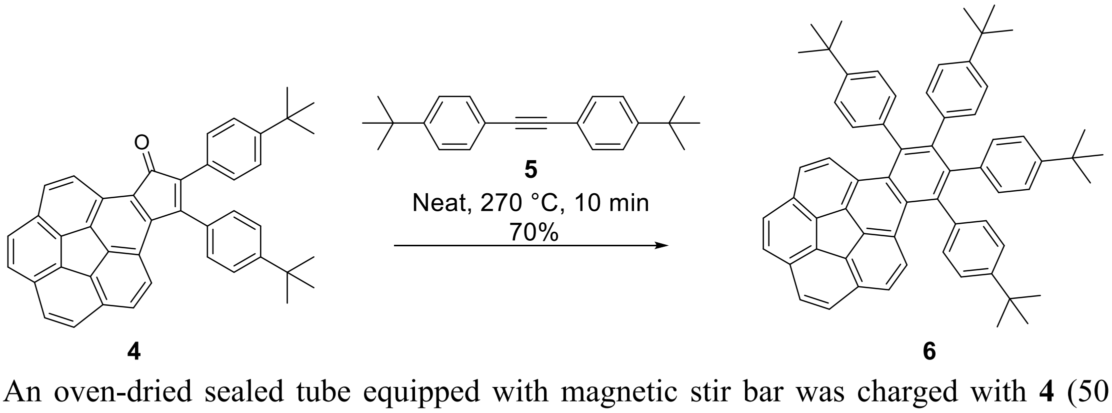

An oven-dried sealed tube equipped with magnetic stir bar was charged with **4** (50

mg, 0.09 mmol, 1 equiv) and bis(4-*tert*-butylphenyl)ethyne **5** (51.5 mg, 0.18 mmol, 2 equiv). The mixture of solids was then heated at 270 °C for 10 minutes. After cooling down to room temperature, the crude residue was subjected to flash column chromatography on silica gel (hexane/CH_2Cl_2= 4:1) to afford **6** (53.5 mg, 70%) as yellowish white solid: HRMS (MALDI-TOF): m/z $[M]^+$ calcd for $C_{64}H_{60}$ 828.4690, found 828.4660. ^{1}H NMR (400 MHz, $CDCl_3$) δ 7.75 (d, J = 8.8 Hz, 2H), 7.69 (d, J = 8.7 Hz, 2H), 7.38 – 7.27 (m, 10H), 6.86 (d, J = 8.4 Hz, 4H), 6.79 (d, J = 8.2 Hz, 4H), 6.70 (d, J = 9.0 Hz, 2H), 1.33 (s, 18H), 1.10 (s, 18H). ^{13}C NMR (101 MHz, $CDCl_3$) δ 149.72, 147.73, 142.06, 140.76, 140.03, 137.65, 135.58, 134.49, 132.65, 131.19, 131.00, 130.35, 129.95, 129.23, 126.98, 126.65, 125.65, 124.93, 123.10, 34.65, 34.21, 31.57, 31.31.

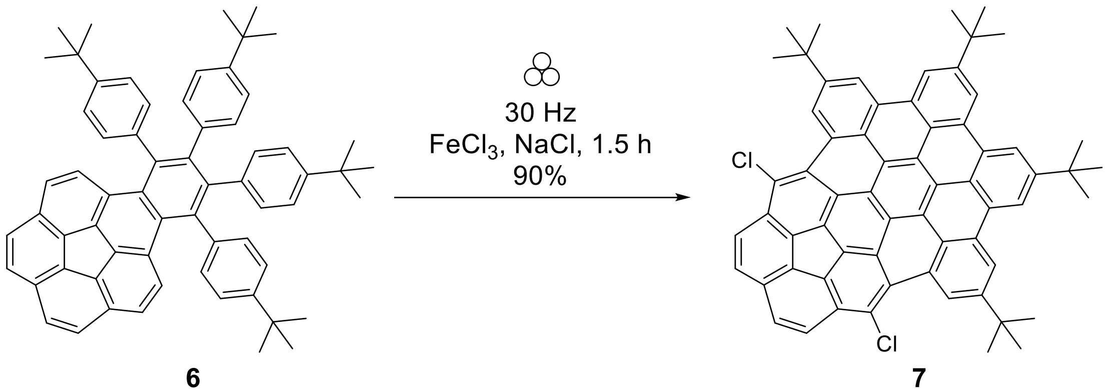

To a 15 mL ZrO_2 jar equipped with 1 piece of 10mm Ø ZrO_2 ball were added **6** (10.3 mg, 0.012 mmol, 1 equiv), anhydrous $FeCl_3$ (145.0 mg, 0.89 mmol, 74.1 equiv), and NaCl (1.444 g). The jar was placed into a glove box, assembled, and sealed with Teflon™ tape and parafilm. The jar was then transferred into the milling machine and milled for (1.5 hours) at a frequency of 30 Hz. The crude mixture was dissolved in CH_2Cl_2 (10 mL) and washed with water. The aqueous layer was then extracted with CH_2Cl_2 (3 × 15 mL). The combined organic layers were washed with brine (5 mL), dried over Na_2SO_4, and evaporated under reduced pressure. The crude residue was purified by passing through a short pad of silica gel column (hexane/CH_2Cl_2= 1:1) to afford **7** (9.9 mg, 90 %) as orange solid: HRMS (MALDI-TOF): m/z $[M]^+$ calcd for $C_{64}H_{48}Cl_2$ 886.3128, found 886.3173. ^{1}H NMR (400 MHz, CDCl3) δ 9.85 (s, 2H), 9.20 (d, J = 14.1 Hz, 6H), 7.92 (d, J = 8.9 Hz, 2H), 7.46 (d, J = 8.8 Hz, 2H), 1.81 (s, 36H). ^{13}C NMR (101 MHz, $CDCl_3$) δ 149.15, 148.29, 131.84, 130.74, 130.55, 130.41, 130.17, 130.14, 129.95, 129.86, 129.64, 128.76, 127.69, 127.25, 127.01, 126.68,

126.57, 126.06, 125.03, 123.15, 120.86, 120.42, 119.73, 119.32, 119.06, 118.83, 35.95, 35.89, 32.22, 32.03.

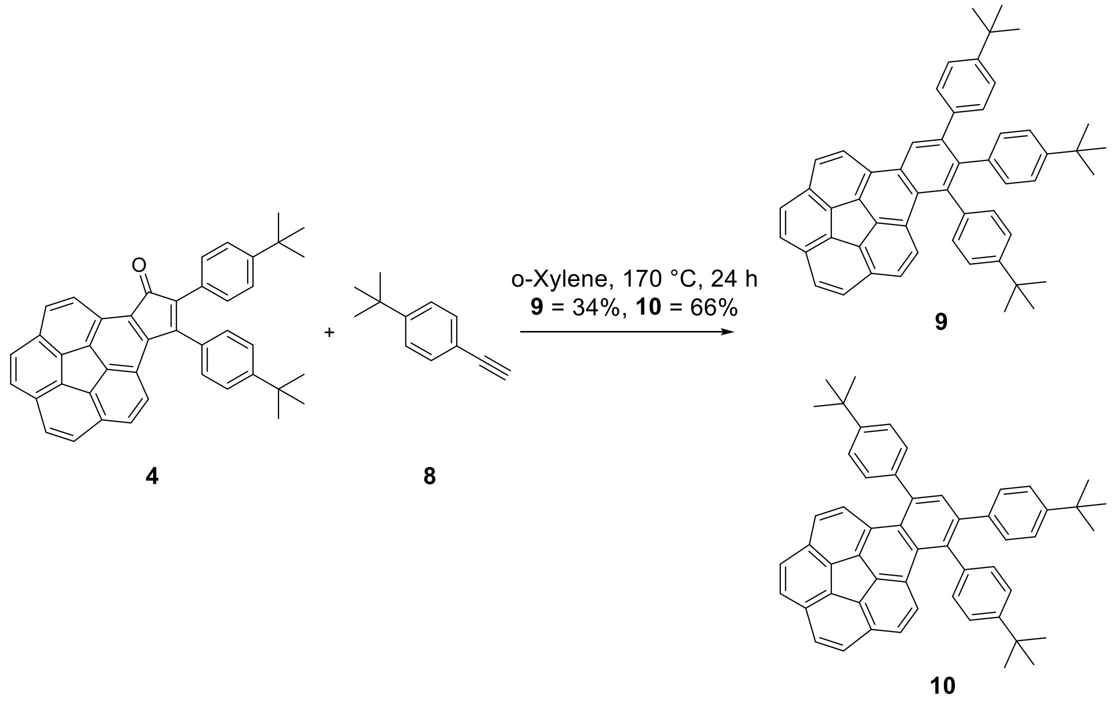

To a sealed tube equipped with magnetic stir bar was added **4** (23.3 mg, 0.04 mmol, 1 equiv) and 4-*tert*-butylphenylacetylene **8** (16.0 µL, 0.08 mmol, 2 equiv). The reaction mixture was heated at 170 °C for 24 hours. Upon completion, the solvent was removed under high vacuum. The resulting crude mixture was purified by flash column chromatography on silica gel (hexane/CH_2Cl_2= 10:1) to afford **9** (10.0 mg, 34%) as white solid and **10** (19.0 mg, 66%) as white solids. **9**: HRMS (MALDI-TOF): m/z $[M]^+$ calcd for $C_{54}H_{48}$ 696.3751, found 696.3801. ^{1}H NMR (400 MHz, $CDCl_3$) δ 8.76 (s, 1H), 8.32 (d, *J* = 8.7 Hz, 1H), 7.96 (d, *J* = 8.6 Hz, 1H), 7.86 (d, *J* = 8.8 Hz, 1H), 7.81 (d, *J* = 8.7 Hz, 1H), 7.77 (d, *J* = 8.7 Hz, 1H), 7.70 (d, *J* = 8.7 Hz, 1H), 7.37 – 7.18 (m, 9H), 6.98 – 6.93 (m, 2H), 6.80 – 6.75 (m, 2H), 6.61 (d, *J* = 8.9 Hz, 1H), 1.35 (s, 9H), 1.30 (s, 9H), 1.18 (s, 9H). ^{13}C NMR (101 MHz, $CDCl_3$) δ 149.98, 149.32, 148.41, 141.76, 140.75, 140.69, 139.45, 139.20, 137.82, 137.02, 135.47, 135.24, 135.05, 135.01, 132.93, 131.98, 131.35, 131.20, 130.64, 130.61, 130.52, 129.94, 129.86, 128.98, 128.73, 127.69, 127.13, 127.04, 126.96, 126.89, 125.91, 125.84, 125.04, 124.54, 124.22, 123.63, 34.69, 34.55, 34.35, 31.60, 31.47, 31.40. **10**: HRMS (MALDI-TOF): m/z $[M]^+$ calcd for $C_{54}H_{48}$ 696.3751, found 696.3759. ^{1}H NMR (400 MHz, $CDCl_3$) δ 7.77 – 7.67 (m, 6H), 7.66 (s, 1H), 7.59 (d, *J* = 8.0 Hz, 2H), 7.41 – 7.28 (m, 6H), 7.17 (d, *J* = 8.1 Hz, 2H), 7.07 (d, *J* = 8.1 Hz, 2H), 6.99 (d, *J*

= 8.9 Hz, 1H), 6.71 (d, J = 9.0 Hz, 1H), 1.47 (s, 9H), 1.40 (s, 9H), 1.27 (s, 9H). ^{13}C NMR (101 MHz, $CDCl_3$) δ 150.74, 150.42, 149.04, 141.56, 141.44, 140.84, 139.52, 139.37, 138.88, 138.11, 136.02, 135.84, 134.80, 134.70, 134.03, 132.72, 132.03, 131.35, 130.70, 130.62, 130.51, 130.10, 129.79, 129.60, 129.43, 129.30, 128.93, 127.05, 127.03, 126.77, 126.71, 125.90, 125.76, 125.59, 125.41, 124.29, 34.89, 34.78, 34.49, 31.70, 31.65, 31.45.

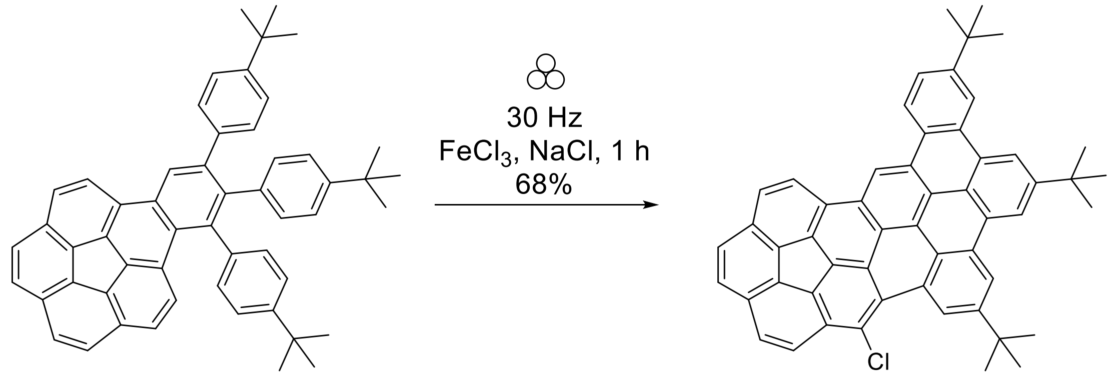

9 **11**

To a 15 mL ZrO_2 jar equipped with 1 piece of 10mm Ø ZrO_2 ball were added **9** (7 mg, 0.01 mmol, 1 equiv), anhydrous $FeCl_3$ (116.7 mg, 0.72 mmol, 72 equiv), and NaCl (1.526 g). The jar was placed into a glove box, assembled, and sealed with Teflon™ tape and parafilm. The jar was then transferred into the milling machine and milled for 1 hour at a frequency of 30 Hz. The crude mixture was dissolved in CH_2Cl_2 (10 mL) and washed with water. The aqueous layer was then extracted with CH_2Cl_2 (3 × 5 mL). The combined organic layers were washed with brine (5 mL), dried over Na_2SO_4, and evaporated under reduced pressure. The crude residue was purified by flash column chromatography on silica gel (hexane/CH_2Cl_2= 4:1) to afford **11** (5.0 mg, 68%) as yellow solid: HRMS (MALDI-TOF): m/z $[M]^+$ calcd for $C_{54}H_{41}Cl$ 724.2891, found 724.2814. ^{1}H NMR (500 MHz, $CDCl_3$) δ 9.95 (d, J = 1.7 Hz, 1H), 9.55 (s, 1H), 9.13 (s, 1H), 9.08 (s, 1H), 8.92 (s, 1H), 8.78 (t, J = 4.4 Hz, 2H), 8.09 (d, J = 8.5 Hz, 1H), 8.04 (d, J = 8.8 Hz, 1H), 7.86 (dd, J = 8.5, 1.8 Hz, 1H), 7.65 (d, J = 8.7 Hz, 2H), 7.61 – 7.54 (m, 2H), 1.81 (s, 9H), 1.71 (s, 9H), 1.64 (s, 9H). ^{13}C NMR (100 MHz, CD_2Cl_2) δ 150.77, 149.42, 148.60, 136.24, 136.20, 135.09, 133.99, 130.90, 130.78, 130.57, 130.51, 130.37, 130.27, 130.24, 130.22, 130.16, 130.13, 129.91, 129.86, 129.84, 129.78, 129.33, 128.45, 127.96, 127.84, 127.56, 127.53, 127.39, 127.15, 126.52, 126.12, 126.08, 125.86, 125.74, 125.49, 125.44, 124.78,

124.58, 123.82, 123.77, 123.17, 122.51, 122.09, 121.82, 119.60, 119.59, 119.57, 119.49, 118.77, 118.40, 118.35, 116.45, 35.53, 35.49, 34.97, 31.76, 31.68, 31.37.

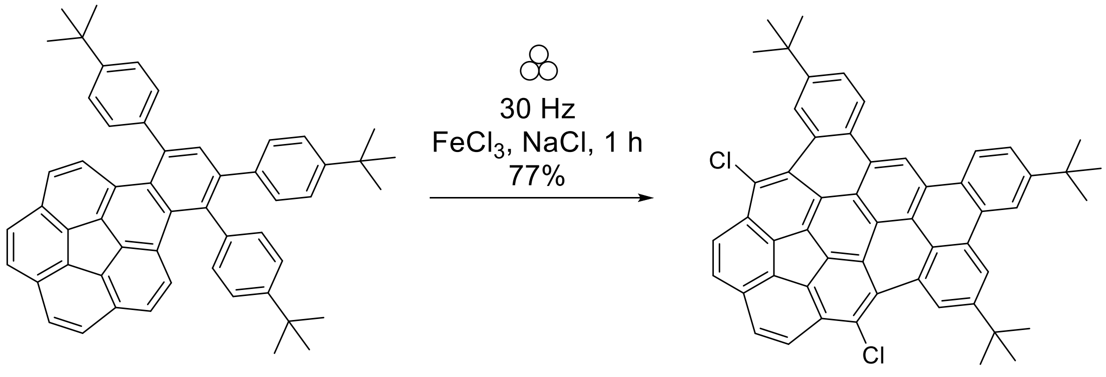

10 **12**

To a 15 mL ZrO_2 jar equipped with 1 piece of 10mm Ø ZrO_2 ball were added **10** (7 mg, 0.01 mmol, 1 equiv), anhydrous $FeCl_3$ (116.7 mg, 0.72 mmol, 72 equiv), and NaCl (1.526 g). The jar was placed into a glove box, assembled, and sealed with Teflon™ tape and parafilm. The jar was then transferred into the milling machine and milled for 1 hour at a frequency of 30 Hz. The crude mixture was dissolved in CH_2Cl_2 (10 mL) and washed with water. The aqueous layer was then extracted with CH_2Cl_2 (3 × 5 mL). The combined organic layers were washed with brine (5 mL), dried over Na_2SO_4, and evaporated under reduced pressure. The crude residue was purified by flash column chromatography on silica gel (hexane/CH_2Cl_2= 4:1) to afford **12** (5.9 mg, 77%) as yellow solid: HRMS (MALDI-TOF): m/z $[M]^+$ calcd for $C_{54}H_{40}Cl_2$ 758.2502, found 758.2550. ^{1}H NMR (400 MHz, $CDCl_3$) δ 9.32 (s, 1H), 9.28 (d, *J* = 2.0 Hz, 1H), 8.86 (s, 1H), 8.60 (d, *J* = 2.0 Hz, 1H), 8.53 (s, 1H), 8.37 (d, *J* = 8.6 Hz, 1H), 8.31 (d, *J* = 8.4 Hz, 1H), 7.74 (dt, *J* = 8.5, 2.4 Hz, 2H), 7.53 (d, *J* = 8.7 Hz, 1H), 7.39 (d, *J* = 8.8 Hz, 1H), 7.18 (d, *J* = 8.8 Hz, 1H), 7.10 (d, *J* = 8.7 Hz, 1H), 1.69 (s, 9H), 1.64 (s, 9H), 1.54 (s, 9H). ^{13}C NMR (101 MHz, $CDCl_3$) δ 149.81, 149.29, 147.29, 133.14, 132.96, 132.82, 130.49, 130.28, 130.21, 130.16, 130.00, 129.77, 129.73, 129.30, 129.27, 128.99, 128.60, 128.30, 127.71, 127.32, 127.25, 126.97, 126.49, 126.46, 126.05, 125.94, 125.86, 125.76, 125.46, 125.07, 124.95, 124.34, 123.62, 123.50, 121.83, 121.30, 120.97, 119.31, 118.35, 114.71, 35.47, 35.38, 35.33, 31.83, 31.79, 31.73.

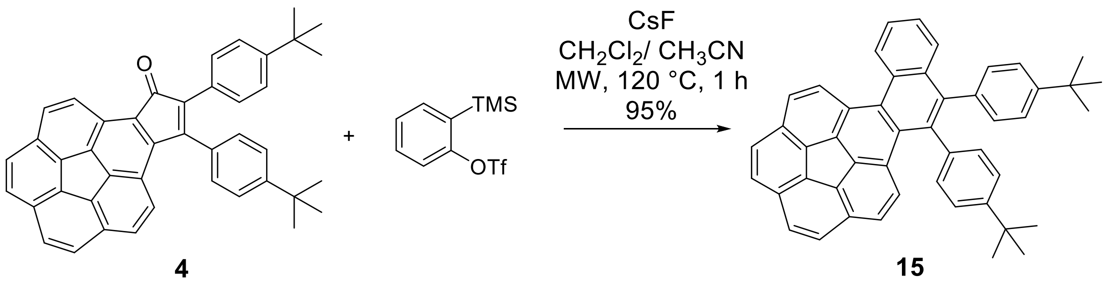

4 **15**

To a 10-mL microwave vial equipped with a magnetic stir bar was added **4** (11.3 mg, 0.02 mmol, 1 equiv), 2-(trimethylsilyl)phenyl trifluoromethanesulfonate (7.3 μL, 0.03 mmol, 1.5 equiv), and CsF (16.7 mg, 0.11 mmol, 5.5 equiv) in a mixture of CH_3CN (2 mL) and CH_2Cl_2 (1 mL). The vial was placed in the microwave reactor and heated to 120 ˚C with "heat as fast as possible to temperature" setting and a hold time of 1 hour. After cooling, solvent was removed under reduced pressure and the resulting crude residue was purified by flash column chromatography on silica gel (hexane/ CH_2Cl_2= 5:1) to afford **15** (11.7 mg, 95%) as yellowish white solid: HRMS (MALDI-TOF): m/z $[M]^+$ calcd for $C_{48}H_{34}$ 614.2968, found 614.2972. ^{1}H NMR (400 MHz, $CDCl_3$) δ 9.62 (d, *J* = 8.5 Hz, 1H), 8.72 (d, *J* = 8.8 Hz, 1H), 7.93 (d, *J* = 8.7 Hz, 1H), 7.85 (s, 2H), 7.83 – 7.76 (m, 3H), 7.73 (d, *J* = 8.7 Hz, 1H), 7.59 (ddd, *J* = 8.2, 6.7, 1.2 Hz, 1H), 7.36 (d, *J* = 9.1 Hz, 1H), 7.30 – 7.23 (m, 4H), 7.17 (d, *J* = 8.2 Hz, 2H), 7.03 (d, *J* = 8.0 Hz, 2H), 6.76 (d, *J* = 9.1 Hz, 1H), 1.35 (s, 9H), 1.32 (s, 9H). ^{13}C NMR (101 MHz, $CDCl_3$) δ 149.84, 149.21, 140.13, 139.83, 138.56, 138.20, 136.61, 136.24, 135.67, 134.97, 134.55, 132.77, 132.19, 131.48, 131.21, 131.00, 130.97, 130.88, 130.60, 129.79, 129.60, 129.50, 129.02, 127.87, 127.74, 127.52, 127.07, 126.95, 126.79, 126.63, 126.31, 126.12, 124.88, 124.33, 34.67, 34.59, 31.59, 31.52.

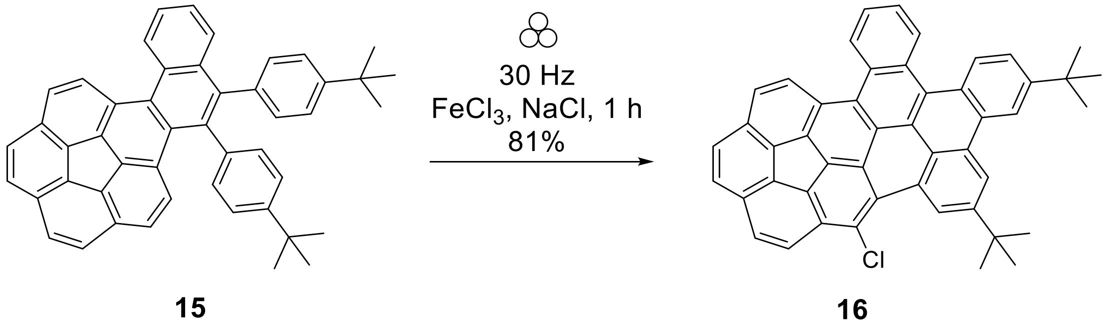

15 **16**

To a 15 mL ZrO_2 jar equipped with 1 piece of 10mm Ø ZrO_2 ball were added **15** (6.1 mg, 0.01 mmol, 1 equiv), anhydrous $FeCl_3$ (116.7 mg, 0.72 mmol, 72 equiv), and NaCl (1.527 g). The jar was placed into a glove box, assembled, and sealed with Teflon™ tape and parafilm. The jar was then transferred into the milling machine and milled for 1 hour at a frequency of 30 Hz. The crude mixture was dissolved in CH_2Cl_2

(10 mL) and washed with water. The aqueous layer was then extracted with CH_2Cl_2 (3 × 5 mL). The combined organic layers were washed with brine (5 mL), dried over Na_2SO_4, and evaporated under reduced pressure. The crude residue was purified by flash column chromatography on silica gel (hexane/CH_2Cl_2= 4:1) to afford **16** (5.2 mg, 81%) as orange solid: HRMS (MALDI-TOF): m/z $[M]^+$ calcd for $C_{48}H_{33}Cl$ 644.2265, found 644.2289. ^{1}H NMR (400 MHz, $CDCl_3$) δ 9.62 (d, J = 1.8 Hz, 1H), 9.54 (d, J = 8.2 Hz, 1H), 9.14 (d, J = 8.4 Hz, 1H), 8.94 (s, 1H), 8.78 (s, 1H), 8.66 (d, J = 8.7 Hz, 1H), 8.57 (d, J = 8.8 Hz, 1H), 8.11 (d, J = 8.9 Hz, 1H), 7.91 (dd, J = 7.5 Hz, 1H), 7.82 – 7.62 (m, 6H), 1.75 (s, 9H), 1.56 (s, 9H). ^{13}C NMR (101 MHz, $CDCl_3$) δ 149.83, 148.58, 136.65, 136.19, 135.27, 134.17, 132.09, 131.61, 131.10, 130.88, 130.77, 130.66, 130.64, 130.14, 129.68, 129.50, 129.44, 129.41, 129.15, 129.03, 128.68, 128.12, 127.96, 127.25, 126.97, 126.88, 126.73, 126.66, 126.00, 125.88, 125.78, 125.37, 125.22, 124.25, 123.33, 122.59, 119.57, 118.80, 35.72, 35.32, 31.87, 31.62.

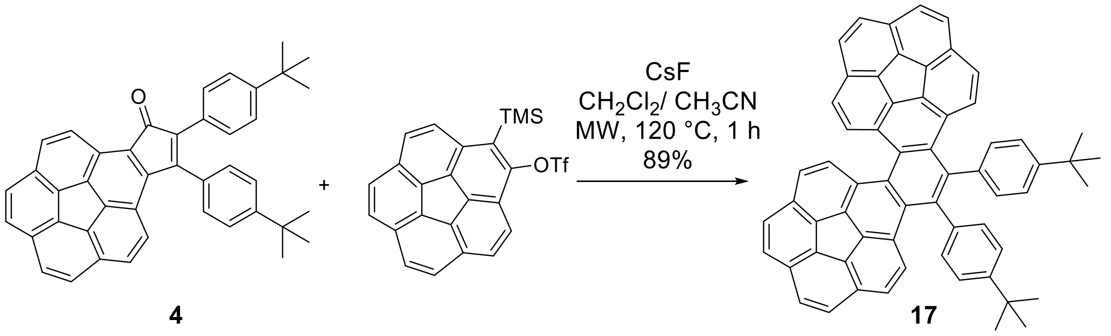

To a 10-mL microwave vial equipped with a magnetic stir bar was added **4** (11.3 mg, 0.02 mmol, 1 equiv), 2-trimethylsilylcorannulenyl triflate (14.1 mg, 0.03 mmol, 1.5 equiv), and CsF (16.7 mg, 0.11 mmol, 5.5 equiv) in a mixture of CH_3CN (2 mL) and CH_2Cl_2 (1 mL). The vial was placed in the microwave reactor and heated to 120 ˚C with "heat as fast as possible to temperature" setting and a hold time of 1 hour. After cooling, solvent was removed under reduced pressure and the resulting crude residue was purified by flash column chromatography on silica gel (hexane/ CH_2Cl_2= 5:1) to afford **17** (14 mg, 89%) as yellowish white solid: HRMS (MALDI-TOF): m/z $[M]^+$ calcd for $C_{62}H_{42}$ 786.3281, found 786.3303. ^{1}H NMR (400 MHz, $CDCl_3$) δ 8.91 (d, J = 8.8 Hz, 2H), 7.86 – 7.71 (m, 10H), 7.45 (ddd, J = 13.9, 9.5, 7.0 Hz, 6H), 7.14 (dd, J = 8.1, 2.0 Hz, 2H), 6.76 – 6.66 (m, 4H), 1.38 (s, 18H). ^{13}C NMR (101 MHz, $CDCl_3$) δ 150.10, 141.27, 139.46, 138.47, 136.89, 135.53, 135.39, 135.08, 132.96, 131.97,

131.63, 131.30, 130.97, 130.95, 130.91, 130.85, 130.58, 129.41, 128.84, 127.54, 127.20, 126.91, 126.88, 126.55, 126.27, 125.10, 124.98, 34.73, 31.63.

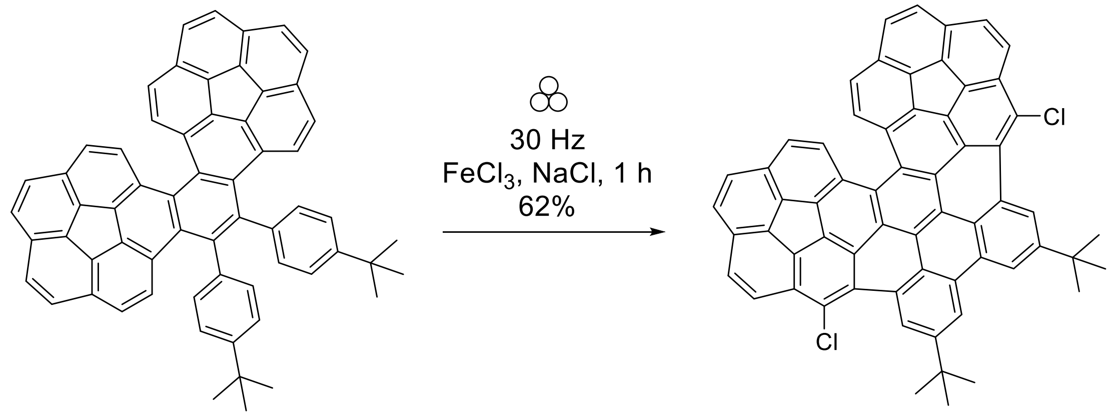

17 18

To a 15 mL ZrO_2 jar equipped with 1 piece of 10mm Ø ZrO_2 ball were added **17** (7.8 mg, 0.01 mmol, 1 equiv), anhydrous $FeCl_3$ (116.7 mg, 0.72 mmol, 72 equiv), and NaCl (1.525 g). The jar was placed into a glove box, assembled, and sealed with Teflon™ tape and parafilm. The jar was then transferred into the milling machine and milled for 1 hour at a frequency of 30 Hz. The crude mixture was dissolved in CH_2Cl_2 (10 mL) and washed with water. The aqueous layer was then extracted with CH_2Cl_2 (3 × 5 mL). The combined organic layers were washed with brine (5 mL), dried over Na_2SO_4, and evaporated under reduced pressure. The crude residue was purified by flash column chromatography on silica gel (hexane/CH_2Cl_2= 1:1) to afford 17 (3.9 mg, 62%) as orange solid: HRMS (MALDI-TOF): m/z $[M]^+$ calcd for $C_{62}H_{34}Cl_2$ 848.2032, found 848.2041. ^{1}H NMR (400 MHz, $CDCl_3$) δ 10.04 (s, 2H), 9.37 (d, *J* = 8.8 Hz, 2H), 9.21 (s, 2H), 8.34 (d, *J* = 8.8 Hz, 2H), 7.93 – 7.86 (m, 4H), 7.81 (s, 4H), 1.78 (s, 18H). ^{13}C NMR (100 MHz, $C_2D_2Cl_4$) δ 152.07, 140.27, 139.82, 138.61, 136.88, 135.14, 134.81, 134.21, 134.05, 133.82, 133.60, 133.58, 133.11, 132.82, 131.40, 131.38, 131.25, 131.03, 130.58, 130.56, 130.35, 130.33, 130.27, 130.24, 129.59, 129.56, 129.18, 129.15, 128.67, 127.81, 127.28, 127.25, 126.05, 123.23, 34.93, 32.78.

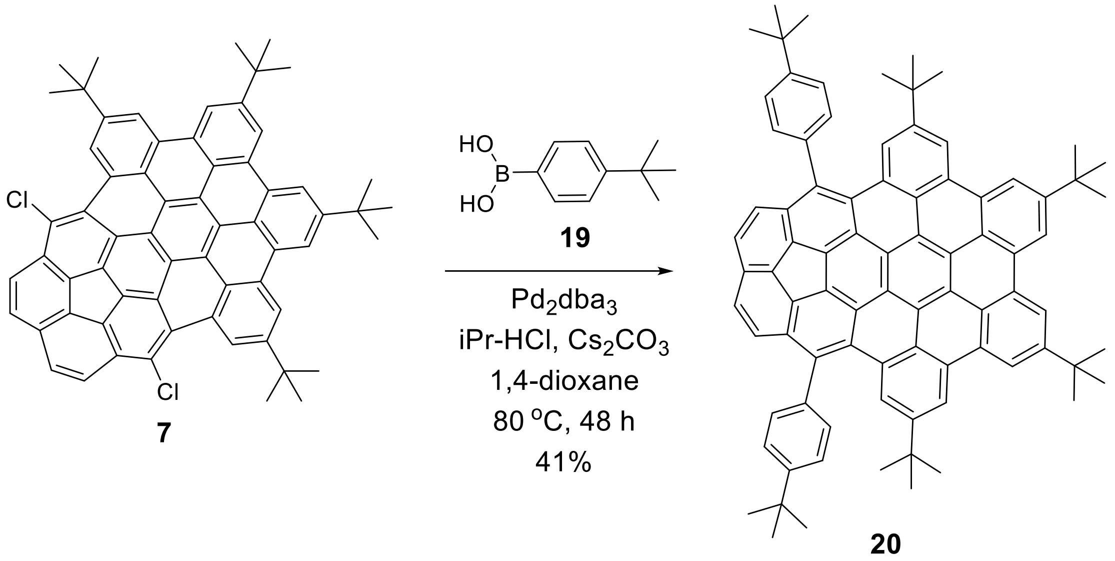

To an oven dried Schlenk tube was added dichlorocorannulene derivatives **7** (8.9 mg, 0.01 mmol, 1 equiv), 4-*t*-butylphenylboronic acid **19** (14.2 mg, 0.08 mmol, 8 equiv), Cs_2CO_3 (52.2 mg, 0.16 mmol, 16 equiv), 1,3-bis(2,6-di-i-propylphenyl)imidazolium chloride (1.0 mg, 2.4 μmol, 0.24 equiv) and tris(dibenzylideneacetone)dipalladium (1.1 mg, 1.2μ mmol, 0.12 equiv). The tube was repeatedly purged with argon and anhydrous 1,4-dioxane (0.5 mL) was then added to the solid mixture. The resulting solution was stirred at 80 °C for 48 hours before being cooled down to room temperature. The reaction mixture was filtered through a short pad of silica gel and washed with dichloromethane. The filtrate was concentrated under reduced pressure and the crude residue was purified by preparative layer chromatography (PLC) (hexane/$C_2H_4Cl_2$= 4:1) to afford **20** (4.4 mg, 41%) as brown solid: HRMS (MALDI-TOF): m/z $[M]^+$ calcd for $C_{84}H_{74}$ 1082.5785, found 1082.5739. ^{1}H NMR (400 MHz, $CDCl_3$) δ 9.22 (d, *J* = 3.5 Hz, 4H), 9.08 (s, 2H), 8.52 (d, *J* = 1.7 Hz, 2H), 8.43 (dd, *J* = 8.0, 2.0 Hz, 2H), 7.94 (dd, *J* = 8.0, 2.1 Hz, 2H), 7.66 (d, *J* = 8.9 Hz, 2H), 7.49 (d, *J* = 8.9 Hz, 2H), 7.19 (dd, *J* = 8.1, 2.1 Hz, 2H), 6.54 (dd, *J* = 8.2, 2.0 Hz, 2H), 1.78 (s, 18H), 1.45 (s, 18H), 1.38 (s, 18H). ^{13}C NMR (101 MHz, $CDCl_3$) δ 150.26, 149.01, 147.79, 138.37, 137.46, 137.14, 136.48, 134.72, 134.10, 131.86, 131.55, 130.87, 130.80, 130.50, 130.26, 130.02, 128.86, 128.05, 126.98, 126.29, 126.21, 125.51, 123.51, 120.87, 120.69, 119.06, 118.71, 118.37, 35.79, 35.17, 34.74, 32.04, 31.56, 31.49.

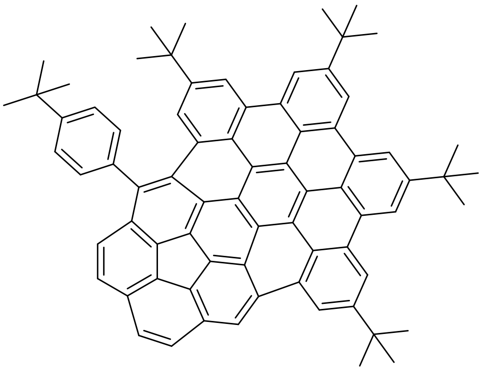

Trace side-product **22**: HRMS (MALDI-TOF): m/z $[M]^+$ calcd for $C_{74}H_{62}$ 950.4846, found 950.4901. ^{1}H NMR (400 MHz, $CDCl_3$) δ 9.27 – 9.18 (m, 6H), 9.06 (s, 1H), 8.90 (s, 1H), 8.49 (s, 1H), 8.43 (dd, *J* = 7.9, 2.0 Hz, 1H), 8.11 (d, *J* = 8.8 Hz, 1H), 7.94 (dd, *J* = 8.0, 2.1 Hz, 1H), 7.82 (d, *J* = 8.8 Hz, 1H), 7.69 (d, *J* = 8.9 Hz, 1H), 7.49 (d, *J* = 8.9 Hz, 1H), 7.16 (dd, *J* = 8.2, 2.1 Hz, 1H), 6.55 – 6.48 (m, 1H), 1.84 (s, 9H), 1.80 (s, 9H), 1.77 (s, 9H), 1.44 (s, 9H), 1.38 (s, 9H). A reasonable ^{13}C-NMR could not be obtained due to low concentration.

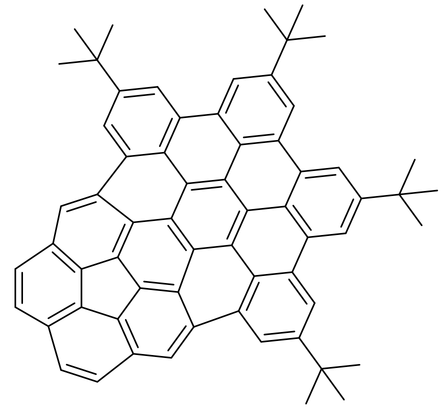

Trace side-product **23**: HRMS (MALDI-TOF): m/z $[M]^+$ calcd for $C_{64}H_{50}$ 818.3907, found 818.3929. ^{1}H NMR (400 MHz, $CDCl_3$) δ 9.11 (d, *J* = 1.3 Hz, 4H), 9.03 (s, *J* = 7.2 Hz, 2H), 8.92 (s, 2H), 8.47 (s, 2H), 7.81 (d, *J* = 8.5 Hz, 2H), 7.61 (d, *J* = 8.6 Hz, 2H), 1.80 (s, 18H), 1.79 (s, 18H). ^{13}C NMR (100 MHz, $CDCl_3$) δ 149.29, 148.87, 135.87, 135.61, 134.46, 132.85, 132.06, 130.73, 130.68, 130.32, 129.76, 128.13, 127.30, 126.68, 124.38, 123.55, 121.39, 120.64, 120.55, 120.39, 119.38, 119.04, 118.83, 35.85, 35.76, 32.16, 32.10.

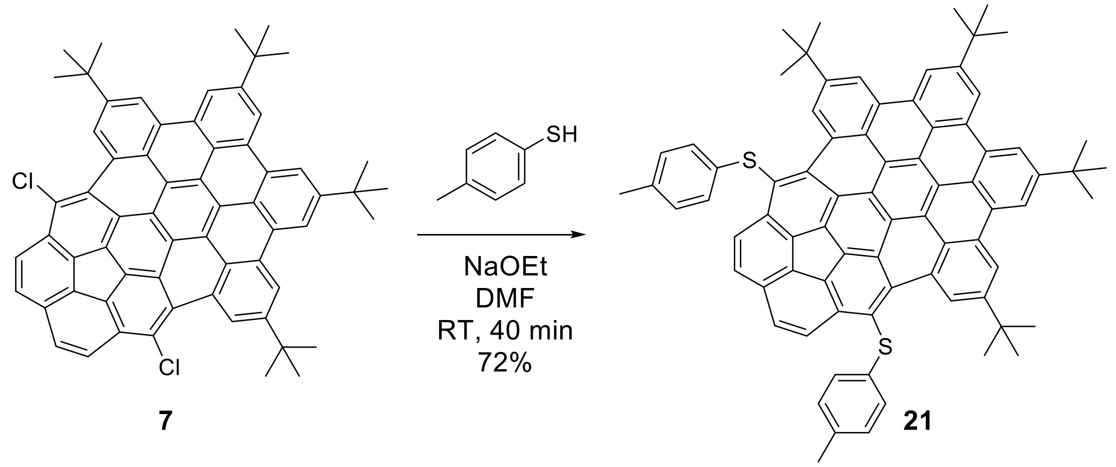

7 **21**

To a freshly prepared solution of NaOEt by adding sodium (1 mg, 0.04 mmol, 4 equiv) to EtOH (20 μL) under argon atmosphere was added thiocresol (5.4 mg, 0.04 mmol, 4 equiv). The resulting mixture was stirred for 40 minutes at room temperature. The solution was subsequently concentrated, the residue was dissolved in DMF (0.5 mL) and dichlorocorannulene derivative **7** (10 mg, 0.01 mmol) was added to the resulting solution. The reaction mixture was stirred for 2 days under argon before being diluted with 10 mL toluene and washed with 3x5 mL water. The organic phase was dried over anhydrous $MgSO_4$, filtered and concentrated. The crude product was purified with flash column chromatography on silica gel (hexane/CH_2Cl_2= 2:1) to afford **21** (8.6 mg, 72%) as red solid: HRMS (MALDI-TOF): m/z $[M]^+$ calcd for $C_{78}H_{62}S_2$ 1062.4288, found 1062.4373. ^1H NMR (400 MHz, $CDCl_3$) δ 10.13 (d, *J* = 1.7 Hz, 2H), 9.20 – 9.12 (m, 7H), 8.41 (d, *J* = 8.9 Hz, 2H), 7.77 (d, *J* = 9.0 Hz, 2H), 7.08 (d, *J* = 8.1 Hz, 4H), 2.30 (s, 6H), 1.78 (s, 18H), 1.59 (s, 18H). ^{13}C NMR (101 MHz, $CDCl_3$) δ 149.33, 148.44, 137.84, 137.38, 136.38, 136.28, 135.31, 134.95, 131.30, 130.96, 130.61, 130.22, 130.13, 128.85, 128.10, 127.59, 126.39, 126.28, 125.56, 125.41, 123.29, 121.19, 120.68, 120.07, 119.88, 119.64, 119.15, 118.87, 35.96, 35.78, 32.21, 31.71, 29.86.

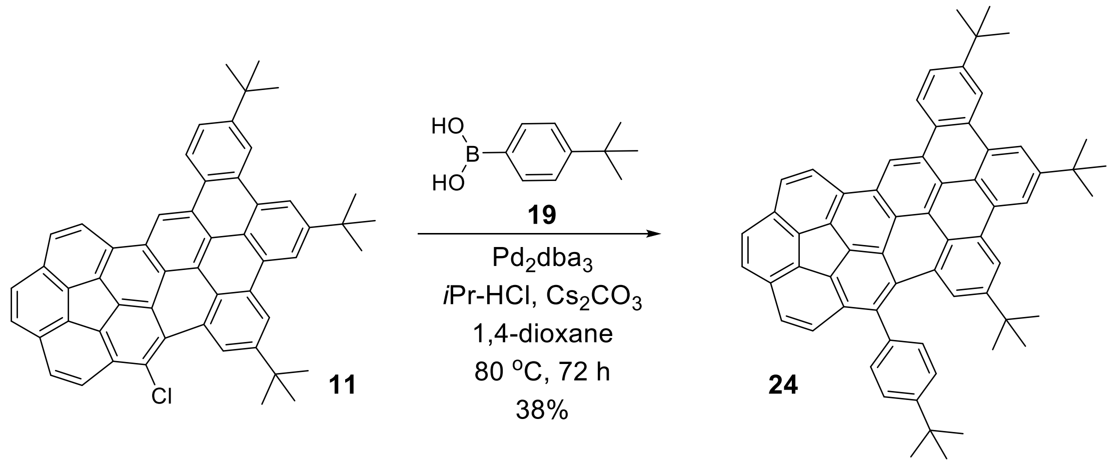

To an oven dried pressure vessel was added dichlorocorannulene derivative **11** (6.9 mg, 0.01 mmol, 1 equiv), 4-*t*-butylphenylboronic acid **19** (14.0 mg, 0.08 mmol, 8 equiv), Cs_2CO_3 (50.0 mg, 0.14 mmol, 15 equiv), 1,3-bis(2,6-di-i-propylphenyl)imidazolium chloride (1.5 mg, 3.5 μmol, 0.35 equiv) and tris(dibenzylideneacetone)dipalladium (1.5 mg, 1.6 μmol, 0.2 equiv). The tube was repeatedly purged with argon and anhydrous 1,4-dioxane (0.7 mL) was then added to the solid mixture. The resulting solution was stirred at 80 °C for 72 hours before being cooled down to room temperature. The reaction mixture was filtered through a short pad of silica gel and washed with dichloromethane. The filtrate was concentrated under reduced pressure and the crude residue was purified by preparative layer chromatography (PLC) (hexane/$C_2H_4Cl_2$= 4:1) to afford **24** (3.0 mg, 38%) as yellow solid: HRMS (MALDI-TOF): m/z $[M]^+$ calcd for $C_{64}H_{54}$ 822.4220, found 822.4216. ^{1}H NMR (400 MHz, $CDCl_3$ -40 °C) δ 10.21 (s, 1H), 9.19 – 9.12 (m, 2H), 9.04 (d, *J* = 7.4 Hz, 2H), 8.89 (s, 1H), 8.72 (d, *J* = 8.4 Hz, 1H), 8.47 (s, 1H), 8.35 (d, *J* = 8.4 Hz, 1H), 8.14 (d, *J* = 8.6 Hz, 1H), 7.98 – 7.88 (m, 3H), 7.83 (d, *J* = 8.4 Hz, 1H), 7.73 (d, *J* = 9.1 Hz, 1H), 7.57 (d, *J* = 9.0 Hz, 1H), 7.21 (d, *J* = 5.8 Hz, 1H), 6.62 (d, *J* = 8.9 Hz, 1H), 1.61 (s, 9H), 1.42 (s, 9H), 1.32 (s, 9H). ^{13}C NMR (100 MHz, $CDCl_3$) δ 150.65, 150.48, 149.27, 147.92, 138.31, 137.89, 137.84, 137.02, 136.15, 135.14, 133.31, 132.74, 131.84, 131.33, 131.02, 130.79, 130.50, 130.49, 130.46, 130.32, 130.02, 129.08, 128.78, 128.51, 127.90, 127.81, 127.63, 127.39, 127.04, 126.56, 125.78, 125.60, 125.41, 125.07, 124.03, 123.85, 122.68, 122.18, 119.81, 119.02, 118.63, 118.46, 116.77, 35.85, 35.38, 35.24, 34.90, 32.08, 31.70, 31.65, 31.62.

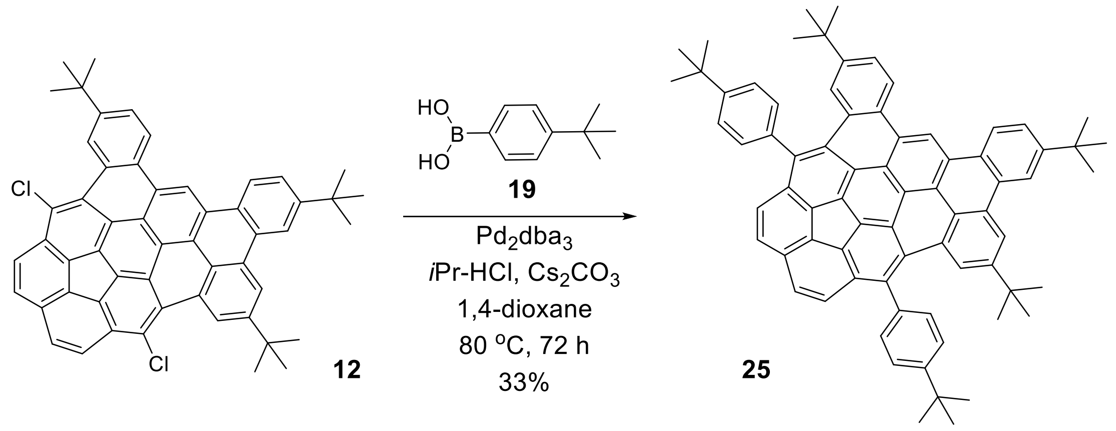

To an oven dried pressure vessel was added dichlorocorannulene derivative **12** (8.2 mg, 0.01 mmol, 1 equiv), 4-*t*-butylphenylboronic acid **19** (15.4 mg, 0.08 mmol, 8 equiv), Cs_2CO_3 (56.3 mg, 0.17 mmol, 16 equiv), 1,3-bis(2,6-di-i-propylphenyl)imidazolium chloride (1.4 mg, 3.3 μmol, 0.33 equiv) and tris(dibenzylideneacetone)dipalladium (1.2 mg, 1.4 μmol, 0.2 equiv). The tube was repeatedly purged with argon and anhydrous 1,4-dioxane (0.7 mL) was then added to the solid mixture. The resulting solution was stirred at 80 °C for 72 hours before being cooled down to room temperature. The reaction mixture was filtered through a short pad of silica gel and washed with dichloromethane. The filtrate was concentrated under reduced pressure and the crude residue was purified by preparative layer chromatography (PLC) (hexane/$C_2H_4Cl_2$= 4:1) to afford **25** (3.4 mg, 33%) as yellow solid: HRMS (MALDI-TOF): m/z $[M]^+$ calcd for $C_{74}H_{66}$ 954.5159, found 954.5213. ^1H NMR (400 MHz, $CDCl_3$) δ 9.97 (s, 1H), 9.05 (d, *J* = 8.6 Hz, 1H), 8.94 (d, *J* = 8.6 Hz, 1H), 8.87 (d, *J* = 1.6 Hz, 2H), 8.42 (d, *J* = 1.5 Hz, 1H), 8.35 (ddd, *J* = 14.0, 8.0, 1.9 Hz, 2H), 8.11 (d, *J* = 2.0 Hz, 1H), 7.93 – 7.83 (m, 3H), 7.72 (dd, *J* = 8.4, 2.0 Hz, 1H), 7.62 (d, *J* = 9.0 Hz, 2H), 7.45 (dd, *J* = 11.4, 9.0 Hz, 2H), 7.24 (dd, *J* = 8.1, 2.2 Hz, 1H), 7.17 (dd, *J* = 8.1, 2.0 Hz, 1H), 6.66 (dd, *J* = 8.1, 1.9 Hz, 1H), 6.48 (dd, *J* = 8.1, 1.9 Hz, 1H), 1.59 (s, 9H), 1.44 (s, 18H), 1.32 (s, 9H), 1.19 (s, 9H). ^{13}C NMR (100 MHz, $CDCl_3$) δ 150.48, 150.40, 150.38, 149.12, 147.72, 138.35, 137.83, 137.55, 137.44, 137.31, 136.56, 136.35, 134.73, 134.26, 134.17, 134.12, 132.69, 131.71, 131.61, 131.27, 131.23, 130.98, 130.85, 130.78, 130.42, 130.14, 129.80, 129.33, 129.05, 128.96, 128.13, 128.07, 128.06, 127.92, 127.84, 127.45, 127.36, 127.13, 126.42, 126.32, 126.13, 125.46, 124.76, 124.72, 124.54, 124.26, 123.77, 123.18, 122.84, 119.87, 118.16, 114.97, 35.34, 35.21, 34.87, 34.74, 31.66, 31.63, 31.61, 31.29.

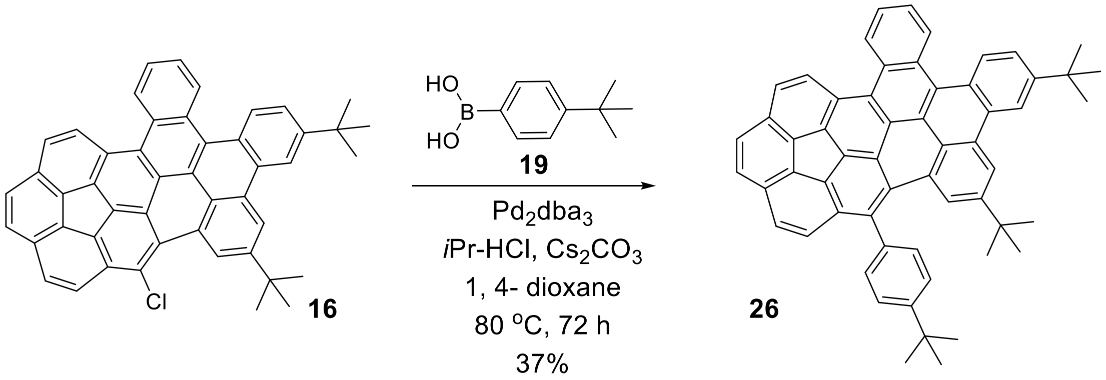

To an oven dried pressure vessel was added dichlorocorannulene derivative **16** (8.8 mg, 0.01 mmol, 1 equiv), 4-*t*-butylphenylboronic acid **19** (9.7 mg, 0.05 mmol, 5 equiv), Cs_2CO_3 (35.5 mg, 0.11 mmol, 11 equiv), 1,3-bis(2,6-di-i-propylphenyl)imidazolium chloride (1.4 mg, 3.3 μmol, 0.33 equiv) and tris(dibenzylideneacetone)dipalladium (1.1 mg, 2.6 μmol, 0.2 equiv). The tube was repeatedly purged with argon and anhydrous 1,4-dioxane (0.7 mL) was then added to the solid mixture. The resulting solution was stirred at 80 °C for 72 hours before being cooled down to room temperature. The reaction mixture was filtered through a short pad of silica gel and washed with dichloromethane. The filtrate was concentrated under reduced pressure and the crude residue was purified by preparative layer chromatography (PLC) (hexane/$C_2H_4Cl_2$= 4:1) to afford **26** (3.7 mg, 37%) as yellow solid: HRMS (MALDI-TOF): m/z $[M]^+$ calcd for $C_{58}H_{46}$ 742.3594, found 742.3648. ^{1}H NMR (400 MHz, $CDCl_3$ -40 ^{o}C) δ 9.78 (d, *J* = 8.2 Hz, 1H), 9.27 (d, *J* = 8.2 Hz, 1H), 8.84 (dd, *J* = 15.9, 9.0 Hz, 2H), 8.76 (d, *J* = 10.6 Hz, 2H), 8.30 (d, *J* = 9.8 Hz, 1H), 8.24 (s, 1H), 8.06 – 7.96 (m, 2H), 7.90 – 7.83 (m, 4H), 7.80 – 7.71 (m, 2H), 7.57 (d, *J* = 9.1 Hz, 1H), 7.15 (d, 1H), 6.58 (d, *J* = 8.3 Hz, 1H), 1.39 (s, 9H), 1.24 (s, 9H). ^{13}C NMR (100 MHz, $CDCl_3$) δ 150.44, 149.88, 148.19, 138.21, 138.11, 137.41, 136.77, 135.93, 135.04, 133.67, 133.51, 131.90, 131.37, 131.30, 131.21, 131.13, 130.34, 129.89, 129.67, 129.61, 129.50, 129.43, 129.23, 128.79, 128.65, 128.37, 128.17, 127.48, 127.32, 127.22, 127.08, 126.66, 126.43, 125.99, 125.95, 125.52, 124.95, 124.50, 124.27, 123.25, 119.65, 117.85, 35.34, 35.07, 34.87, 31.65, 31.63, 31.50.

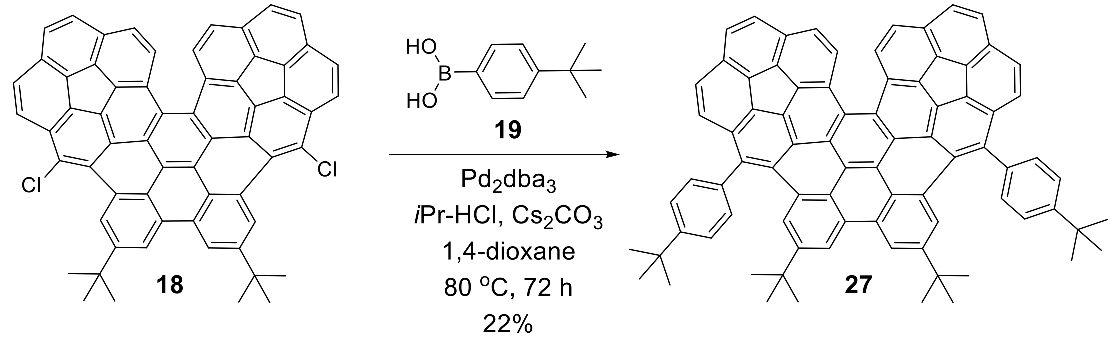

To an oven dried pressure vessel was added dichlorocorannulene derivative **18** (18.0 mg, 0.02 mmol, 1 equiv), 4-*t*-butylphenylboronic acid **19** (30.0 mg, 0.16 mmol, 16 equiv), Cs_2CO_3 (100.0 mg, 0.30 mmol, 30 equiv), 1,3-bis(2,6-di-i-propylphenyl)imidazolium chloride (3.0 mg, 7.0 μmol, 0.7 equiv) and tris(dibenzylideneacetone)dipalladium (2.0 mg, 2.2 μmol, 0.2 equiv). The tube was repeatedly purged with argon and anhydrous 1,4-dioxane (0.7 mL) was then added to the solid mixture. The resulting solution was stirred at 80 °C for 72 hours before being cooled down to room temperature. The reaction mixture was filtered through a short pad of silica gel and washed with dichloromethane. The filtrate was concentrated under reduced pressure and the crude residue was purified by preparative layer chromatography (PLC) (hexane/$C_2H_4Cl_2$= 4:1) to afford **27** (4.9 mg, 22%) as yellow solid: HRMS (MALDI-TOF): m/z $[M]^+$ calcd for $C_{82}H_{60}$ 1044.4689, found 1044.4673. ^{1}H NMR (400 MHz, $CDCl_3$) δ 9.58 (d, *J* = 8.8 Hz, 2H), 8.98 (s, 2H), 8.57 (s, 2H), 8.35 (dd, *J* = 7.9, 2.0 Hz, 2H), 8.01 (d, *J* = 8.8 Hz, 2H), 7.94 (dd, *J* = 8.0, 2.0 Hz, 2H), 7.87 (dd, *J* = 8.7 Hz, 4H), 7.76 (d, *J* = 9.0 Hz, 2H), 7.54 (d, *J* = 8.9 Hz, 3H), 7.31 (dd, *J* = 8.1, 1.9 Hz, 2H), 6.72 (dd, *J* = 8.1, 2.0 Hz, 2H), 1.48 (s, 18H), 1.30 (s, 18H). ^{13}C NMR (100 MHz, $CDCl_3$) δ 150.51, 147.96, 139.00, 138.73, 138.19, 137.37, 136.25, 134.95, 133.68, 133.63, 132.07, 131.53, 131.39, 131.37, 131.33, 131.05, 130.92, 130.58, 130.00, 128.78, 128.72, 128.31, 127.95, 127.66, 127.51, 127.27, 126.72, 126.66, 125.64, 125.37, 124.76, 123.41, 119.31, 35.20, 34.94, 31.67, 31.55.

Solution-phase Scholl reactions:

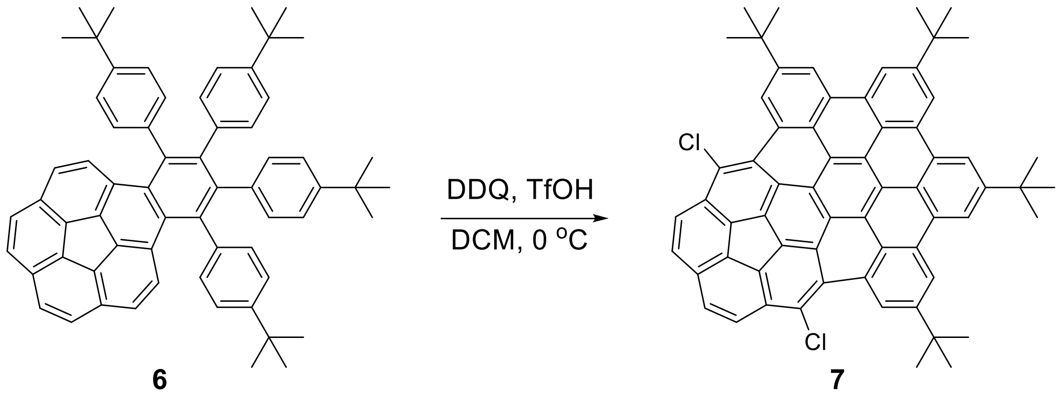

6 **7**

To a dry round bottom flask equipped with a magnetic stir bar compound **6** (20.0 mg, 0.024 mmol, 1 equiv) was dissolved in dry CH_2Cl_2 (10 mL). The solution was cooled at 0 °C and bubbled with Ar during the reaction. Afterwards, DDQ (239.7 mg, 1.056 mmol, 44 equiv) was added in one portion, followed by the addition of TfOH dropwise (2.0 mL). The resulting mixture was stirred for 20 minutes. After that, the mixture was quenched with H_2O and was extracted with CH_2Cl_2. The combined organic layers were dried over Na_2SO_4, filtered and concentrated under vacuum. The crude residue was purified through flash column chromatography (hexane:CH_2Cl_2, 1:1).

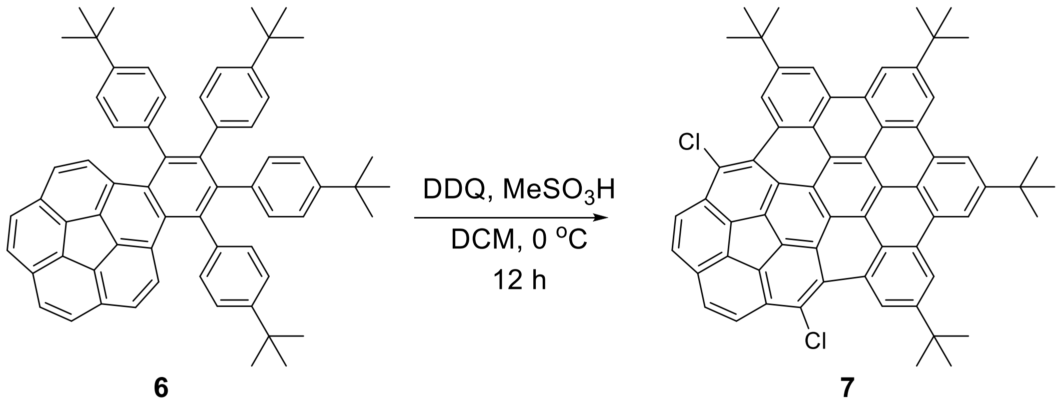

6 **7**

To a dry round bottom flask equipped with a magnetic stir bar compound **6** (10.0 mg, 0.012 mmol, 1 equiv)) was dissolved in dry CH_2Cl_2 (9.6 ml). The solution was cooled at 0 °C, bubbled with Ar and $MeSO_3H$ (2.1 ml) was slowly added. Afterwards, DDQ (13.6 mg, 0.06 mmol, 5 equiv)) was added in one portion and mixture was stirred for 12 hours at 0 °C. After that, the mixture was quenched with aqueous solution of $NaHCO_3$ and was extracted with CH_2Cl_2. The combined organic layers were dried over Na_2SO_4, filtered and concentrated under vacuum. The crude residue was purified by preparative layer chromatography (PLC) (hexane:CH_2Cl_2, 1:1).

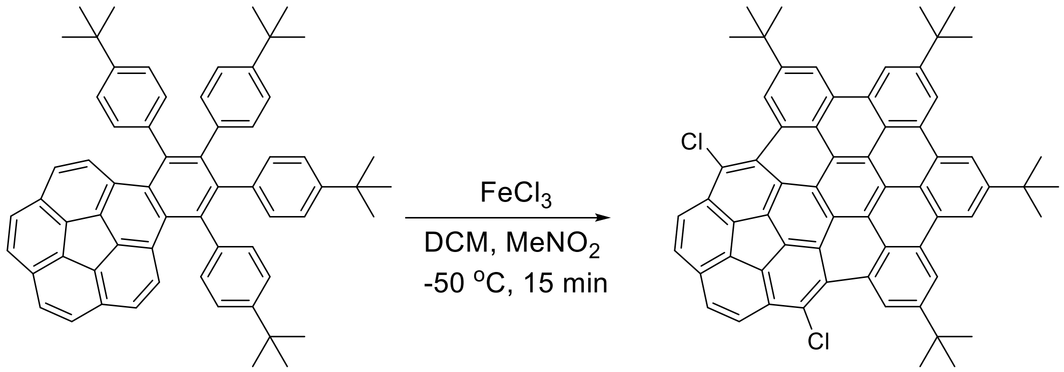

6 **7**

To a dry round bottom flask equipped with a magnetic stir bar compound **6** (10 mg, 0.012 mmol, 1 equiv) was dissolved in dry CH_2Cl_2 (5 ml). The solution was cooled at -50 °C and bubbled with Ar during the reaction. Afterwards, a solution of $FeCl_3$ (78 mg, 0.48 mmol, 40 equiv) in $MeNO_2$ (2.5 mL) was added dropwise. The resulting mixture was stirred for 15 minutes, then washed with H_2O and extracted with CH_2Cl_2. The combined organic layers were dried over Na_2SO_4, filtered and concentrated under vacuum. The crude residue was purified through column chromatography (hexane:CH_2Cl_2 = 9:1).

Supplementary Table

Supplementary Table 1. Bowl-depth for the prepared compounds.

**Compound**	**Bowl-depth**
**4**	0.851 Å
**6**	0.811 Å
**7**	0.923 Å
**10**	0.809 Å
**15**	0.856 Å
**17** (*M*)	0.880, 0.833 Å
**17** (*P*)	0.882, 0.800 Å
**20**	0.959 Å
**22**	1.005 Å
**23**	1.005 Å
**24**	0.899 Å
**25**	1.012 Å
**26**	0.947 Å
**27**	0.908 Å

Supplementary Figures

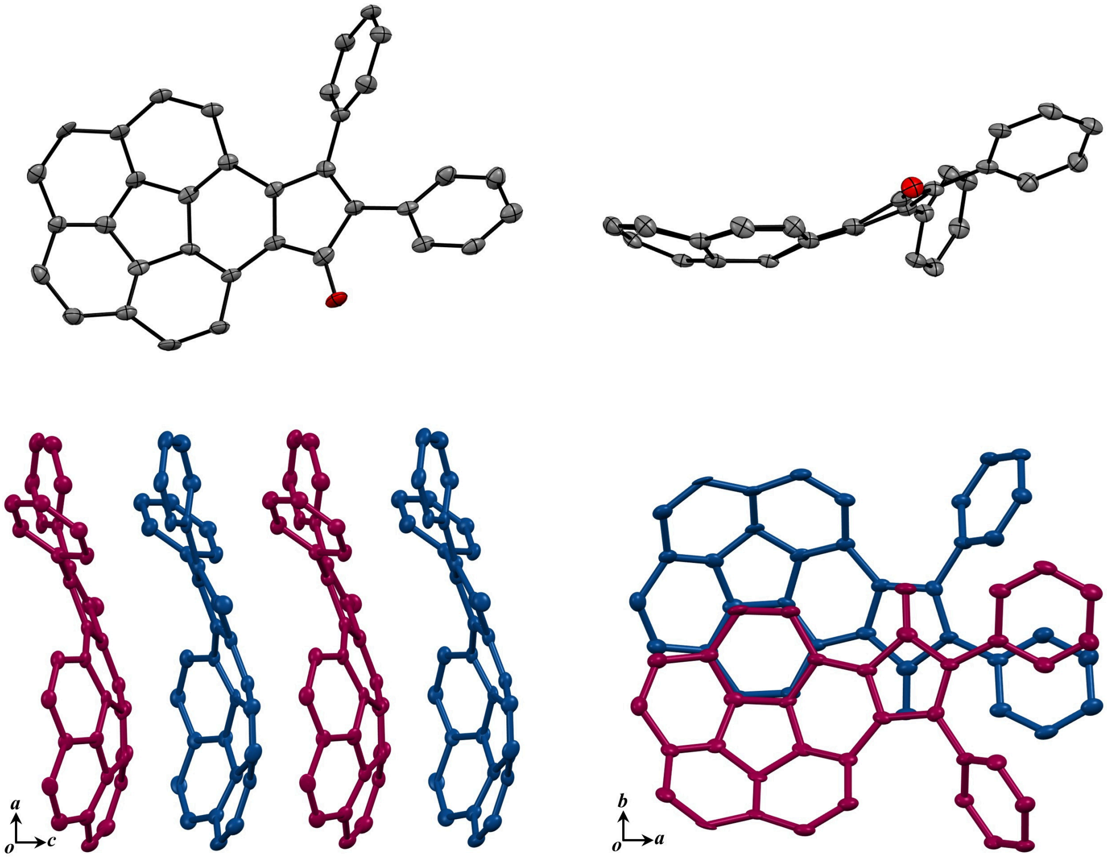

Supplementary Figure 1. X-Ray crystal structure of **4**. Thermal ellipsoids were scaled at 50% probability level. Top view (top left), side view (top right); packing structure (bottom), *tert*-butyl groups and hydrogen atoms were omitted for clarity.

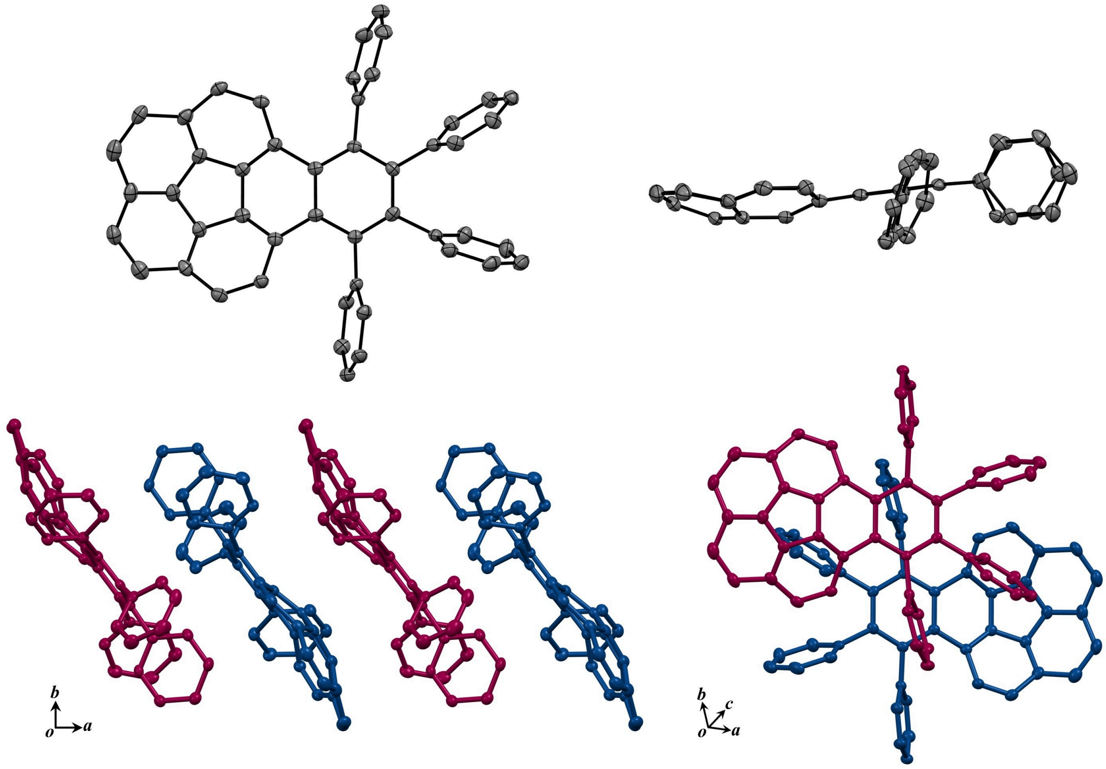

Supplementary Figure 2. X-Ray crystal structure of **6**. Thermal ellipsoids were scaled at 50% probability level. Top view (top left), side view (top right); packing structure (bottom), *tert*-butyl groups and hydrogen atoms were omitted for clarity.

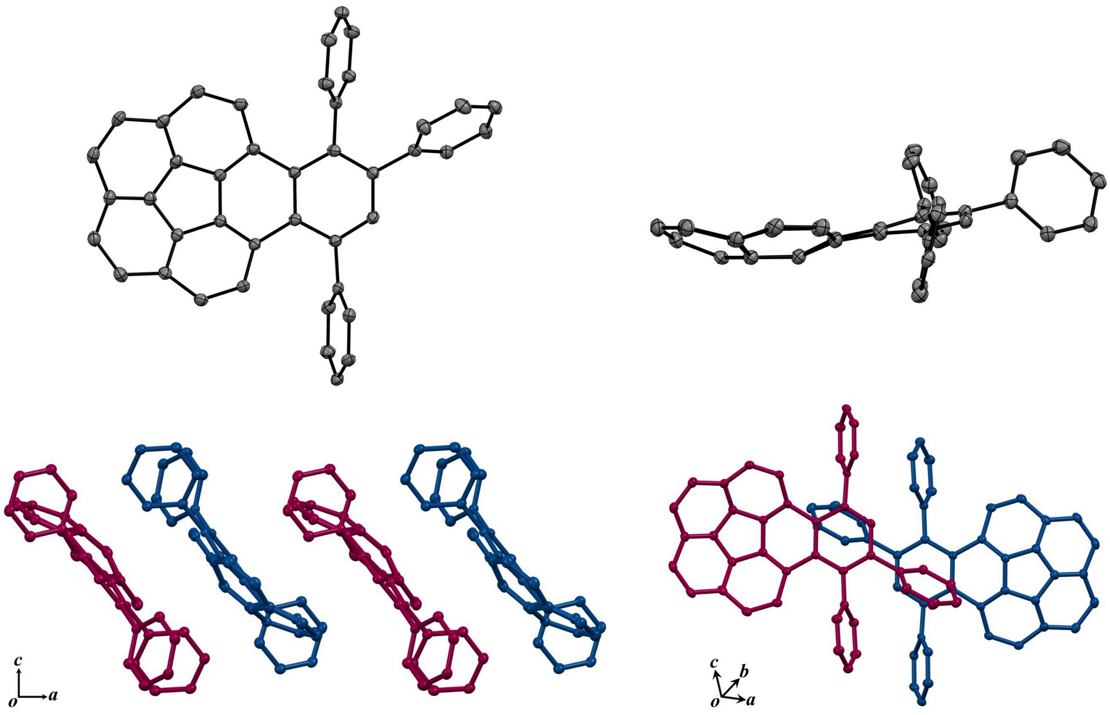

Supplementary Figure 3. X-Ray crystal structure of **10**. Thermal ellipsoids were scaled at 50% probability level. Top view (top left), side view (top right); packing structure (bottom), *tert*-butyl groups and hydrogen atoms were omitted for clarity.

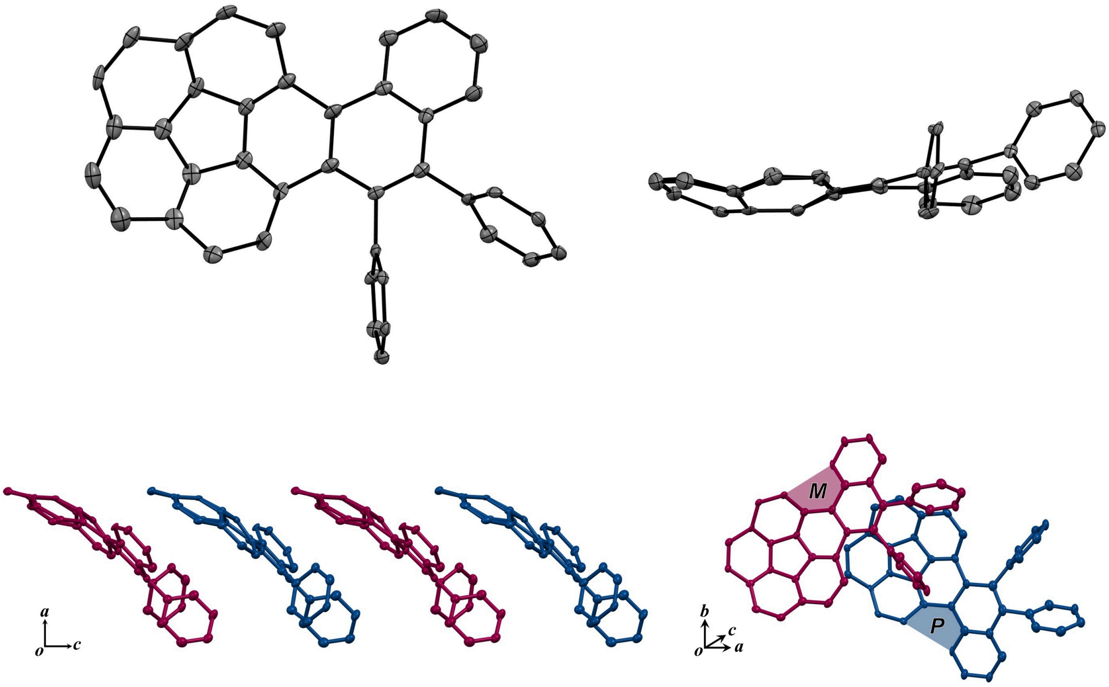

Supplementary Figure 4. X-Ray crystal structure of **15**. Thermal ellipsoids were scaled at 50% probability level. Top view (top left), side view (top right); packing structure (bottom), *tert*-butyl groups and hydrogen atoms were omitted for clarity.

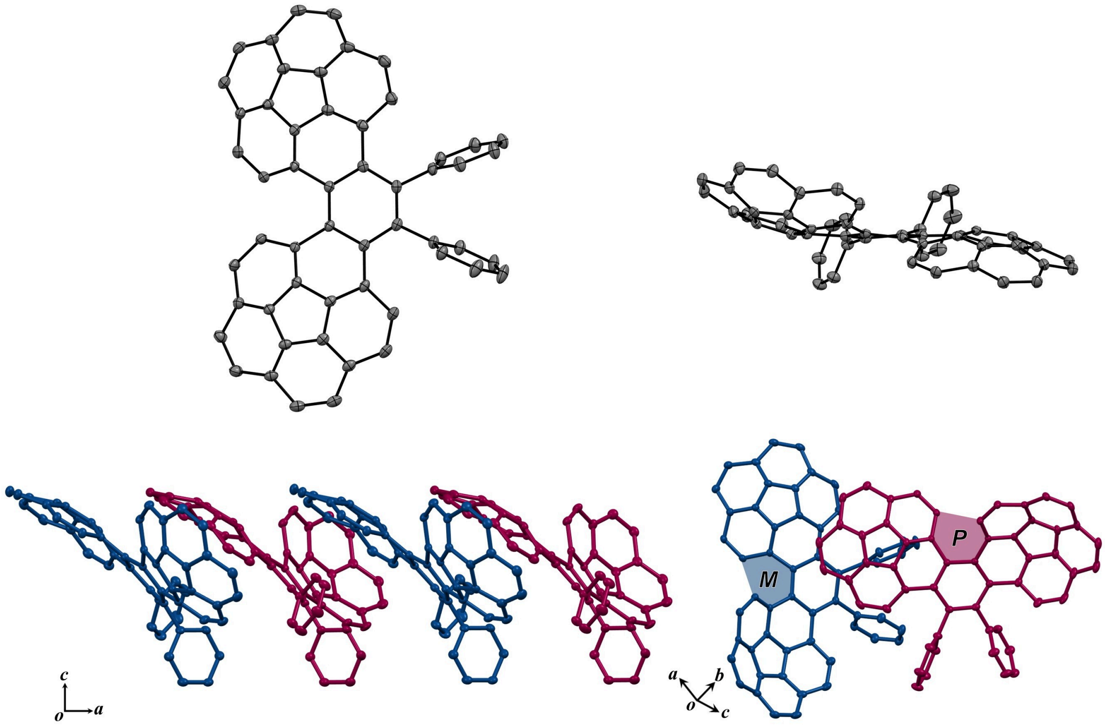

Supplementary Figure 5. X-Ray crystal structure of **17**. Thermal ellipsoids were scaled at 50% probability level. Top view (top left), side view (top right); packing structure (bottom), *tert*-butyl groups and hydrogen atoms were omitted for clarity.

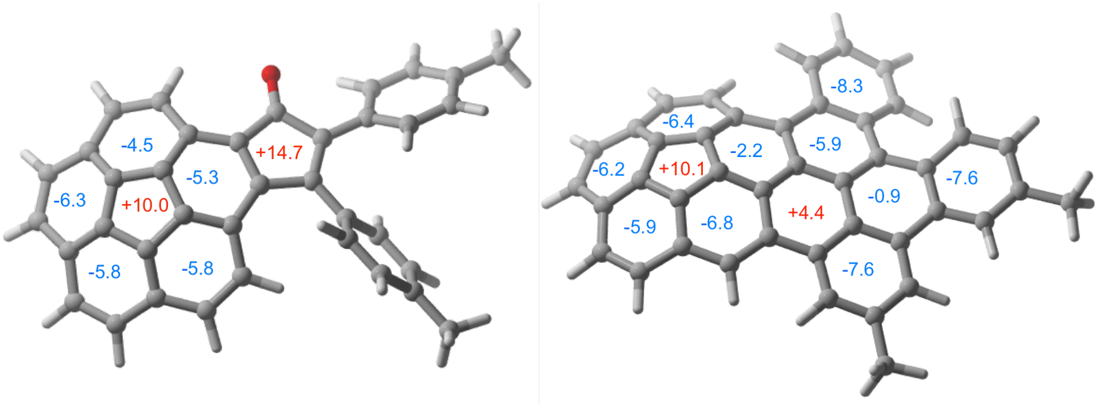

Supplementary Figure 6. NICS values (in ppm) are shown in the optimized structures for **4’** and **16’**. Negative (aromatic) values are given in blue whereas positive (anti-aromatic) values are given in red.

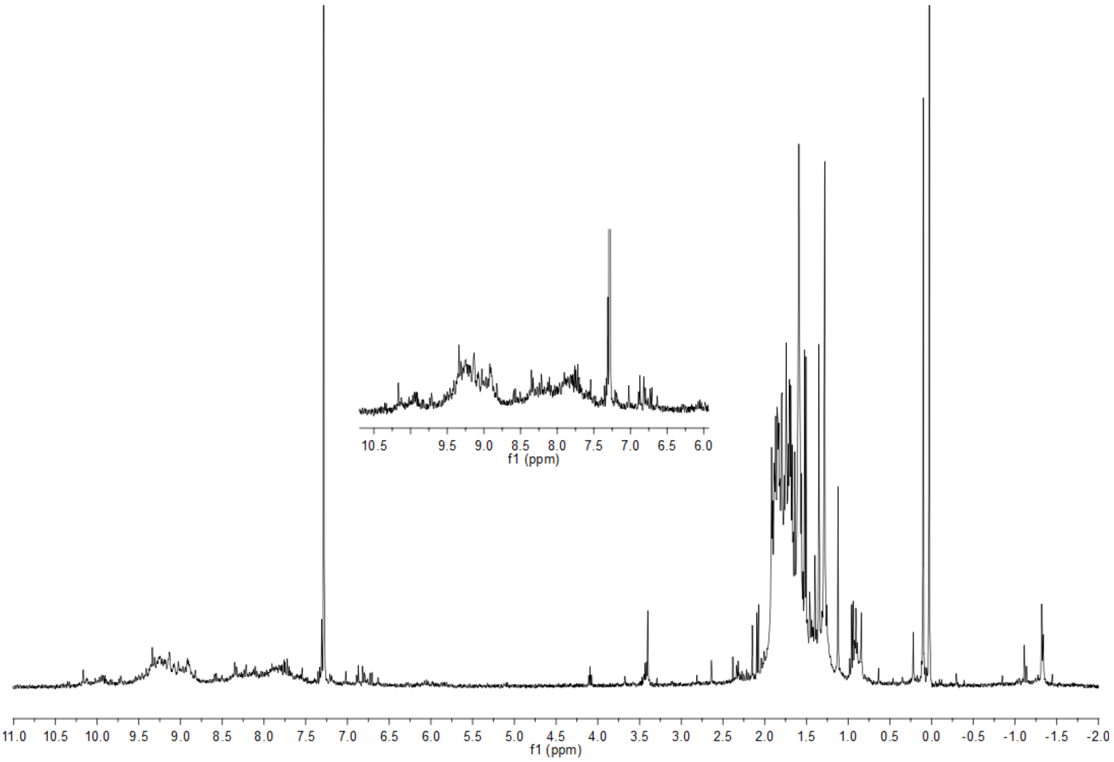

Supplementary Figure 7. A typical ^{1}H NMR from crude solution-phase Scholl reaction mixture involving compound **6** as a starting material.

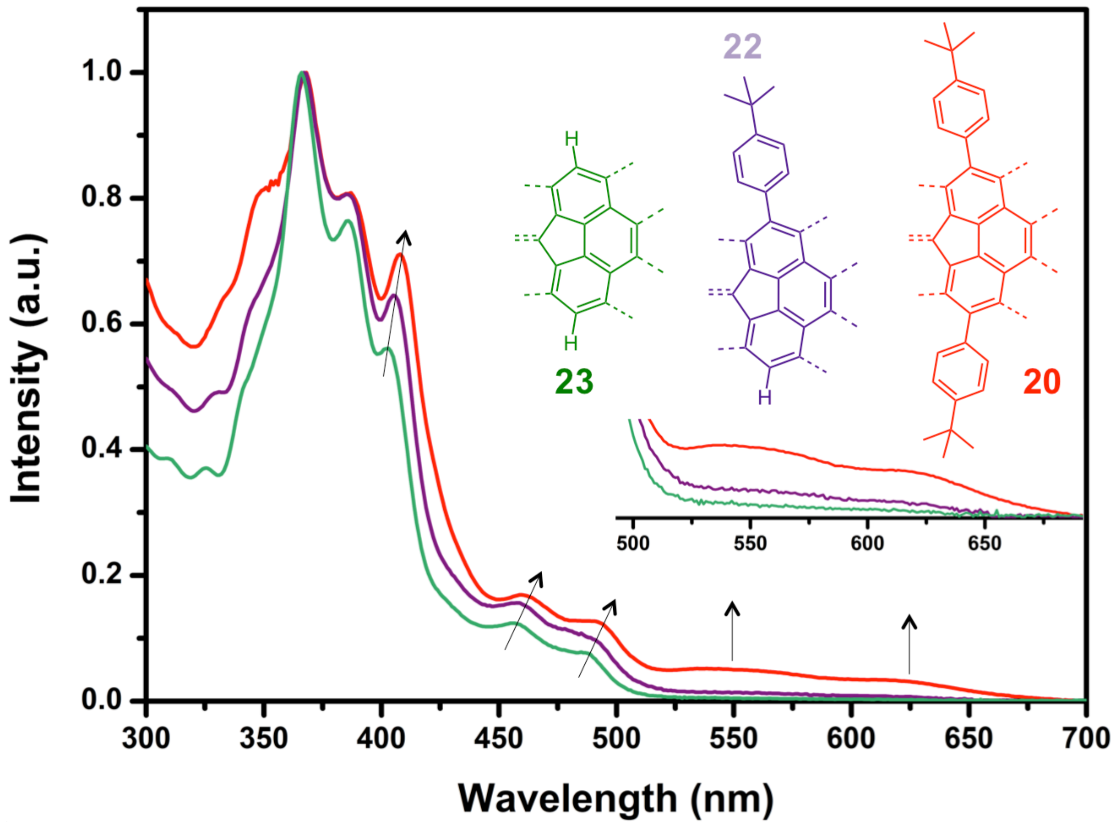

Supplementary Figure 8. The effect of functionalization on the absorption properties of the nanographenes. Partial chemical structures of **20**, **22**, and **23** are shown.

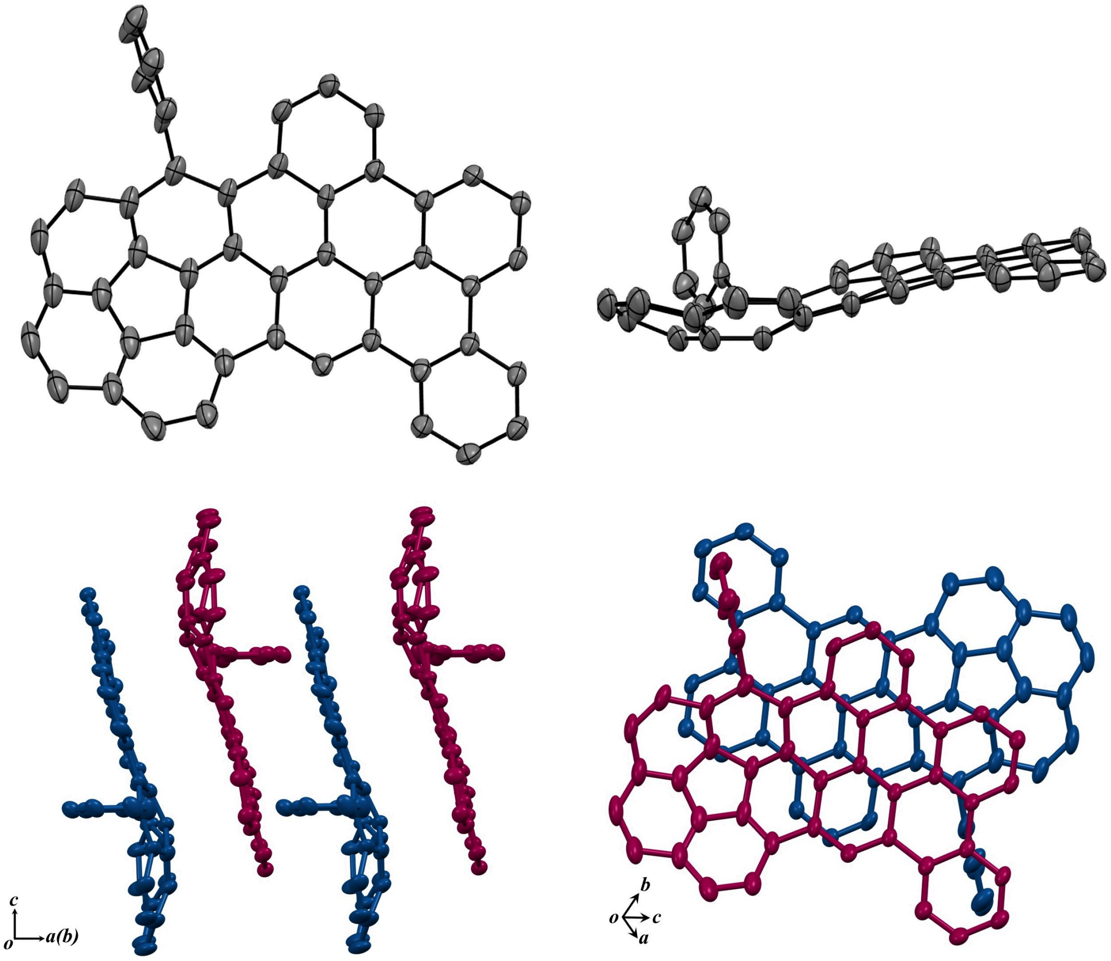

Supplementary Figure 9. X-Ray crystal structure of **24**. Thermal ellipsoids were scaled at 50% probability level. Top view (top left), side view (top right); packing structure (bottom), *tert*-butyl groups and hydrogen atoms were omitted for clarity.

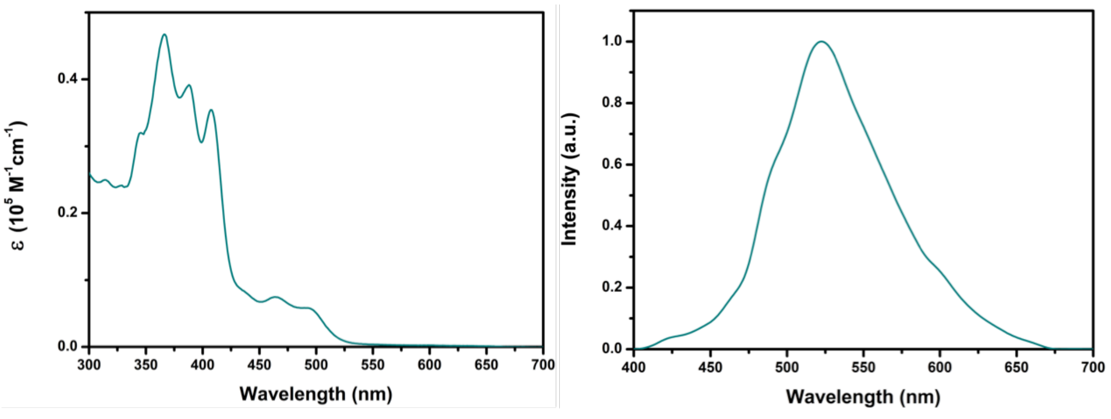

Supplementary Figure 10. UV-vis (dichloromethane; c = 10^{-5} mol L^{-1}) (left) and emission spectra (dichloromethane) (right) of **7**.

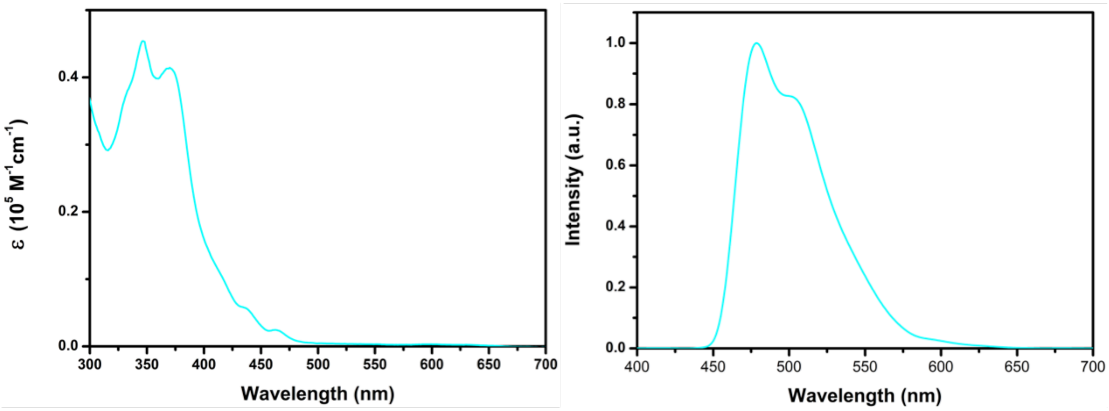

Supplementary Figure 11. UV-vis (dichloromethane; c = 10^{-5} mol L^{-1}) (left) and emission spectra (dichloromethane) (right) of **11**.

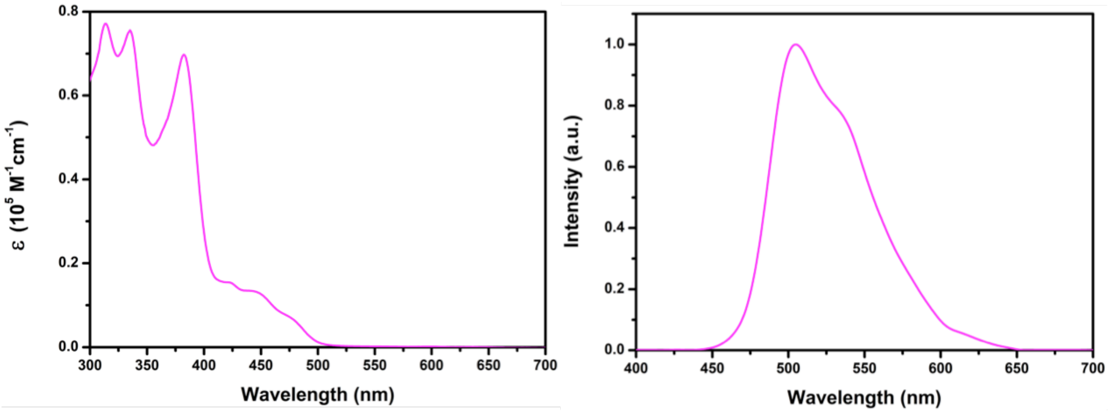

Supplementary Figure 12. UV-vis (dichloromethane; c = 10^{-5} mol L^{-1}) (left) and emission spectra (dichloromethane) (right) of **12**.

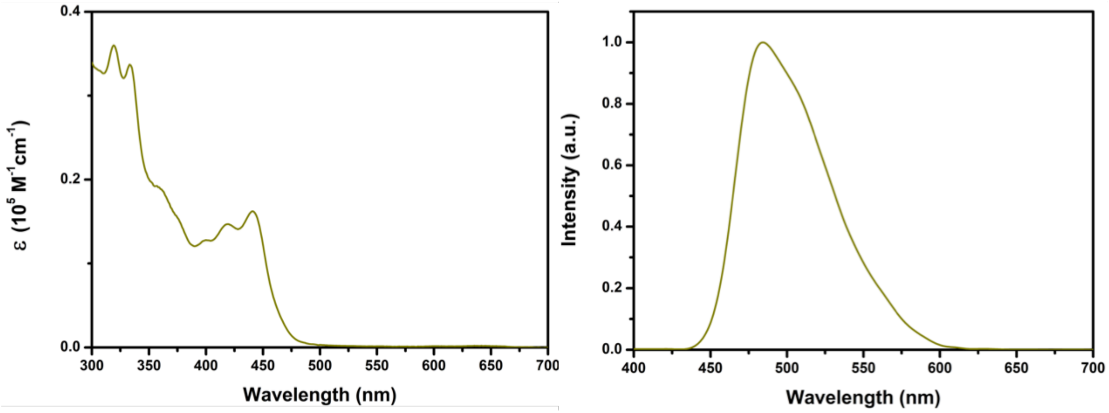

Supplementary Figure 13. UV-vis (dichloromethane; c = 10^{-5} mol L^{-1}) (left) and emission spectra (dichloromethane) (right) of **16**.

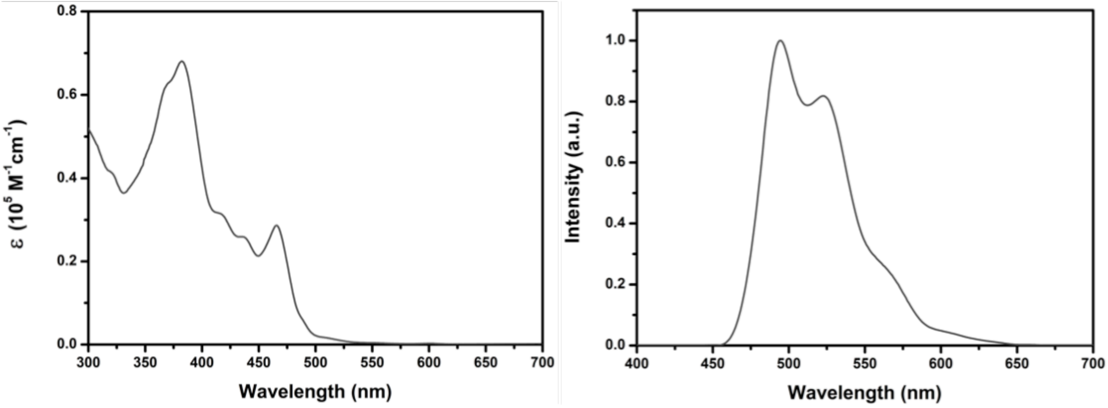

Supplementary Figure 14. UV-vis (dichloromethane; c = 10^{-5} mol L^{-1}) (left) and emission spectra (dichloromethane) (right) of **18**.

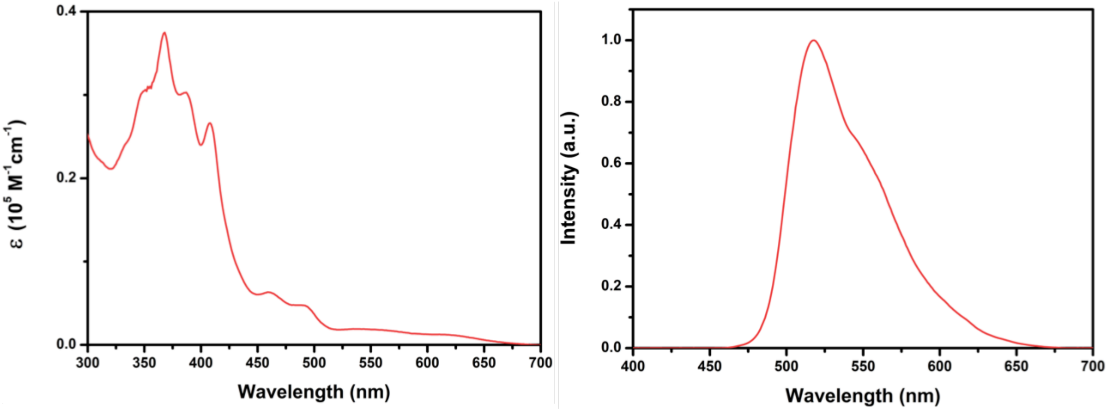

Supplementary Figure 15. UV-vis (dichloromethane; c = 10^{-5} mol L^{-1}) (left) and emission spectra (dichloromethane) (right) of **20**.

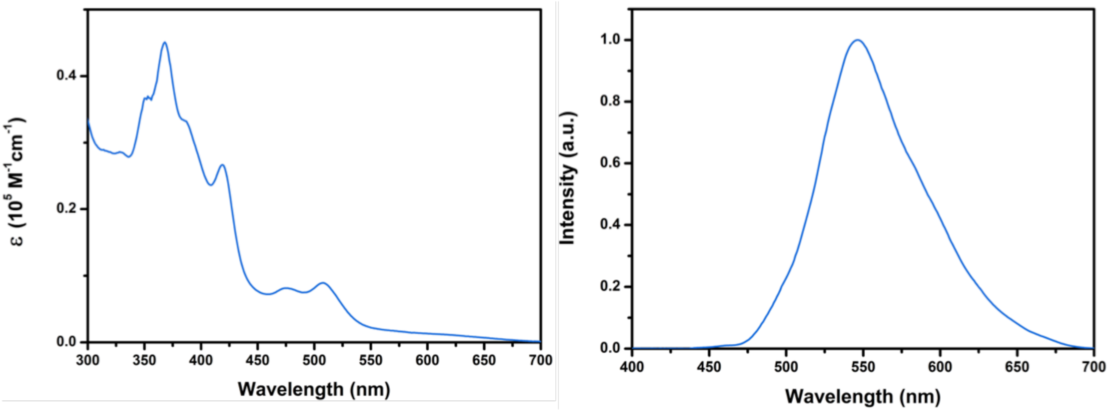

Supplementary Figure 16. UV-vis (dichloromethane; c = 10^{-5} mol L^{-1}) (left) and emission spectra (dichloromethane) (right) of **21**.

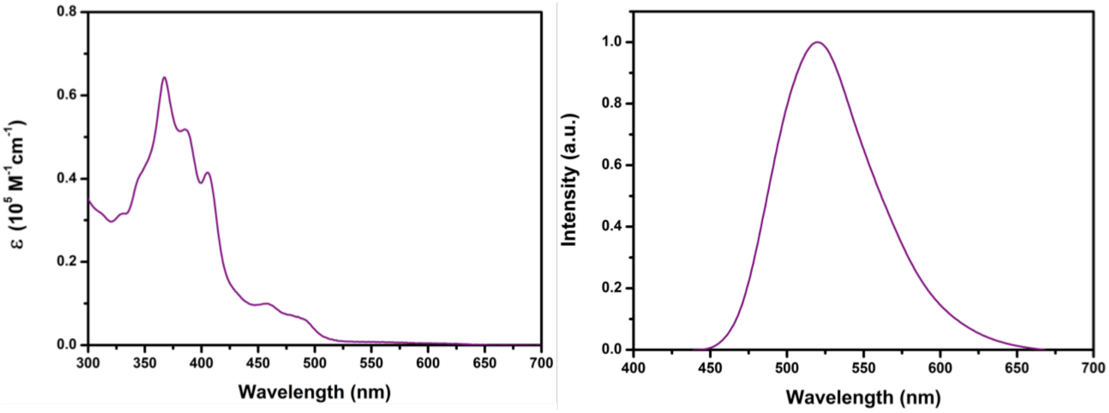

Supplementary Figure 17. UV-vis (dichloromethane; c = 10^{-5} mol L^{-1}) (left) and emission spectra (dichloromethane) (right) of **22**.

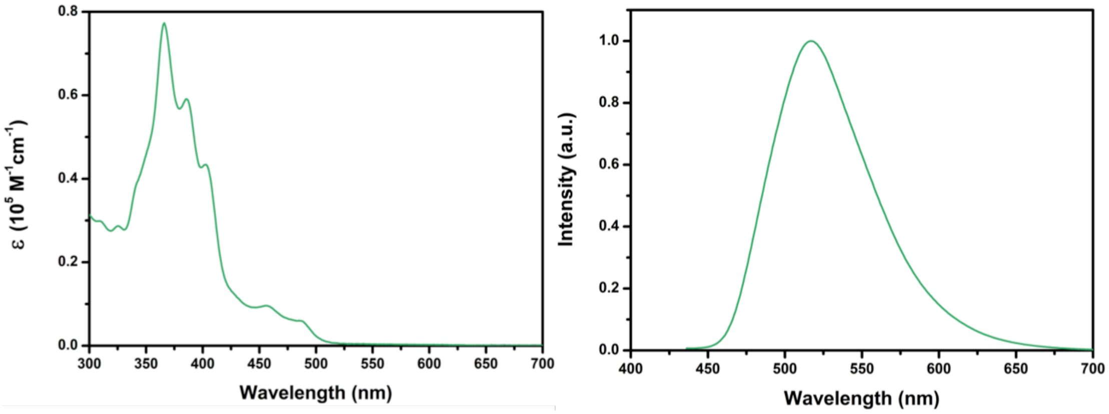

Supplementary Figure 18. UV-vis (dichloromethane; c = 10^{-5} mol L^{-1}) (left) and emission spectra (dichloromethane) (right) of **23**.

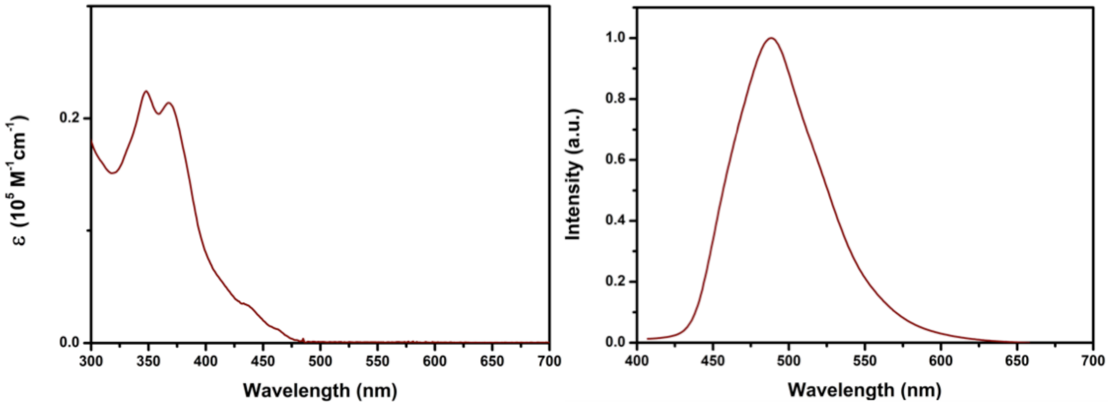

Supplementary Figure 19. UV-vis (dichloromethane; c = 10^{-5} mol L^{-1}) (left) and emission spectra (dichloromethane) (right) of **24**.

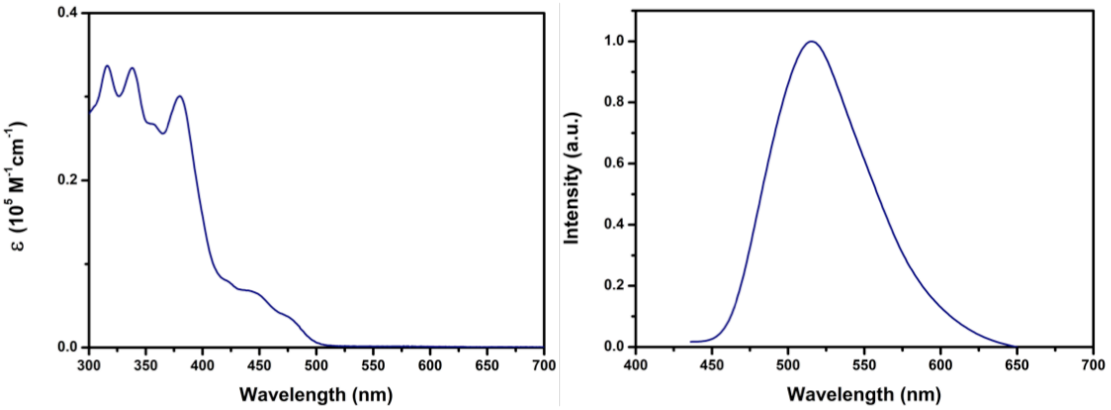

Supplementary Figure 20. UV-vis (dichloromethane; c = 10^{-5} mol L^{-1}) (left) and emission spectra (dichloromethane) (right) of **25**.

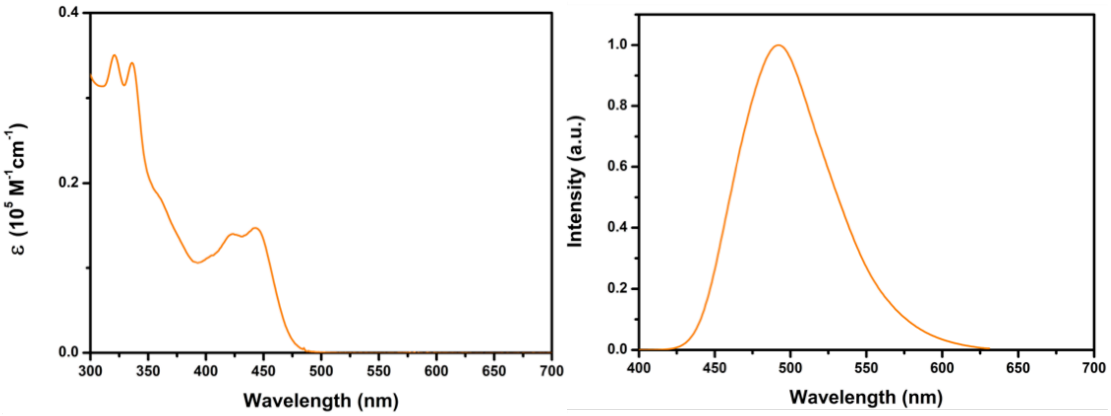

Supplementary Figure 21. UV-vis (dichloromethane; c = 10^{-5} mol L^{-1}) (left) and emission spectra (dichloromethane) (right) of **26**.

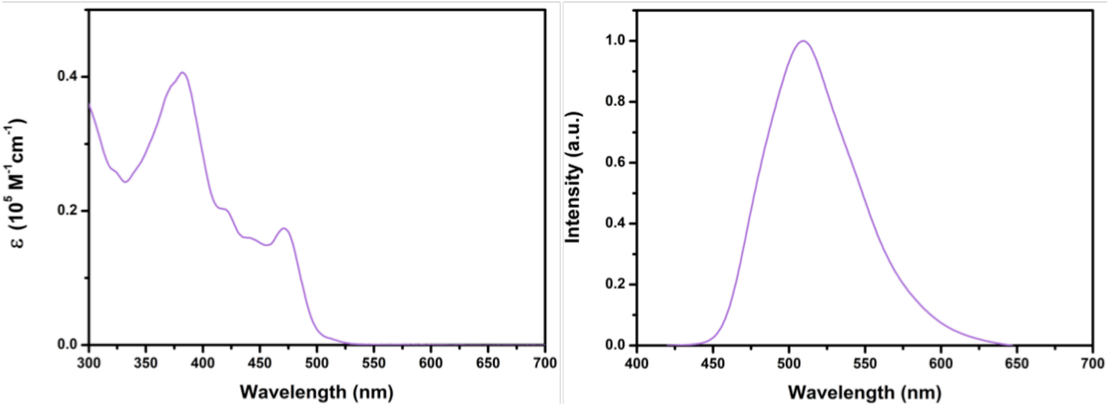

Supplementary Figure 22. UV-vis (dichloromethane; c = 10^{-5} mol L^{-1}) (left) and emission spectra (dichloromethane) (right) of **27**.

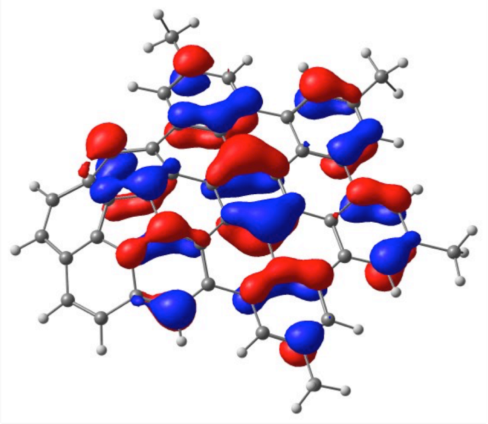

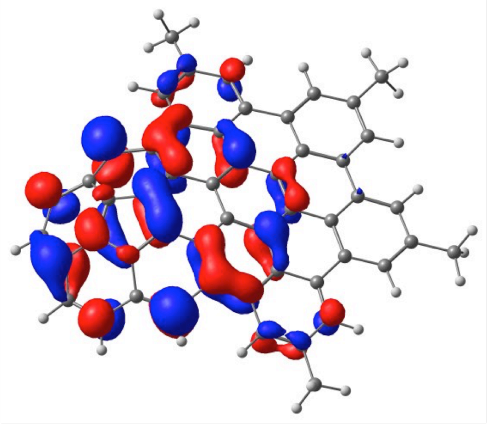

7’ **7’**

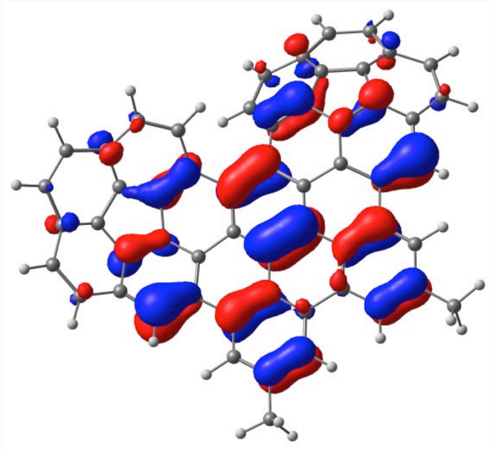

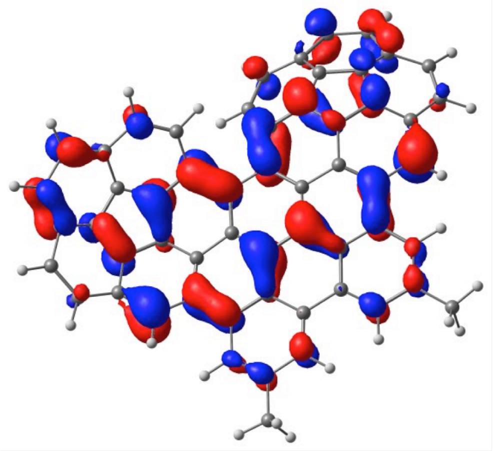

18’ **18’**

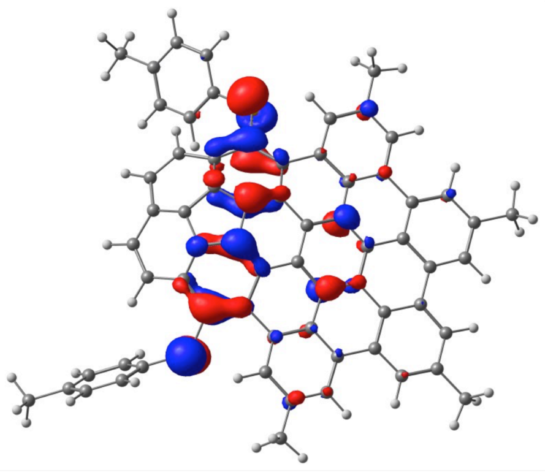

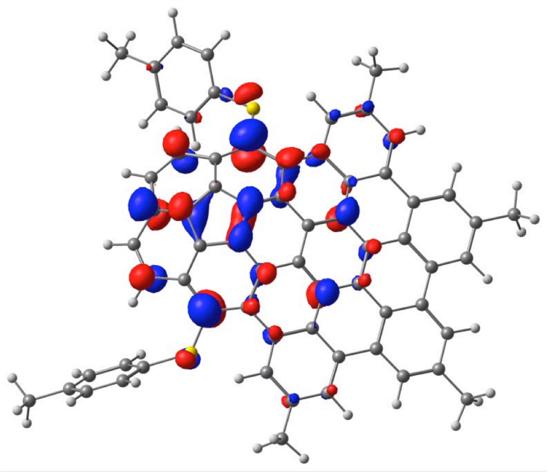

21’ **21’**

Supplementary Figure 23. Computed LUMO (right) and HOMO (left) for **7’** (top), **18’** (middle), and **21’** (bottom).

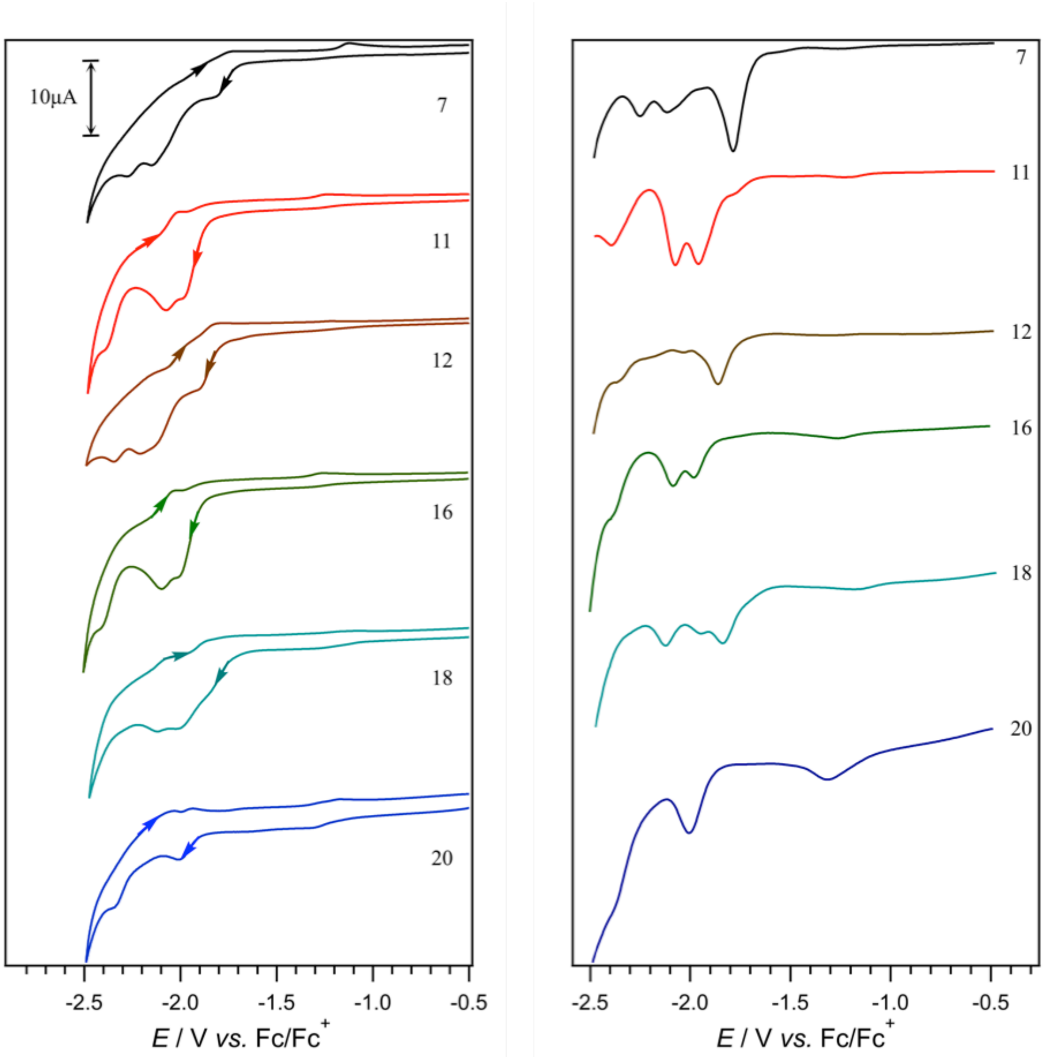

Supplementary Figure 24. Cyclic voltammograms (left panel) and square-wave voltammograms (right panel) were measured at room temperature in dry, degassed dichloromethane with *n*-Bu_4NPF_6 (0.1 M) as the supporting electrolyte at a 1 mm diameter planar glassy carbon electrode under an argon atmosphere. CVs were measured at a scan rate of 0.1 V s^{-1} and SWVs were recorded with a pulse period (τ) = 25 Hz, a potential step = 2 mV and a pulse amplitude = 20 mV.

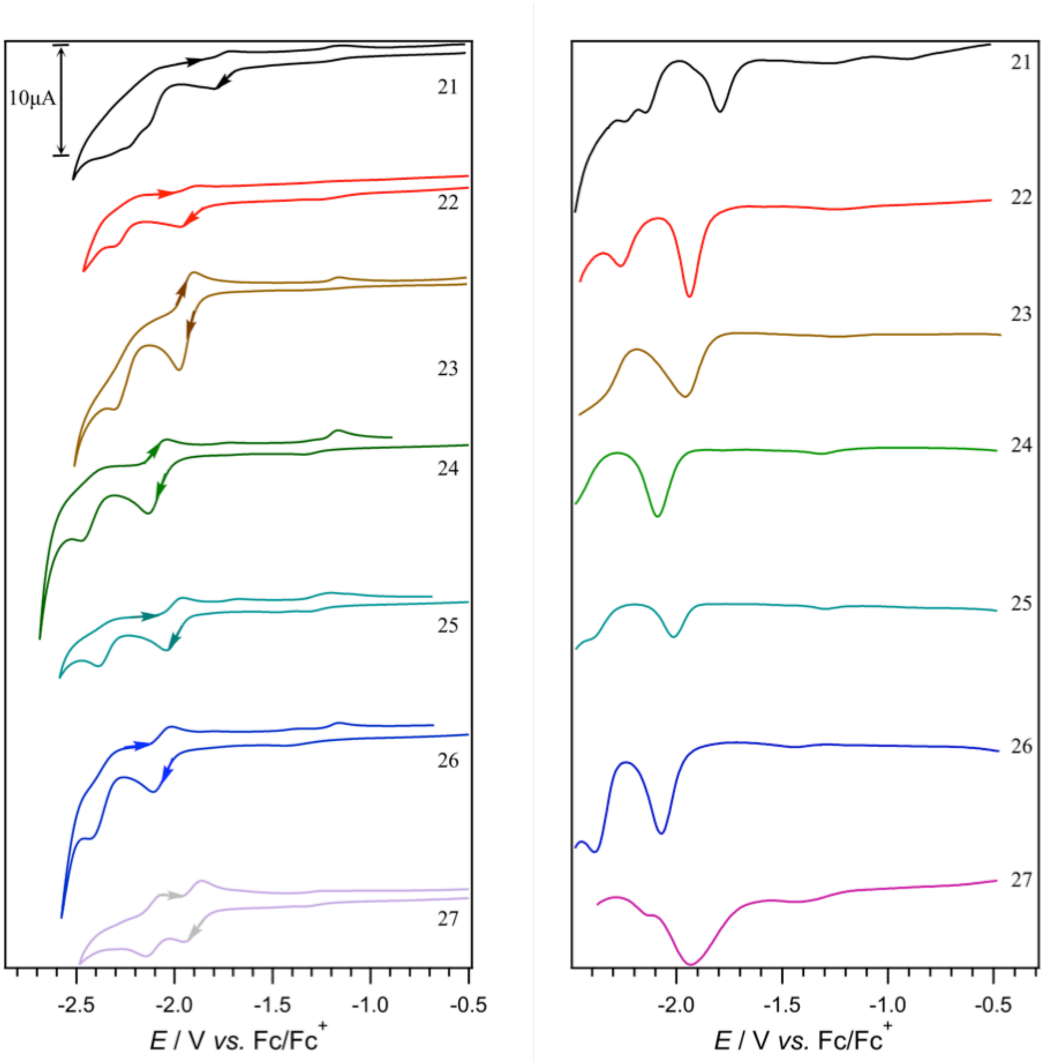

Supplementary Figure 25. Cyclic voltammograms (left panel) and square-wave voltammograms (right panel) were measured at room temperature in dry, degassed dichloromethane with *n*-Bu_4NPF_6 (0.1 M) as the supporting electrolyte at a 1 mm diameter planar glassy carbon electrode under an argon atmosphere. CVs were measured at a scan rate of 0.1 V s^{-1} and SWVs were recorded with a pulse period (τ) = 25 Hz, a potential step = 2 mV and a pulse amplitude = 20 mV.

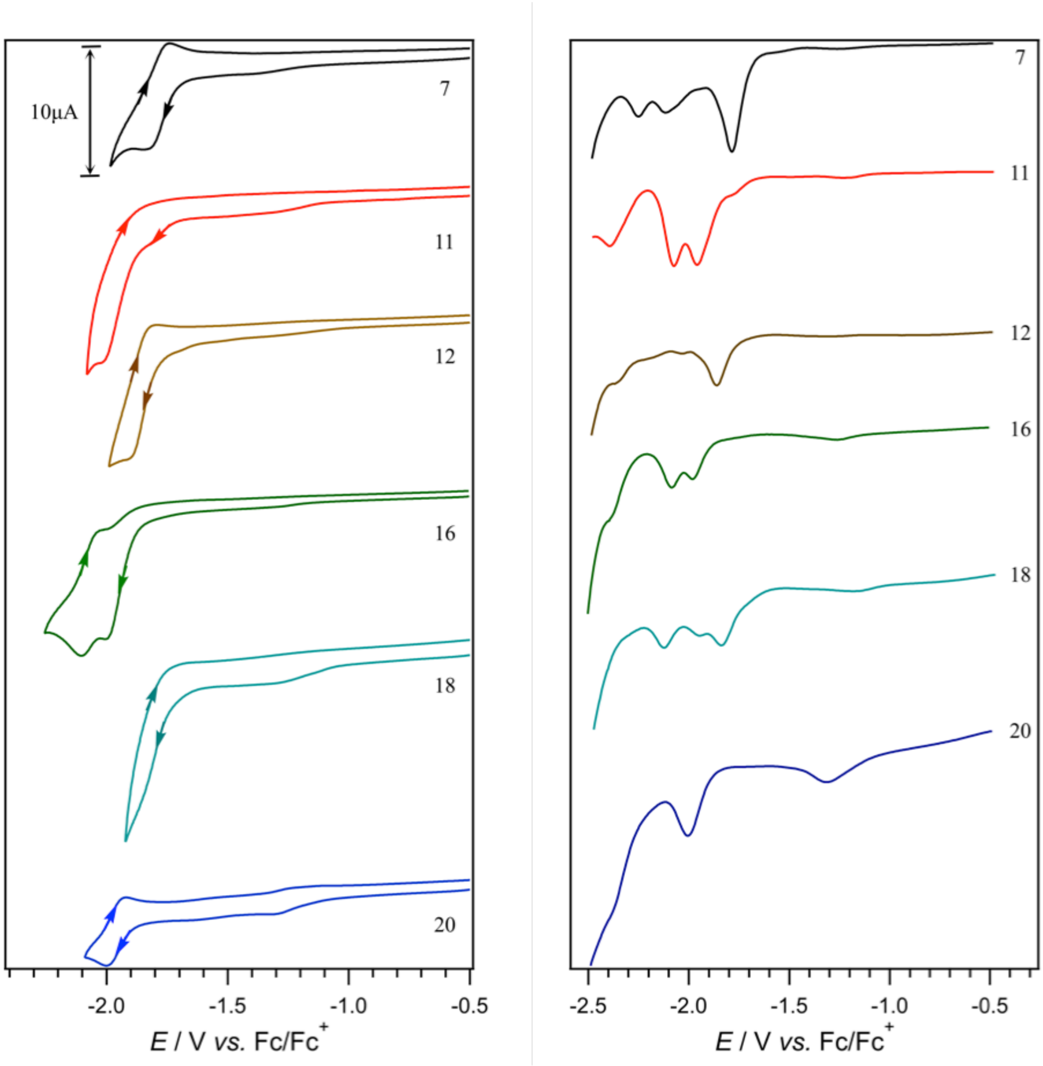

Supplementary Figure 26. Cyclic voltammograms (left panel) and square-wave voltammograms (right panel) were measured at room temperature in dry, degassed dichloromethane with n-Bu_4NPF_6 (0.1 M) as the supporting electrolyte at a 1 mm diameter planar glassy carbon electrode under an argon atmosphere. CVs were measured at a scan rate of 0.1 V s^{-1} and SWVs were recorded with a pulse period (τ) = 25 Hz, a potential step = 2 mV and a pulse amplitude = 20 mV.

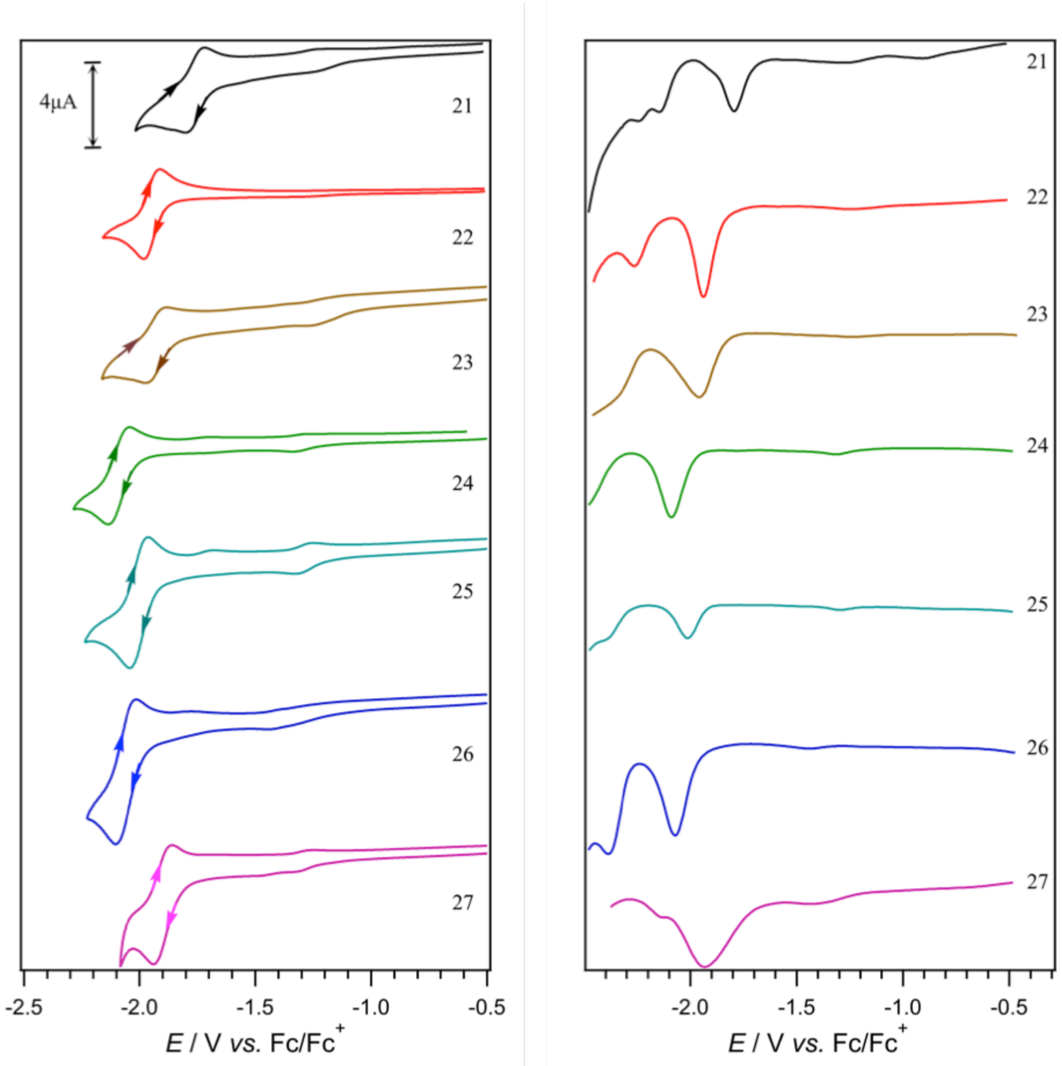

Supplementary Figure 27. Cyclic voltammograms (left panel) and square-wave voltammograms (right panel) were measured at room temperature in dry, degassed dichloromethane with *n*-Bu_4NPF_6 (0.1 M) as the supporting electrolyte at a 1 mm diameter planar glassy carbon electrode under an argon atmosphere. CVs were measured at a scan rate of 0.1 V s^{-1} and SWVs were recorded with a pulse period (τ) = 25 Hz, a potential step = 2 mV and a pulse amplitude = 20 mV.

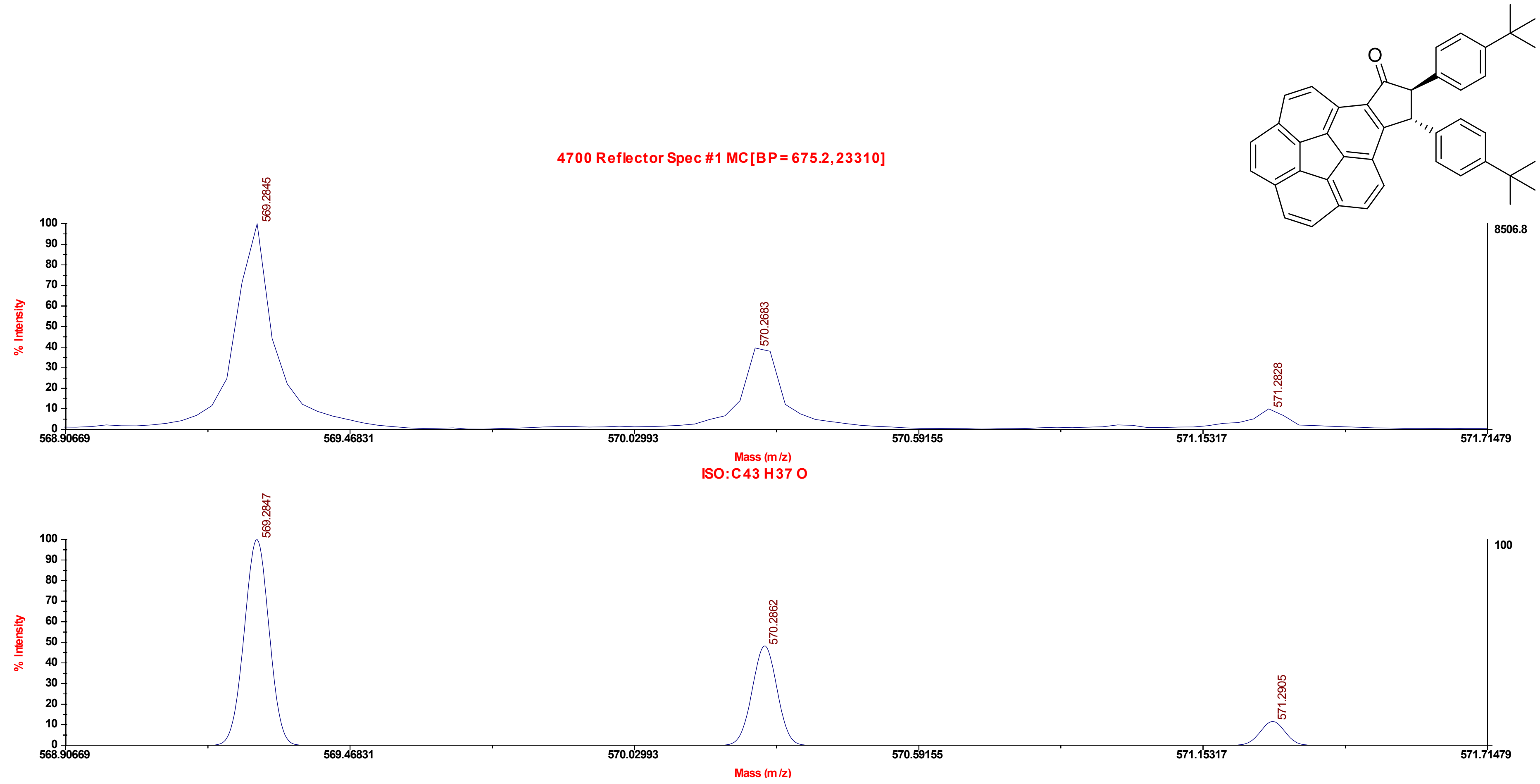

Index	Input m/z	Calc. Mass	Error (mDa)	Error (ppm)	DBE	Formula	Isotope Match Score
1	569.28455	569.28389	0.6575	1.1549	25.50	C43 H36 O	0.196840

Supplementary Figure 28. MALDI/TOF (HRMS) mass spectrum of **3**.

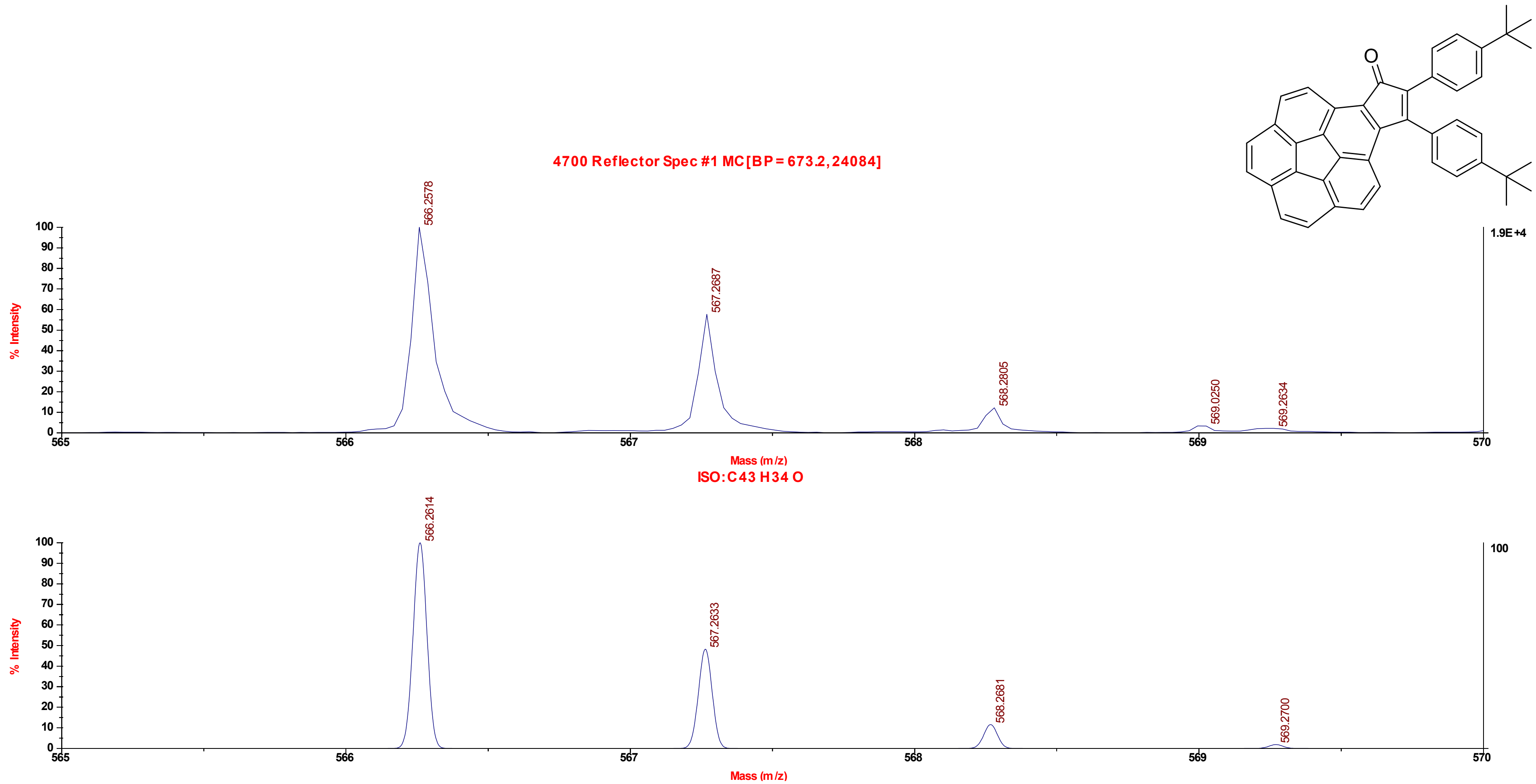

Index	Input m/z	Calc. Mass	Error (mDa)	Error (ppm)	DBE	Formula	Isotope Match Score
1	566.25781	566.26042	-2.6074	-4.6046	27.00	C43 H34 O	0.994338

Supplementary Figure 29. MALDI/TOF (HRMS) mass spectrum of **4**.

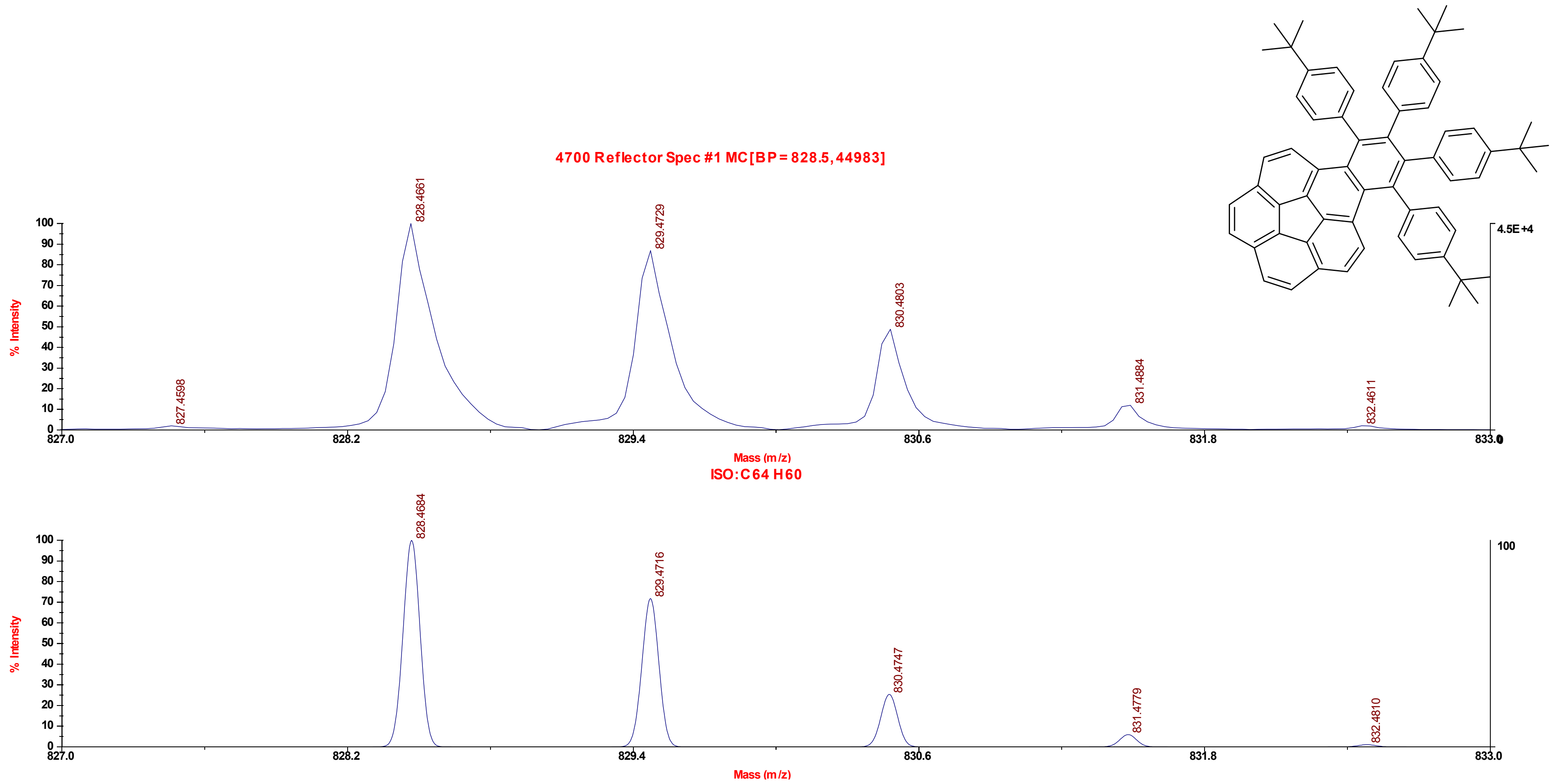

Index	Input m/z	Calc. Mass	Error (mDa)	Error (ppm)	DBE	Formula	Isotope Match Score
1	828.46606	828.46895	-2.8938	-3.4930	35.00	C64 H60	0.989619

Supplementary Figure 30. MALDI/TOF (HRMS) mass spectrum of **6**.

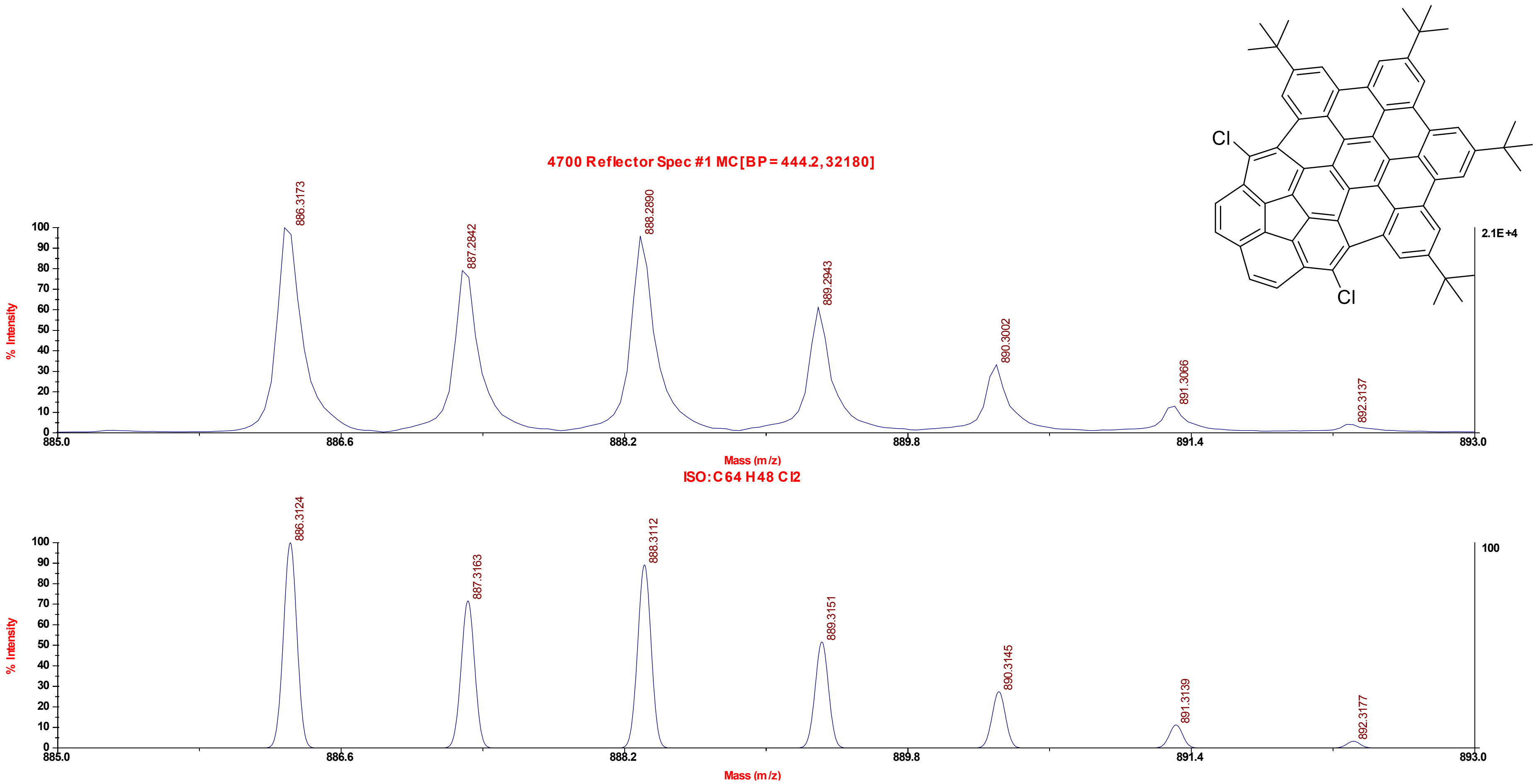

Index	Input m/z	Calc. Mass	Error (mDa)	Error (ppm)	DBE	Formula	Isotope Match Score
1	886.31726	886.31276	4.5012	5.0785	40.00	C64 H48 Cl2	0.370591

Supplementary Figure 31. MALDI/TOF (HRMS) mass spectrum of **7**.

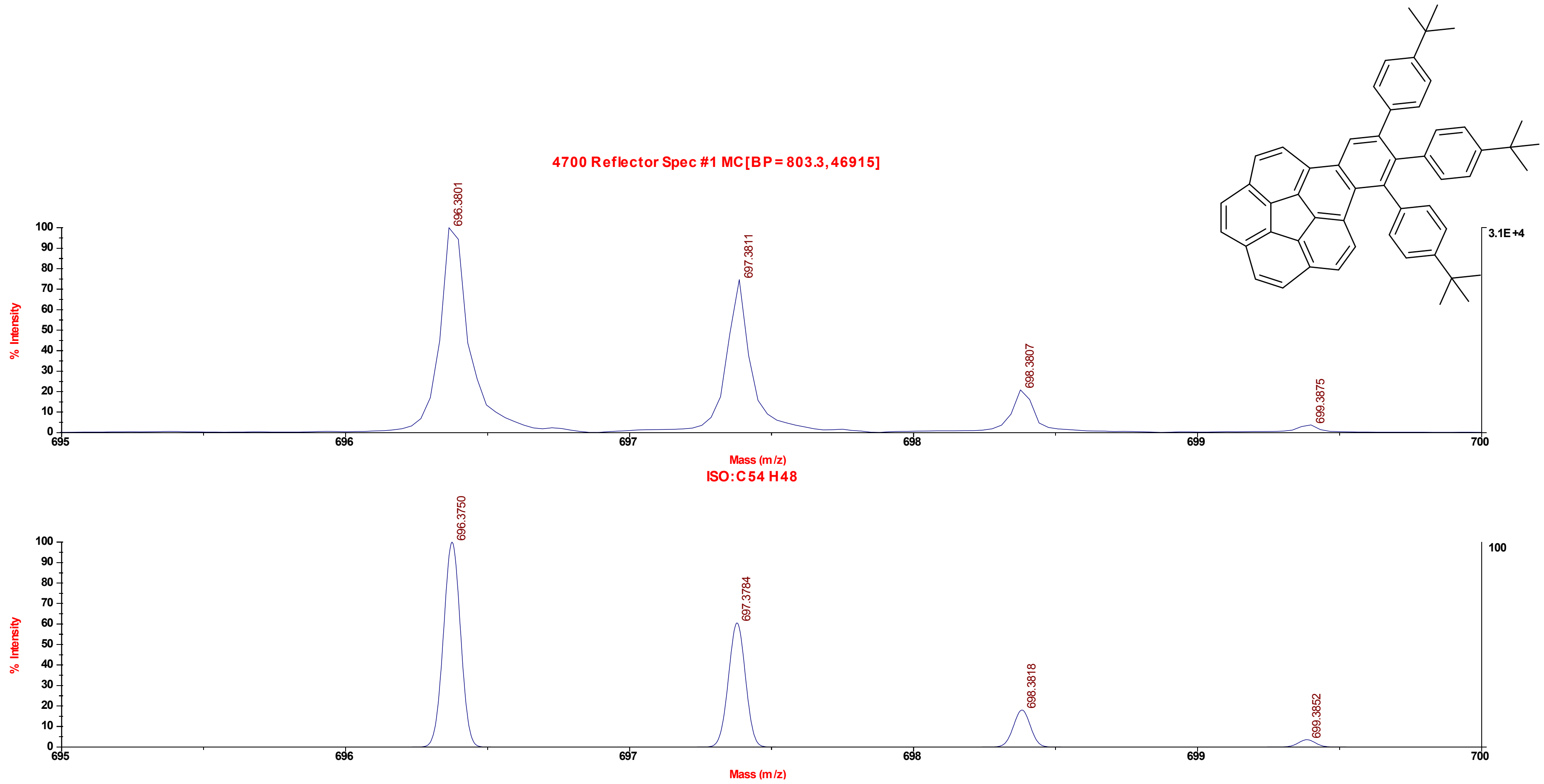

Index	Input m/z	Calc. Mass	Error (mDa)	Error (ppm)	DBE	Formula	Isotope Match Score
1	696.38013	696.37505	5.0767	7.2901	31.00	C54 H48	0.999761

Supplementary Figure 32. MALDI/TOF (HRMS) mass spectrum of **9**.

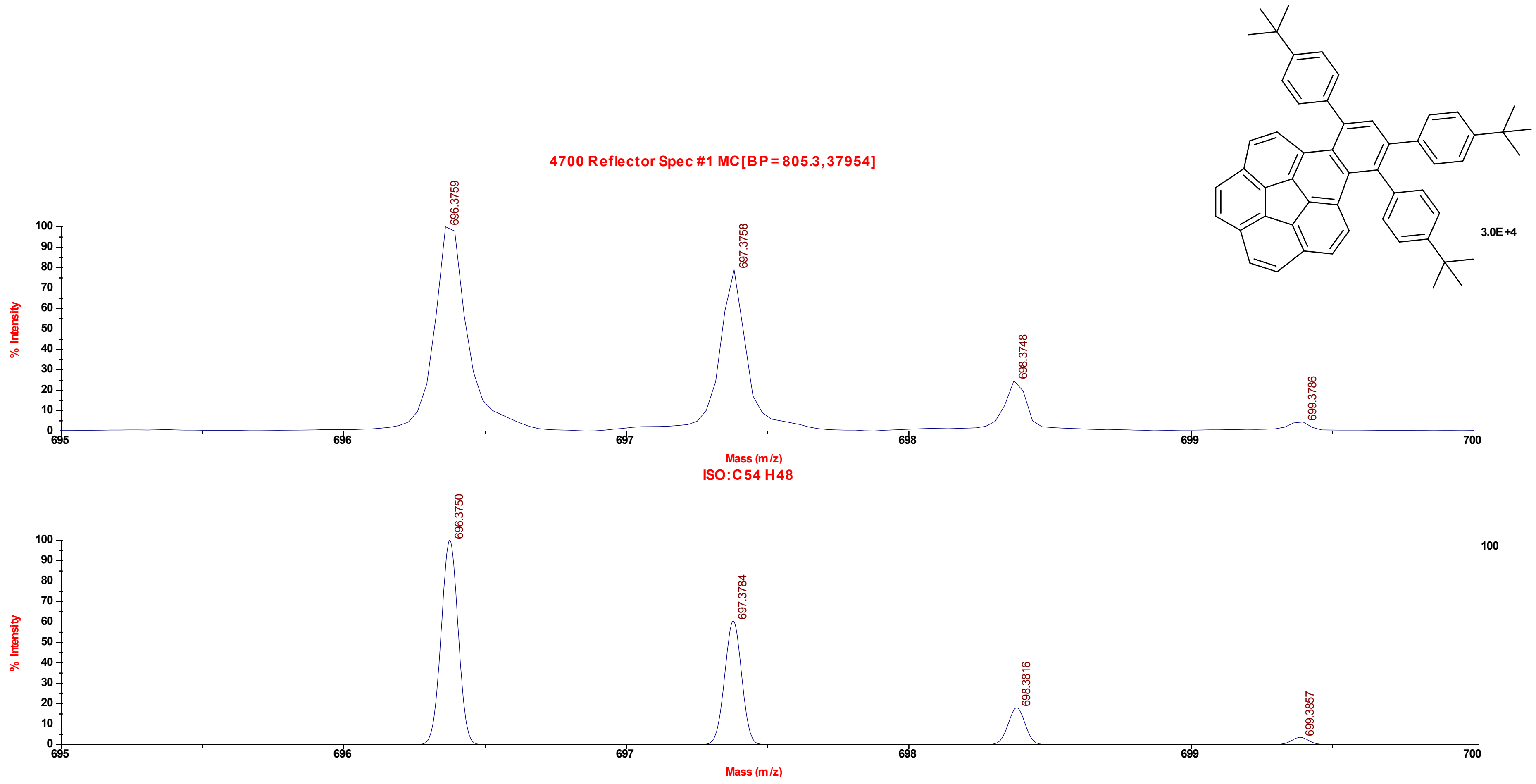

Index	Input m/z	Calc. Mass	Error (mDa)	Error (ppm)	DBE	Formula	Isotope Match Score
1	696.37585	696.37505	0.7967	1.1440	31.00	C54 H48	0.998258

Supplementary Figure 33. MALDI/TOF (HRMS) mass spectrum of **10**.

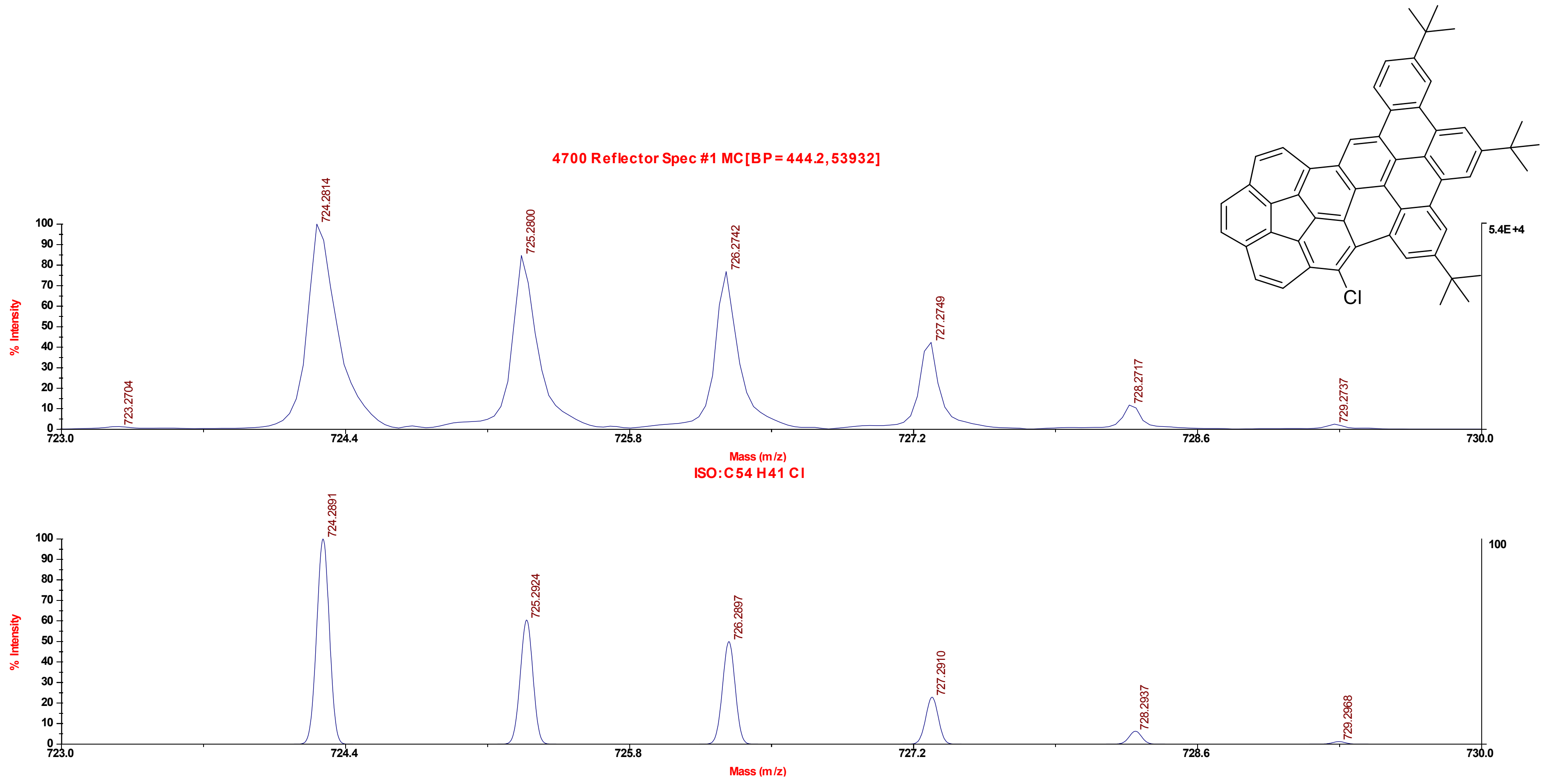

Index	Input m/z	Calc. Mass	Error (mDa)	Error (ppm)	DBE	Formula	Isotope Match Score
1	724.28137	724.28913	-7.7608	-10.7151	34.00	C54 H41 Cl	0.990860

Supplementary Figure 34. MALDI/TOF (HRMS) mass spectrum of **11**.

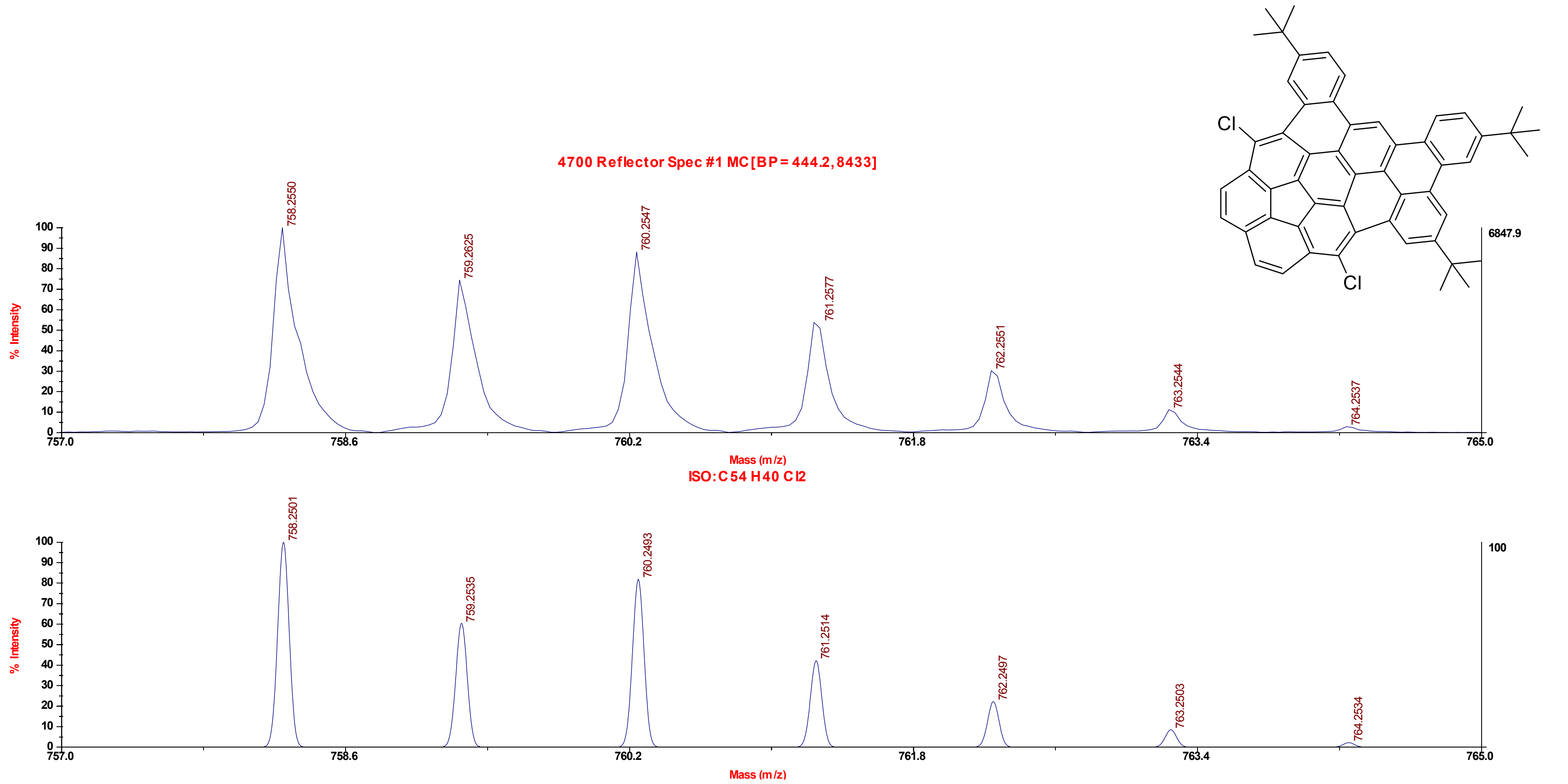

Index	Input m/z	Calc. Mass	Error (mDa)	Error (ppm)	DBE	Formula	Isotope Match Score
1	758.25500	758.25016	4.8415	6.3851	34.00	C54 H40 Cl2	0.993330

Supplementary Figure 35. MALDI/TOF (HRMS) mass spectrum of **12**.

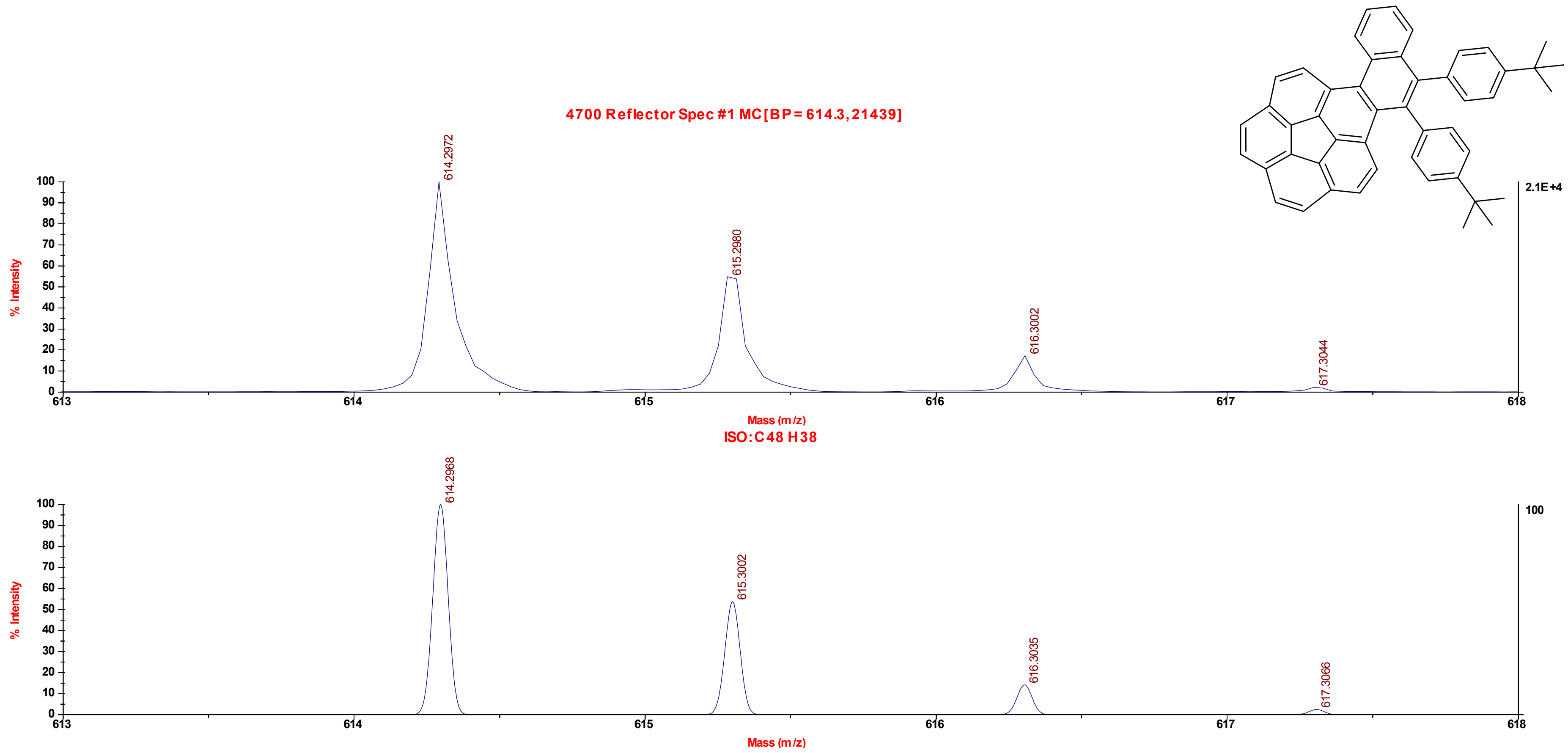

Index	Input m/z	Calc. Mass	Error (mDa)	Error (ppm)	DBE	Formula	Isotope Match Score
1	614.29718	614.29680	0.3771	0.6138	30.00	C48 H38	0.810701

Supplementary Figure 36. MALDI/TOF (HRMS) mass spectrum of **15**.

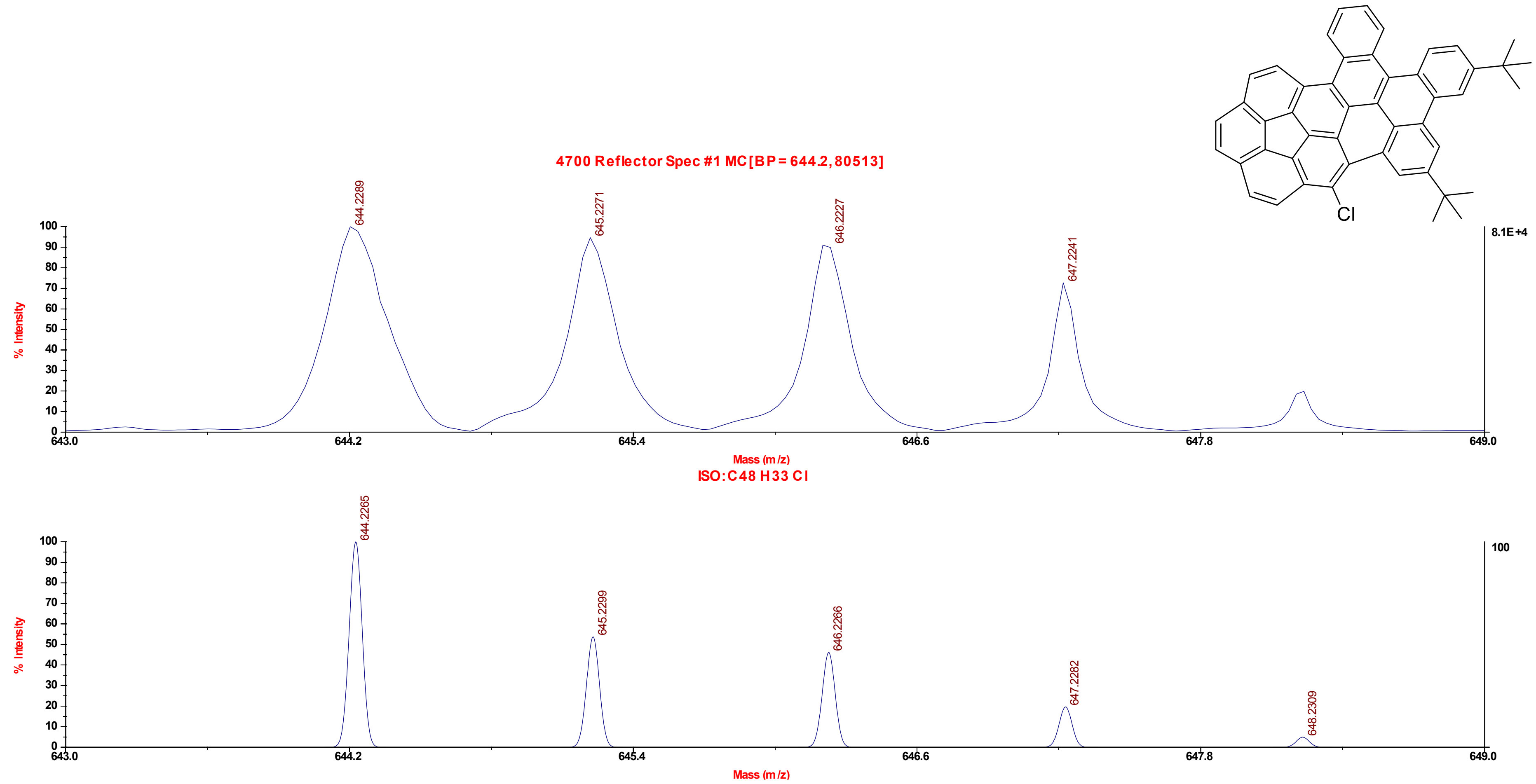

Index	Input m/z	Calc. Mass	Error (mDa)	Error (ppm)	DBE	Formula	Isotope Match Score
1	644.22890	644.22653	2.3695	3.6781	32.00	C48 H33 Cl	0.964820

Supplementary Figure 37. MALDI/TOF (HRMS) mass spectrum of **16**.

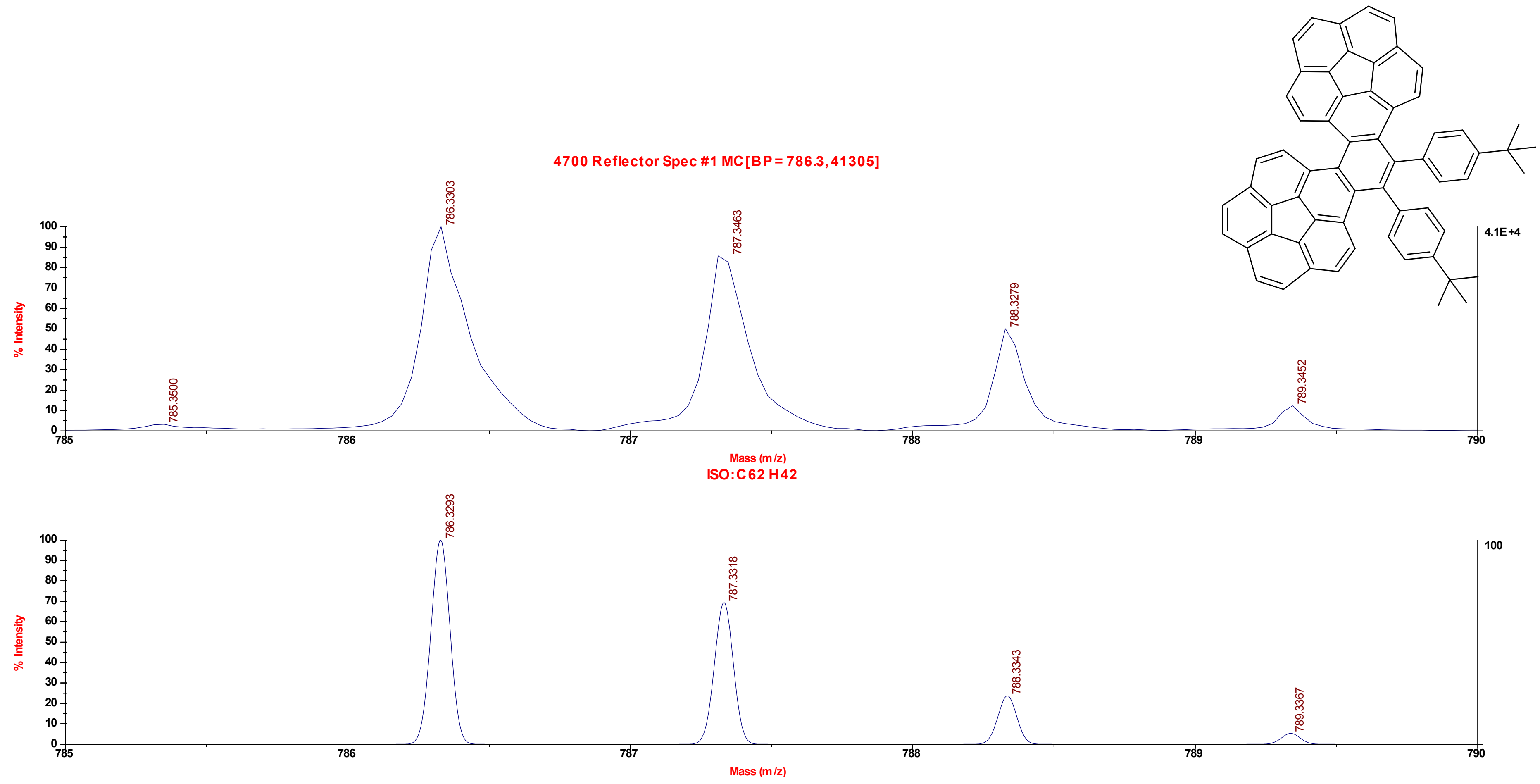

Index	Input m/z	Calc. Mass	Error (mDa)	Error (ppm)	DBE	Formula	Isotope Match Score
1	786.33026	786.32810	2.1569	2.7430	42.00	C62 H42	0.990462

Supplementary Figure 38. MALDI/TOF (HRMS) mass spectrum of **17**.

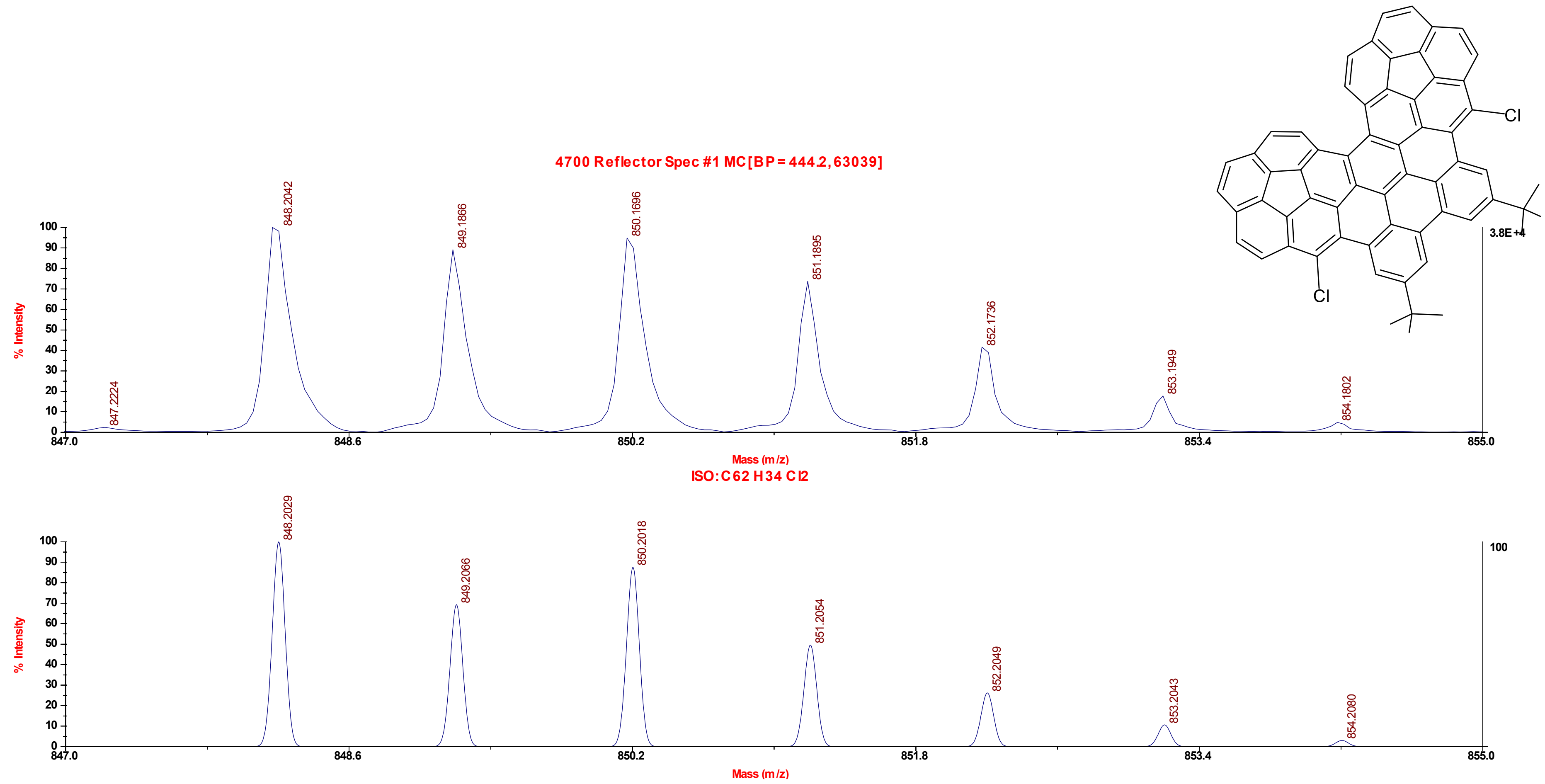

Index	Input m/z	Calc. Mass	Error (mDa)	Error (ppm)	DBE	Formula	Isotope Match Score
1	848.20416	848.20321	0.9518	1.1221	45.00	C62 H34 Cl2	0.578994

Supplementary Figure 39. MALDI/TOF (HRMS) mass spectrum of **18**.

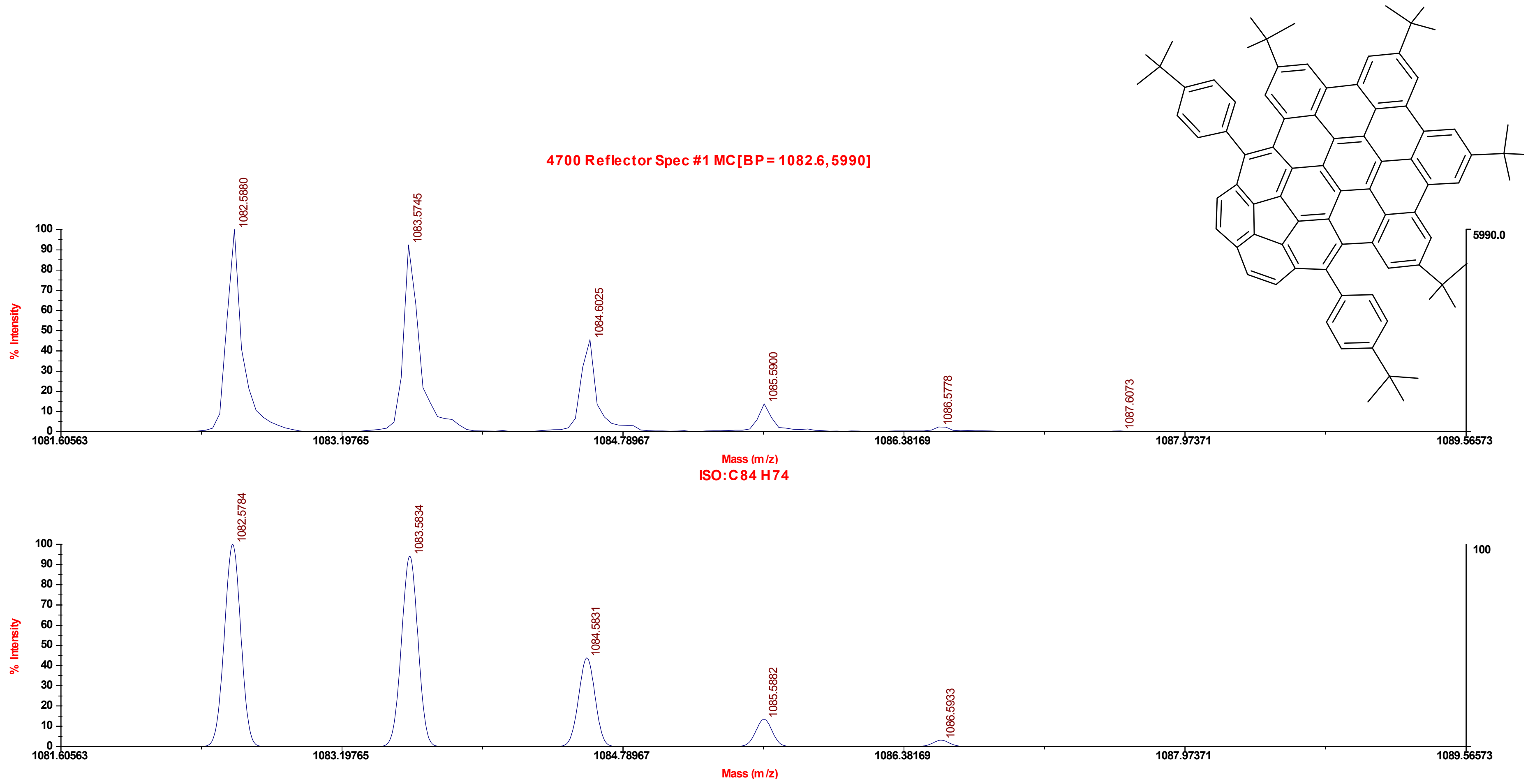

Index	Input m/z	Calc. Mass	Error (mDa)	Error (ppm)	DBE	Formula	Isotope Match Score
1	1082.58801	1082.57850	9.5056	8.7805	48.00	C84 H74	0.999271

Supplementary Figure 40. MALDI/TOF (HRMS) mass spectrum of **20**.

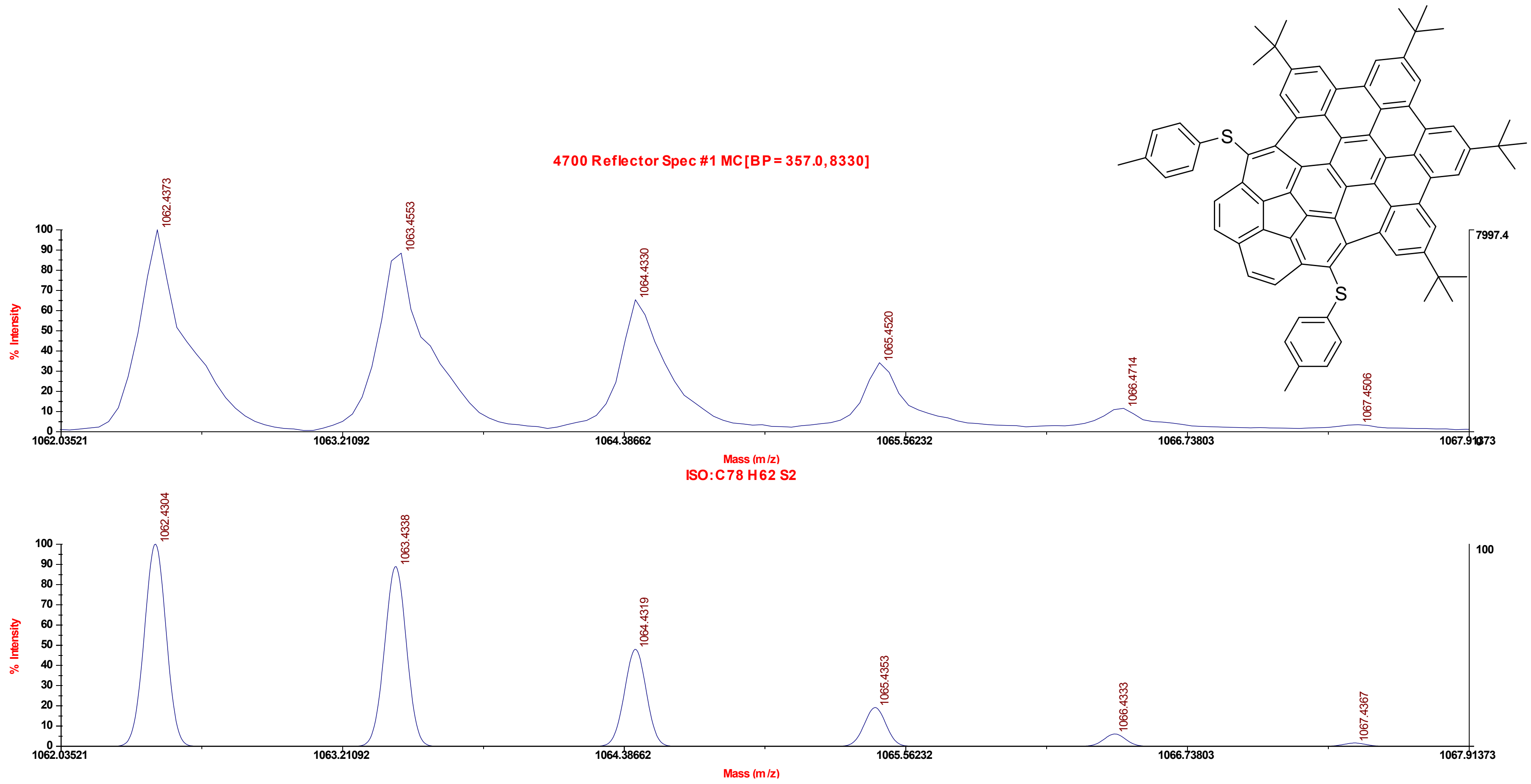

Index	Input m/z	Calc. Mass	Error (mDa)	Error (ppm)	DBE	Formula	Isotope Match Score
1	1062.43726	1062.42875	8.5125	8.0122	48.00	C78 H62 S2	0.693458

Supplementary Figure 41. MALDI/TOF (HRMS) mass spectrum of **21**.

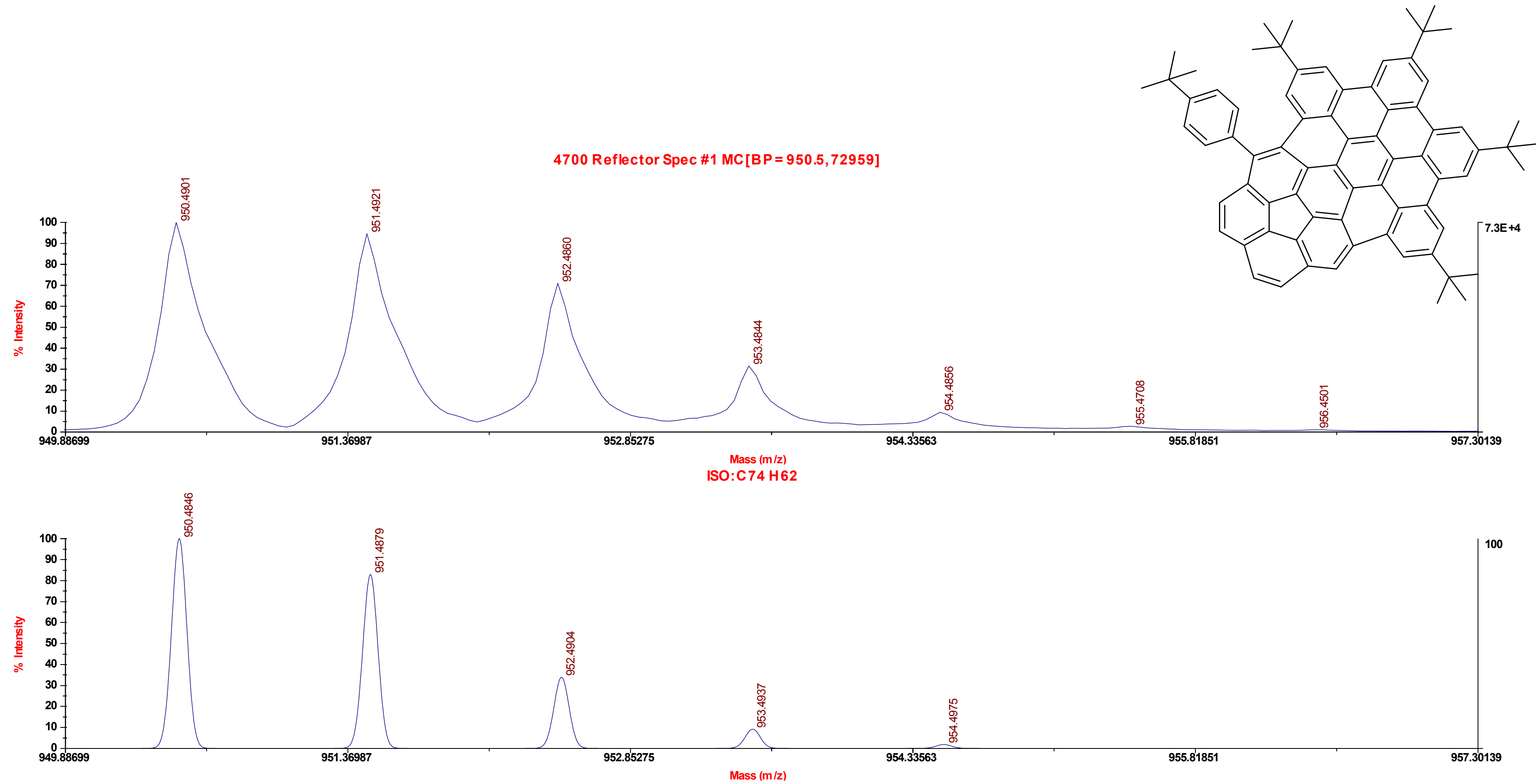

Index	Input m/z	Calc. Mass	Error (mDa)	Error (ppm)	DBE	Formula	Isotope Match Score
1	950.49005	950.48460	5.4461	5.7298	44.00	C74 H62	0.976103

Supplementary Figure 42. MALDI/TOF (HRMS) mass spectrum of **22**.

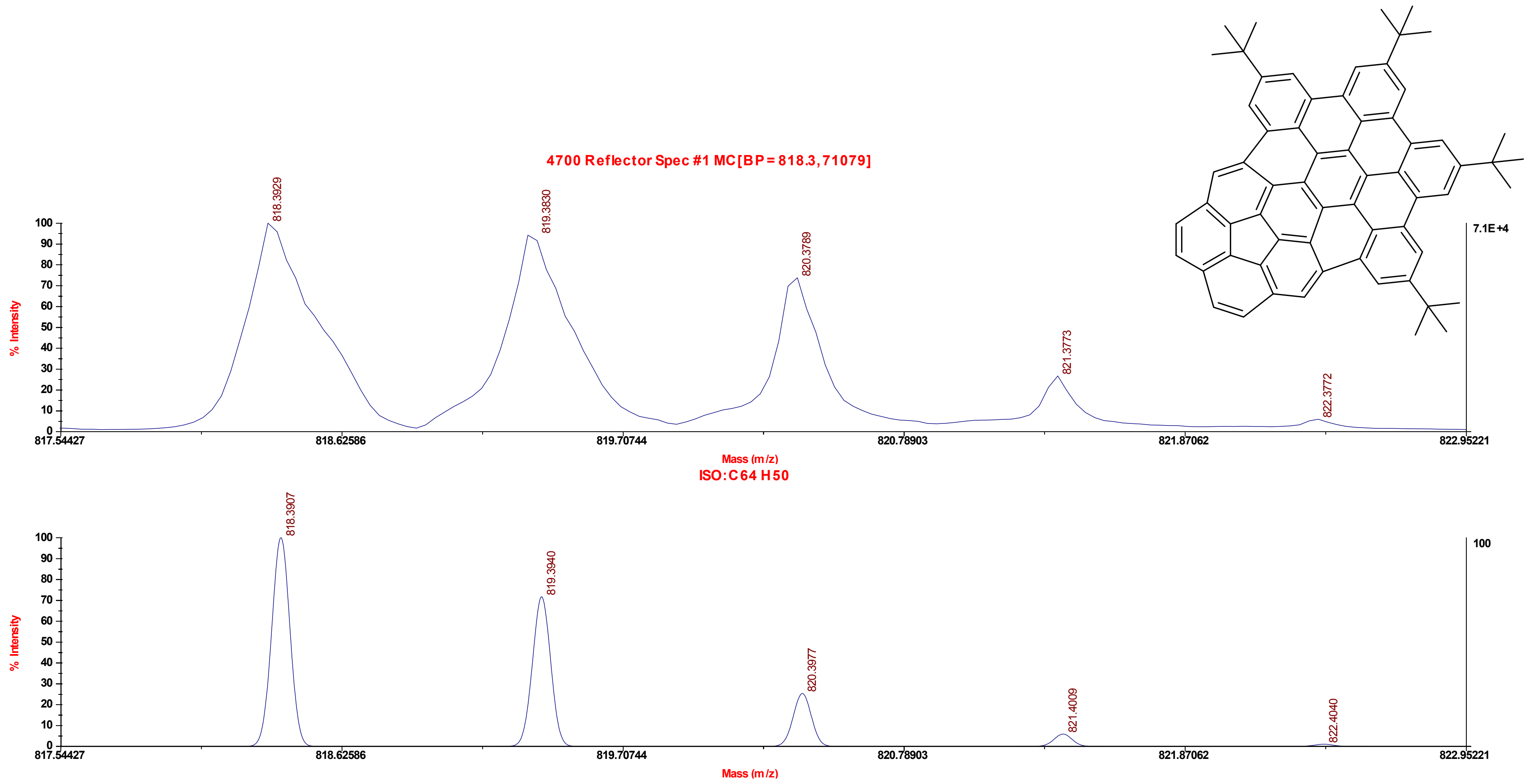

Index	Input m/z	Calc. Mass	Error (mDa)	Error (ppm)	DBE	Formula	Isotope Match Score
1	818.39288	818.39070	2.1766	2.6596	40.00	C64 H50	0.826350

Supplementary Figure 43. MALDI/TOF (HRMS) mass spectrum of **23**.

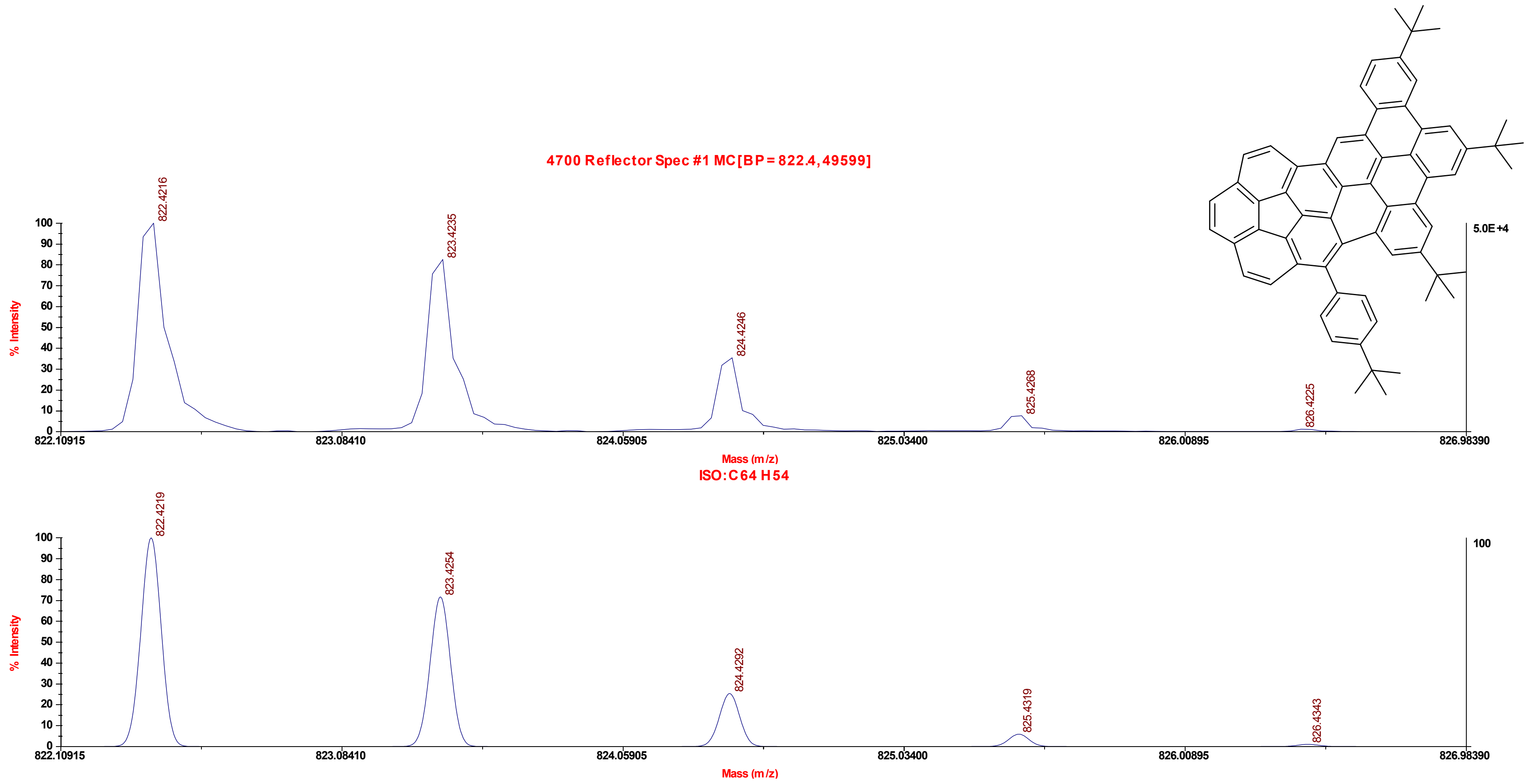

Index	Input m/z	Calc. Mass	Error (mDa)	Error (ppm)	DBE	Formula	Isotope Match Score
1	822.42157	822.42200	-0.4336	-0.5272	38.00	C64 H54	0.997772

Supplementary Figure 44. MALDI/TOF (HRMS) mass spectrum of **24**.

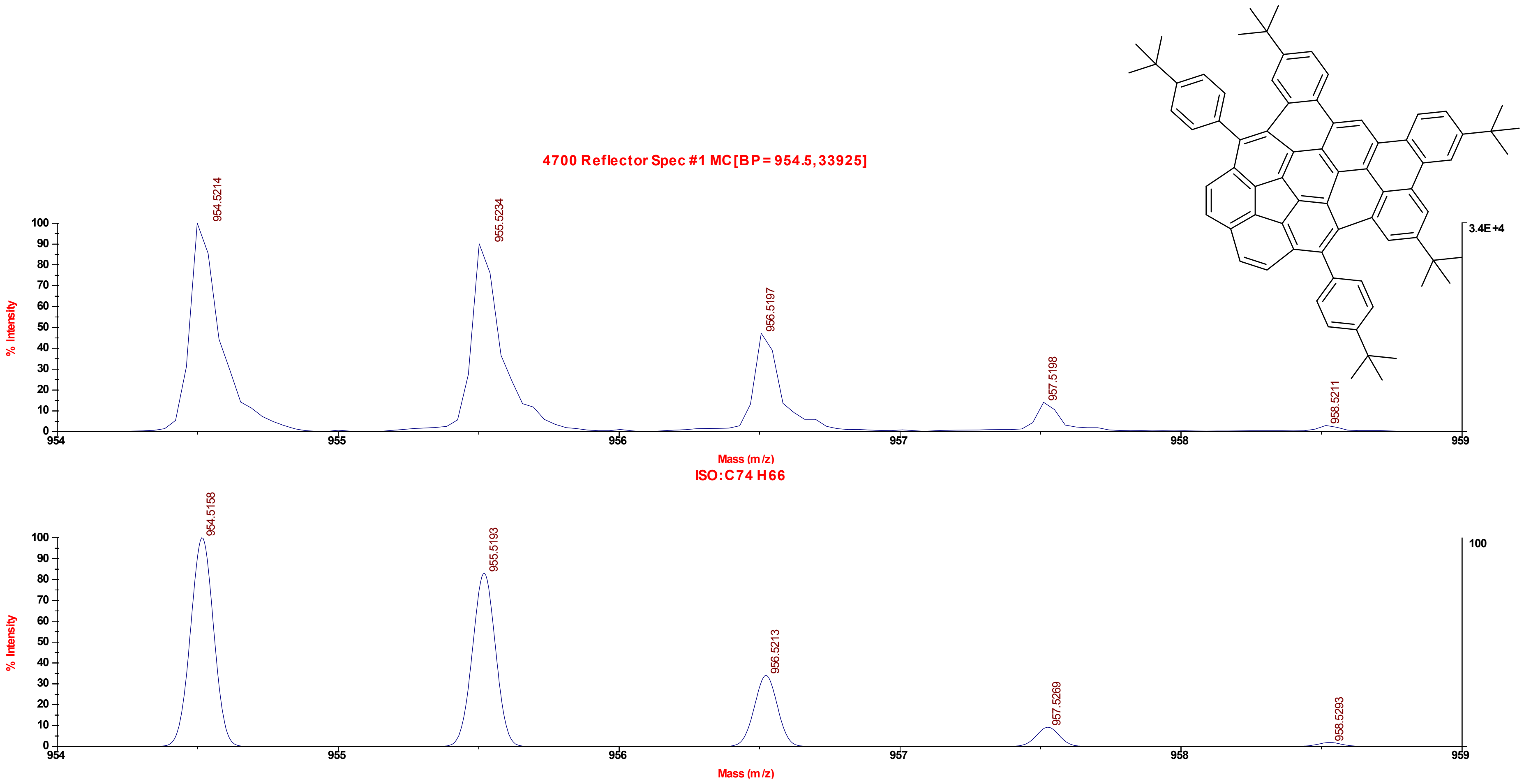

Index	Input m/z	Calc. Mass	Error (mDa)	Error (ppm)	DBE	Formula	Isotope Match Score
1	954.52136	954.51590	5.4559	5.7159	42.00	C74 H66	0.995292

Supplementary Figure 45. MALDI/TOF (HRMS) mass spectrum of **25**.

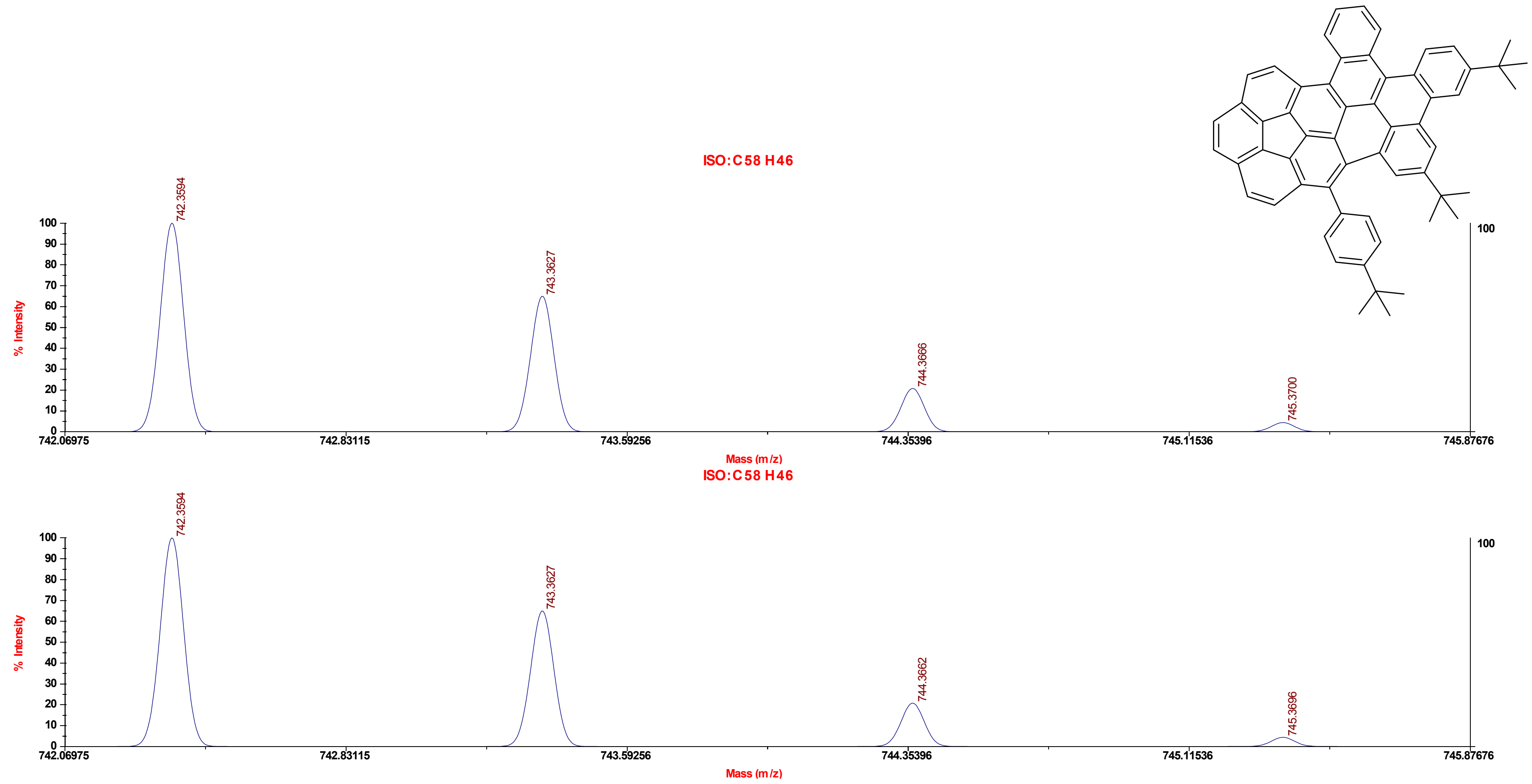

Index	Input m/z	Calc. Mass	Error (mDa)	Error (ppm)	DBE	Formula	Isotope Match Score
1	742.36481	742.35940	5.4067	7.2831	36.00	C58 H46	0.993037

Supplementary Figure 46. MALDI/TOF (HRMS) mass spectrum of **26**.

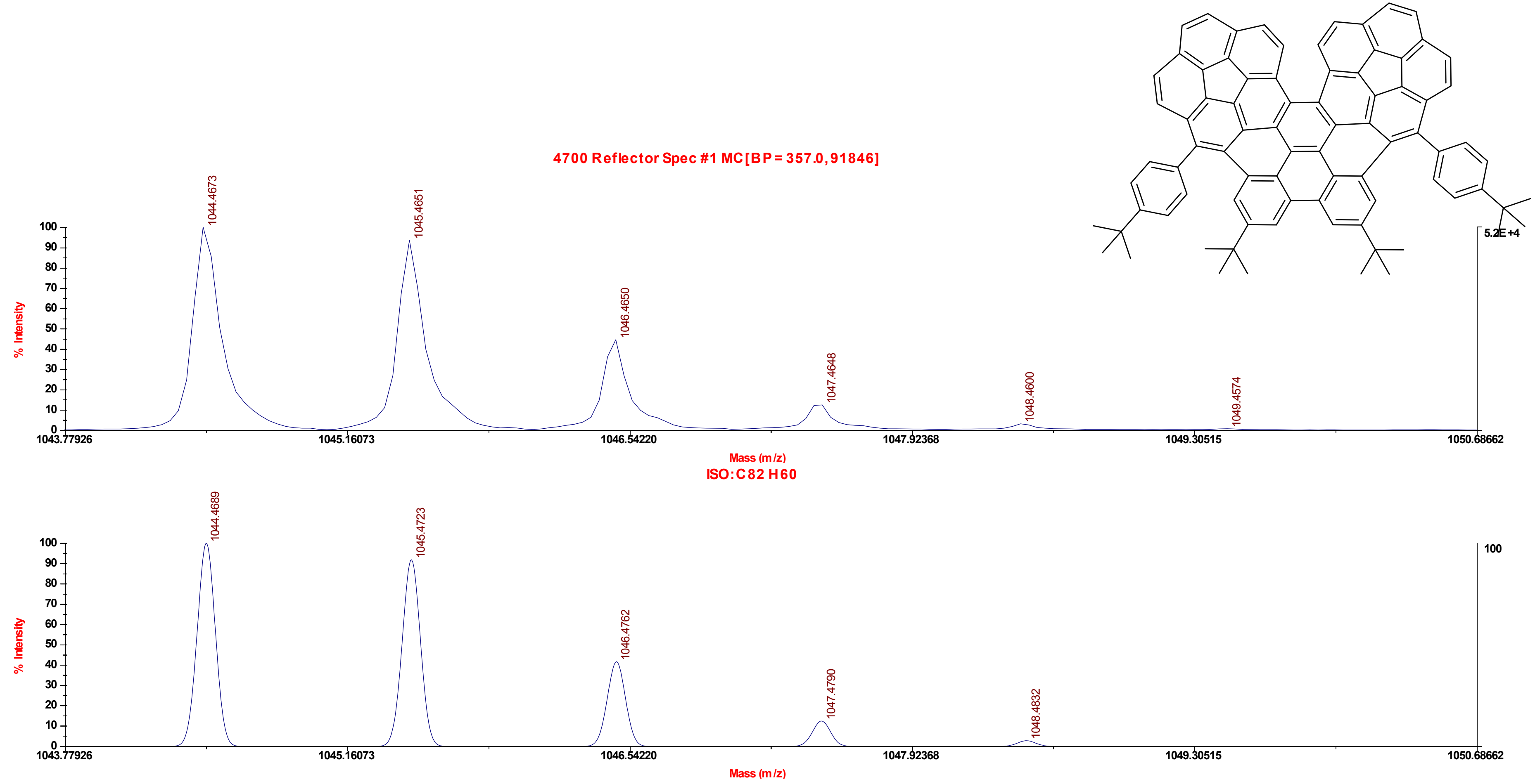

Index	Input m/z	Calc. Mass	Error (mDa)	Error (ppm)	DBE	Formula	Isotope Match Score
1	1044.46729	1044.46895	-1.6638	-1.5930	53.00	C82 H60	0.999102

Supplementary Figure 47. MALDI/TOF (HRMS) mass spectrum of **27**

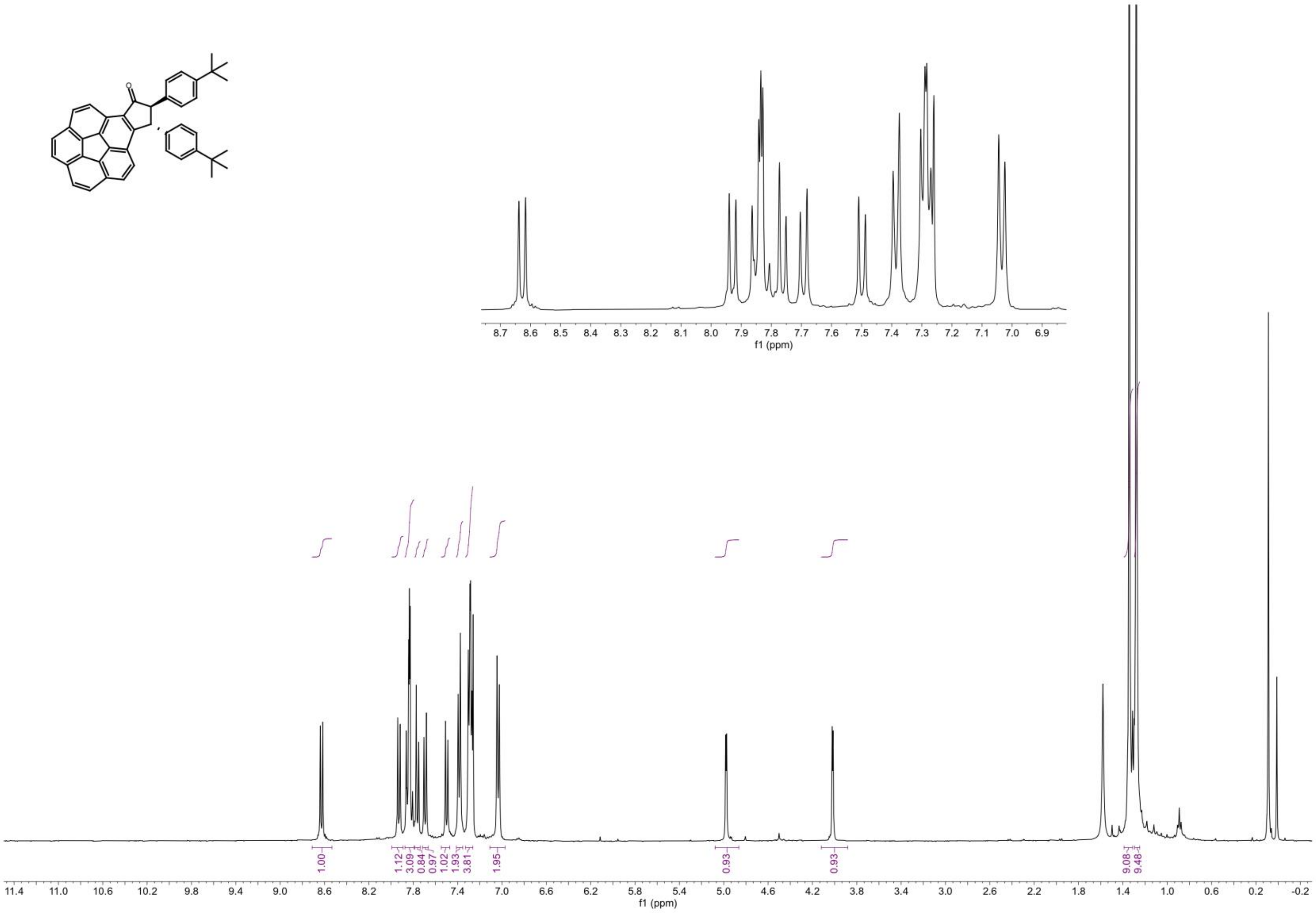

Supplementary Figure 48. NMR (400 MHz) of compound **3** in deuterated chloroform at room temperature.

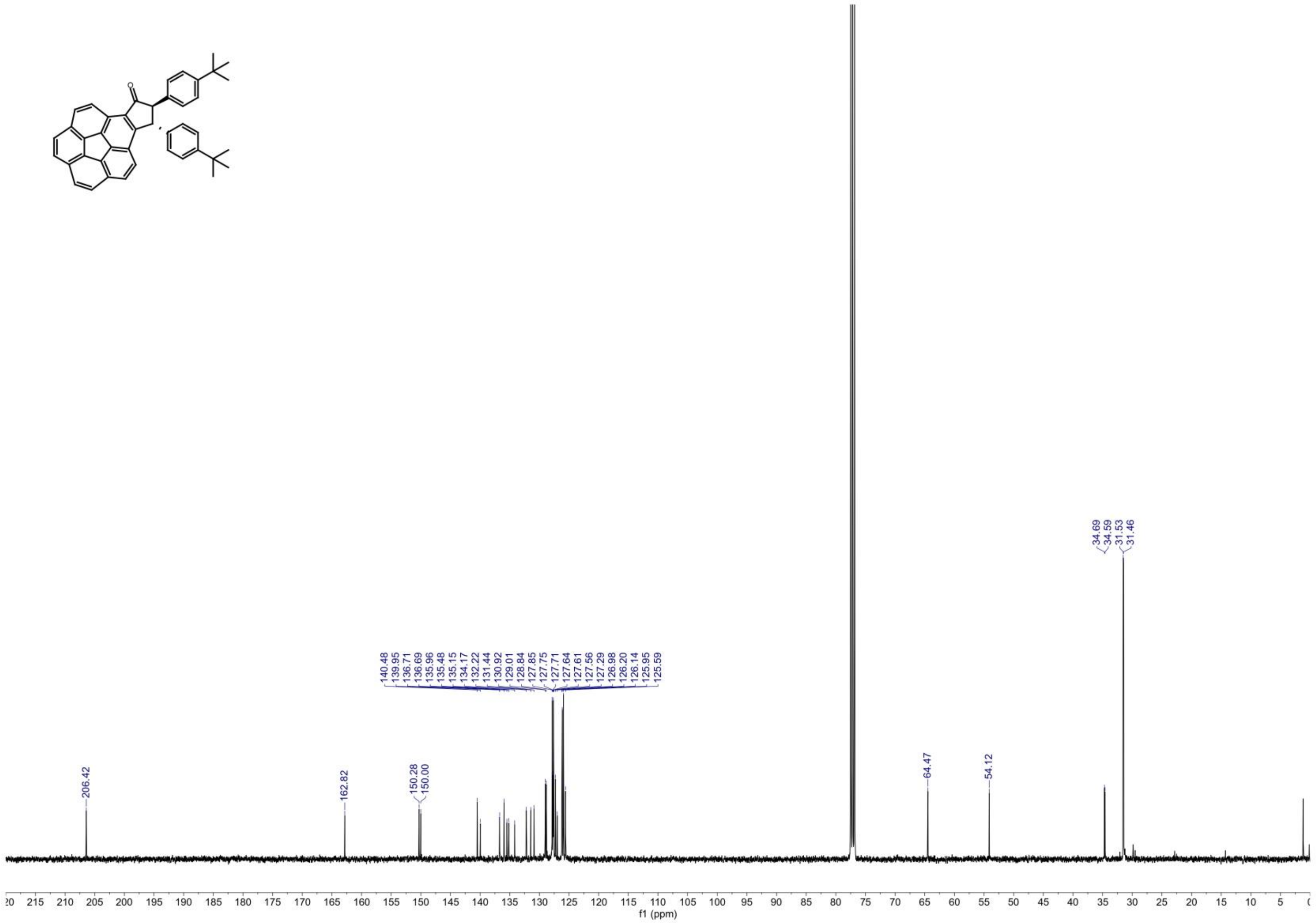

Supplementary Figure 49. NMR (101 MHz) of compound **3** in deuterated chloroform at room temperature.

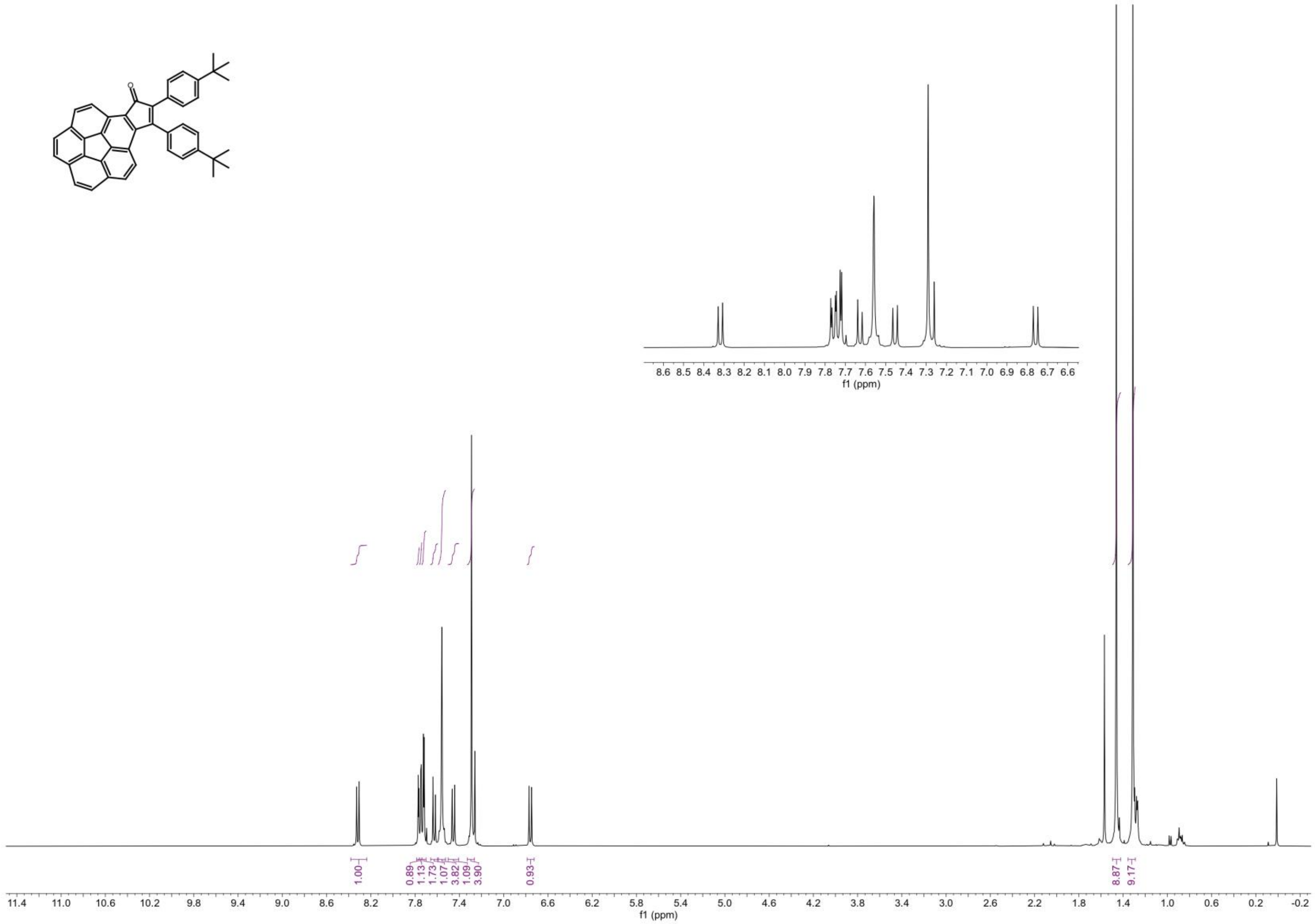

Supplementary Figure 50. NMR (400 MHz) of compound **4** in deuterated chloroform at room temperature.

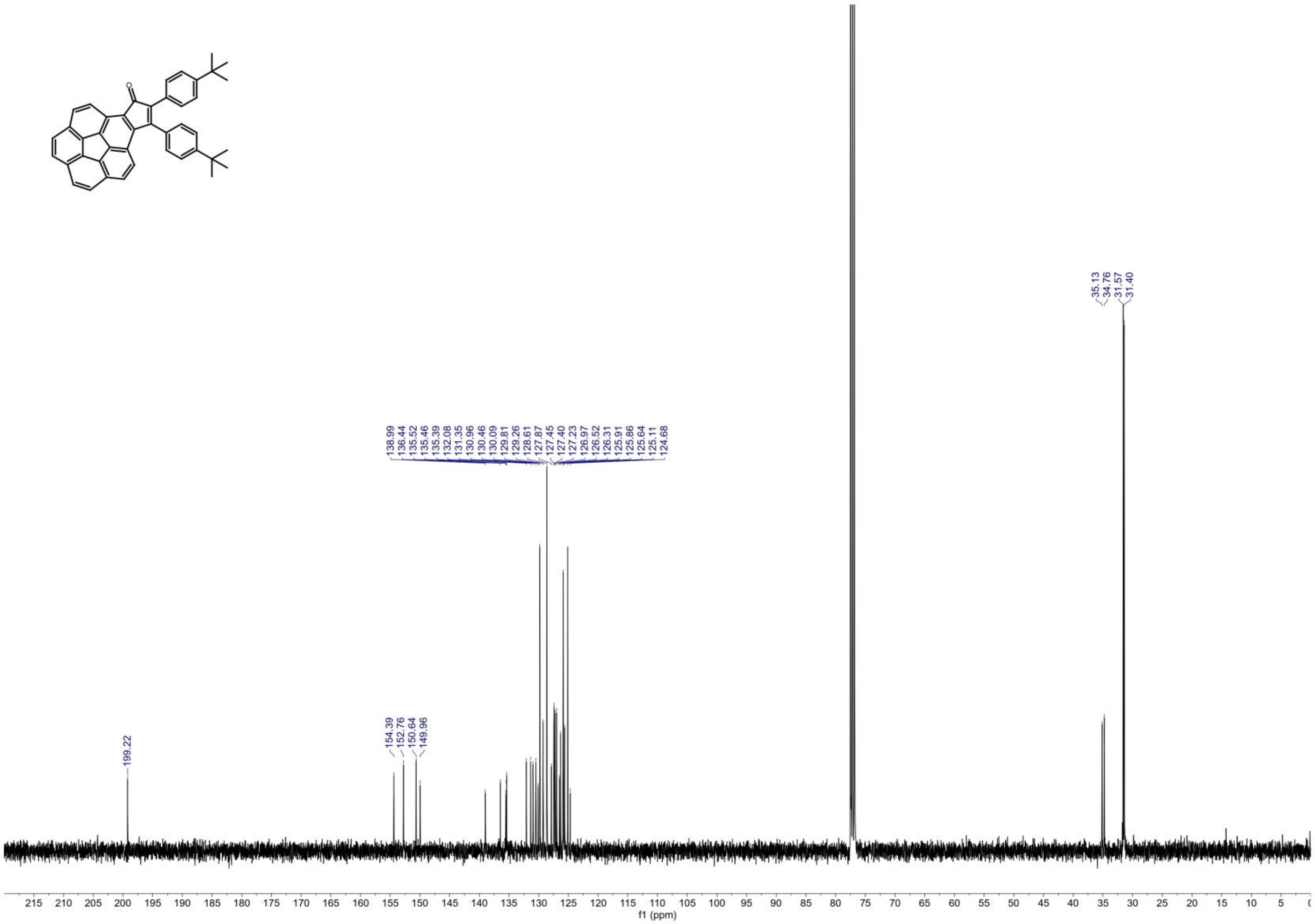

Supplementary Figure 51. NMR (101 MHz) of compound **4** in deuterated chloroform at room temperature.

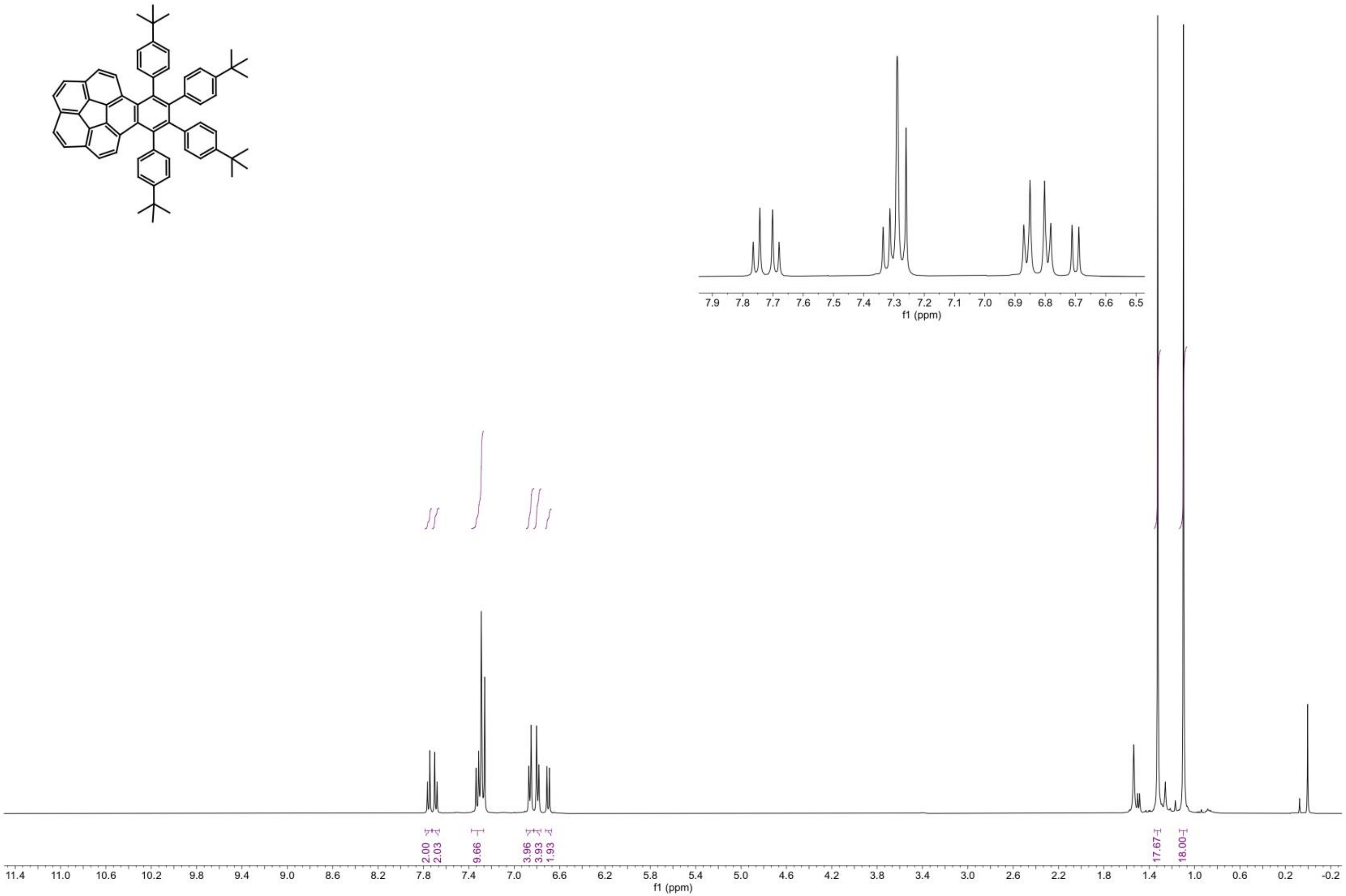

Supplementary Figure 52. NMR (400 MHz) of compound **6** in deuterated chloroform at room temperature.

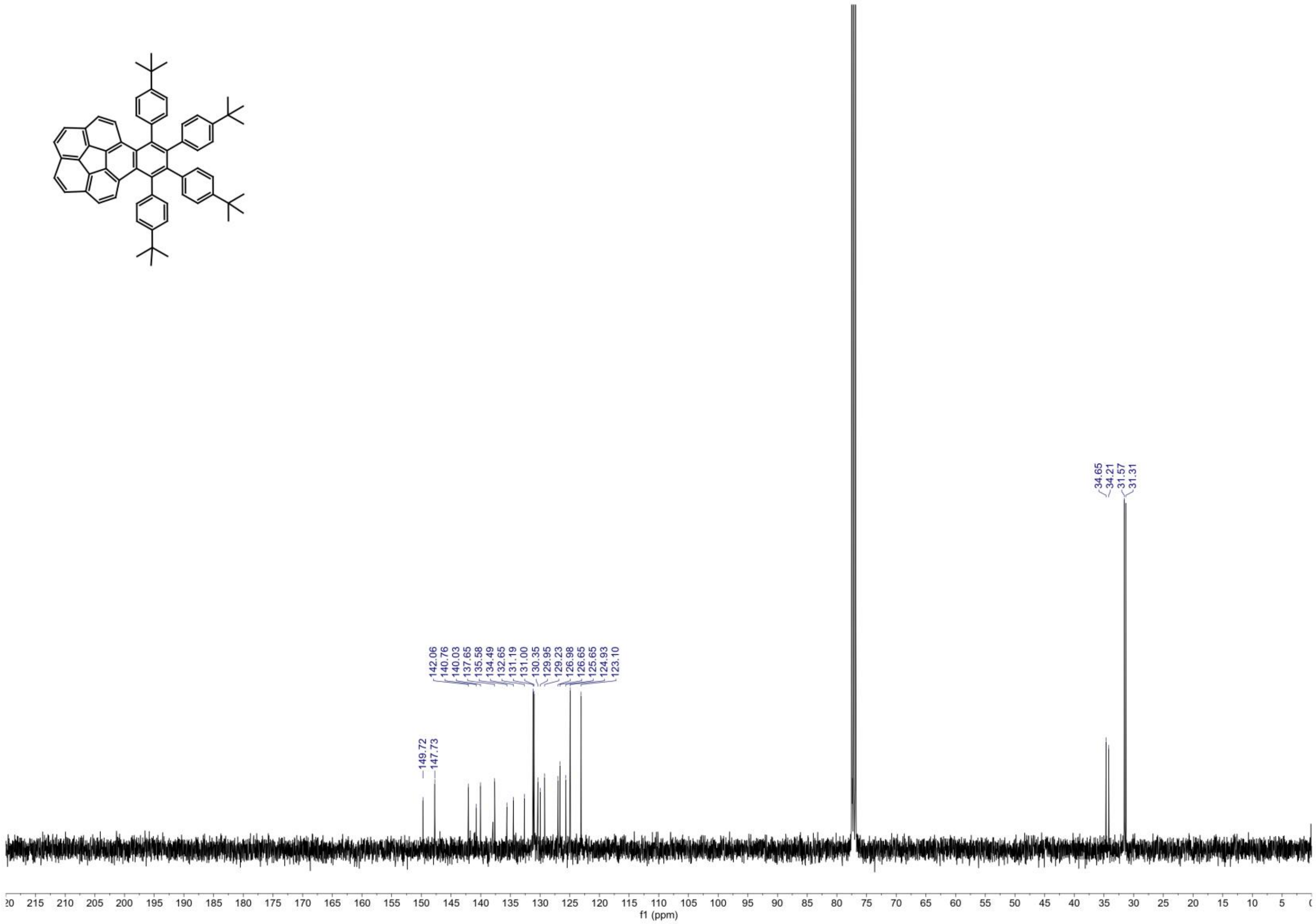

Supplementary Figure 53. NMR (101 MHz) of compound **6** in deuterated chloroform at room temperature.

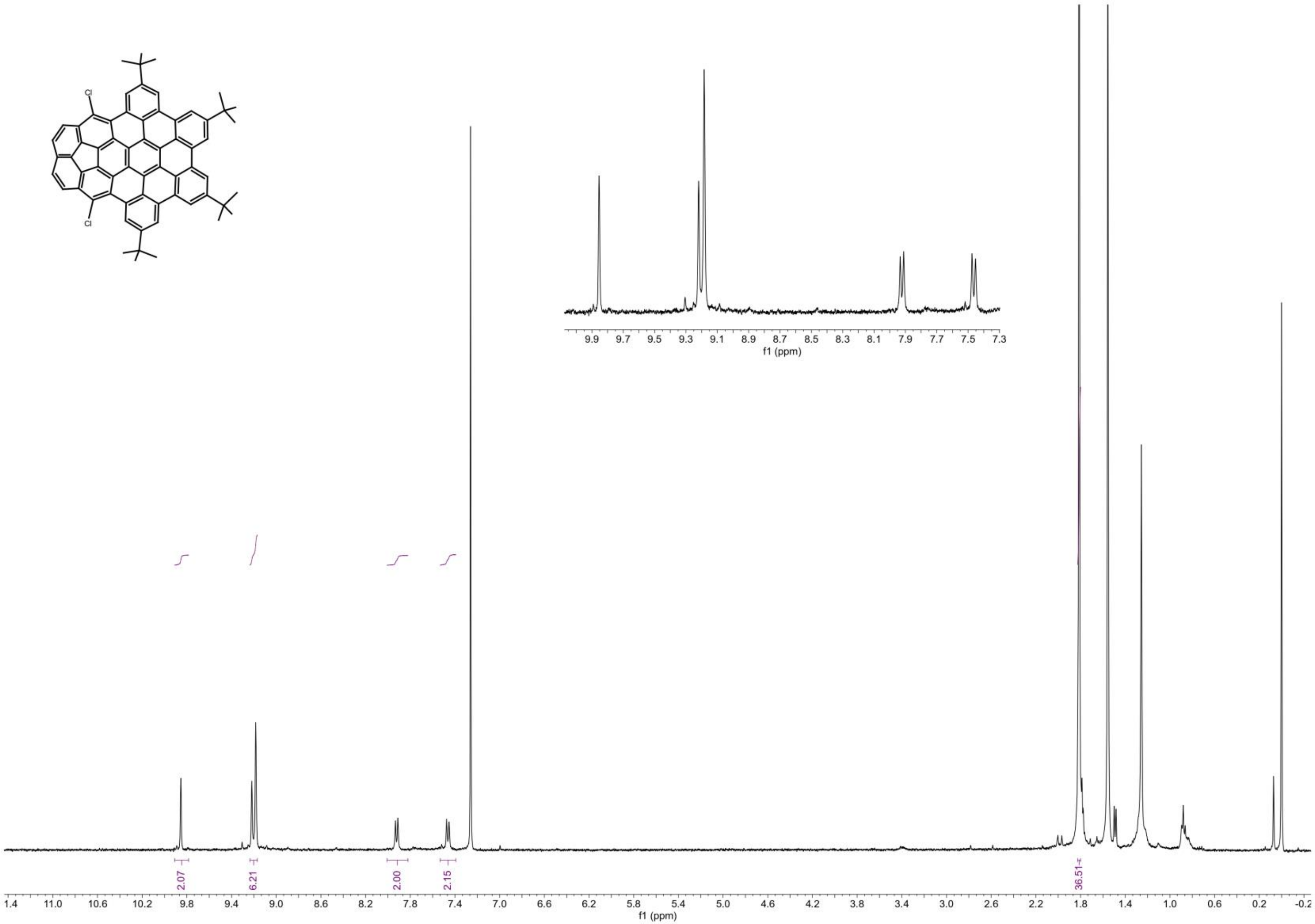

Supplementary Figure 54. NMR (400 MHz) of compound 7 in deuterated chloroform at room temperature.

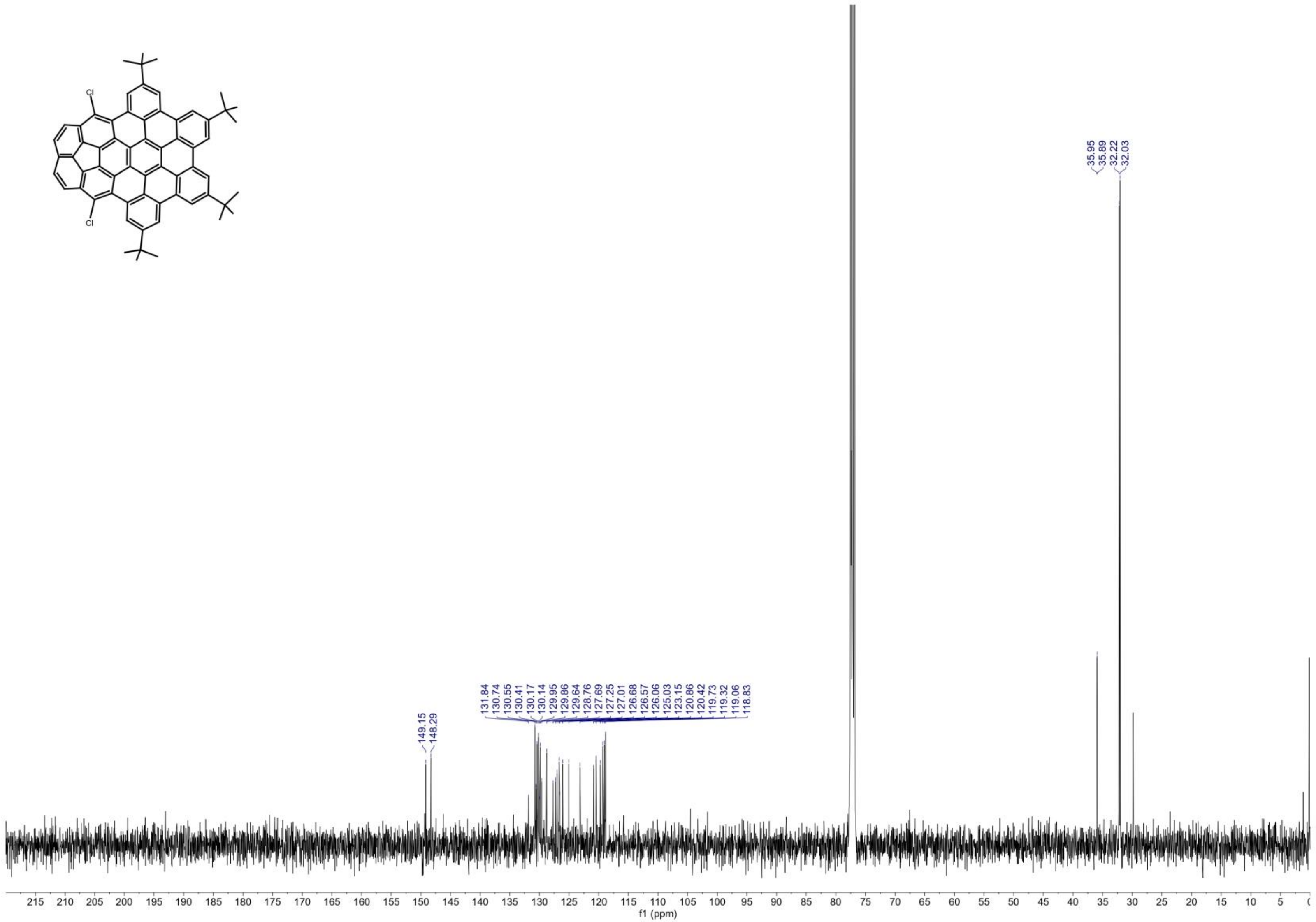

Supplementary Figure 55. NMR (101 MHz) of compound **7** in deuterated chloroform at room temperature.

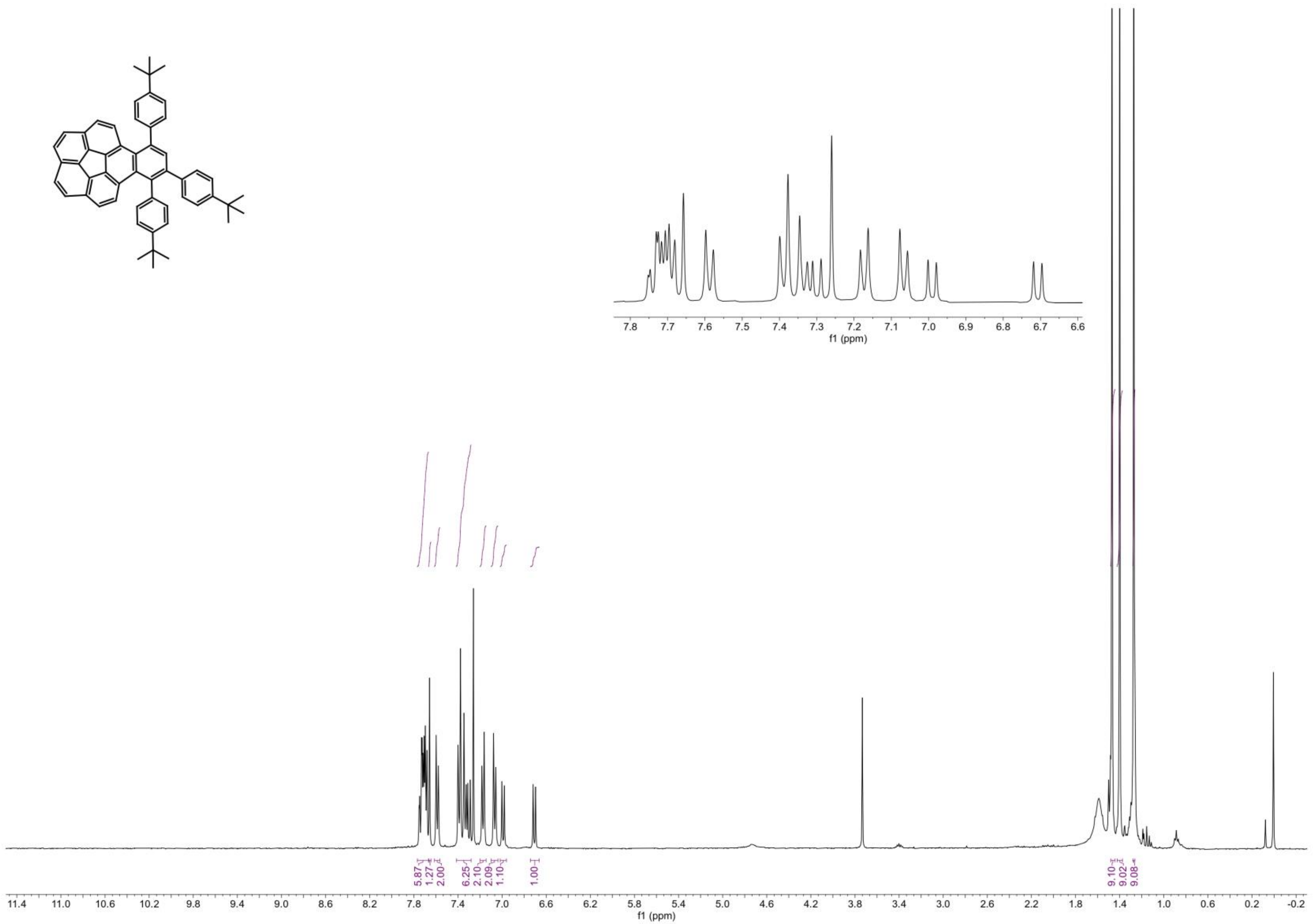

Supplementary Figure 56. NMR (400 MHz) of compound **10** in deuterated chloroform at room temperature.

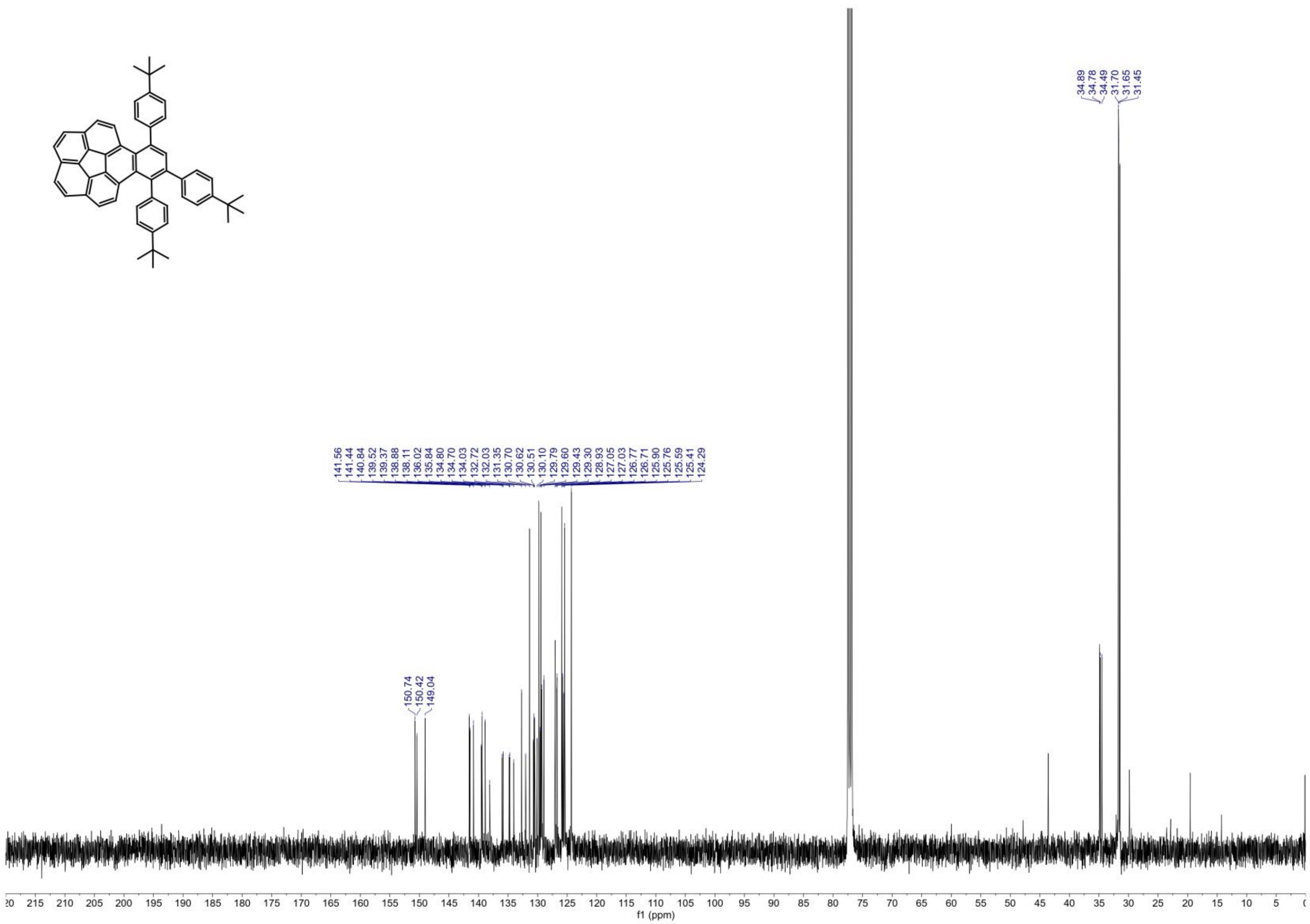

Supplementary Figure 57. NMR (101 MHz) of compound **10** in deuterated chloroform at room temperature.

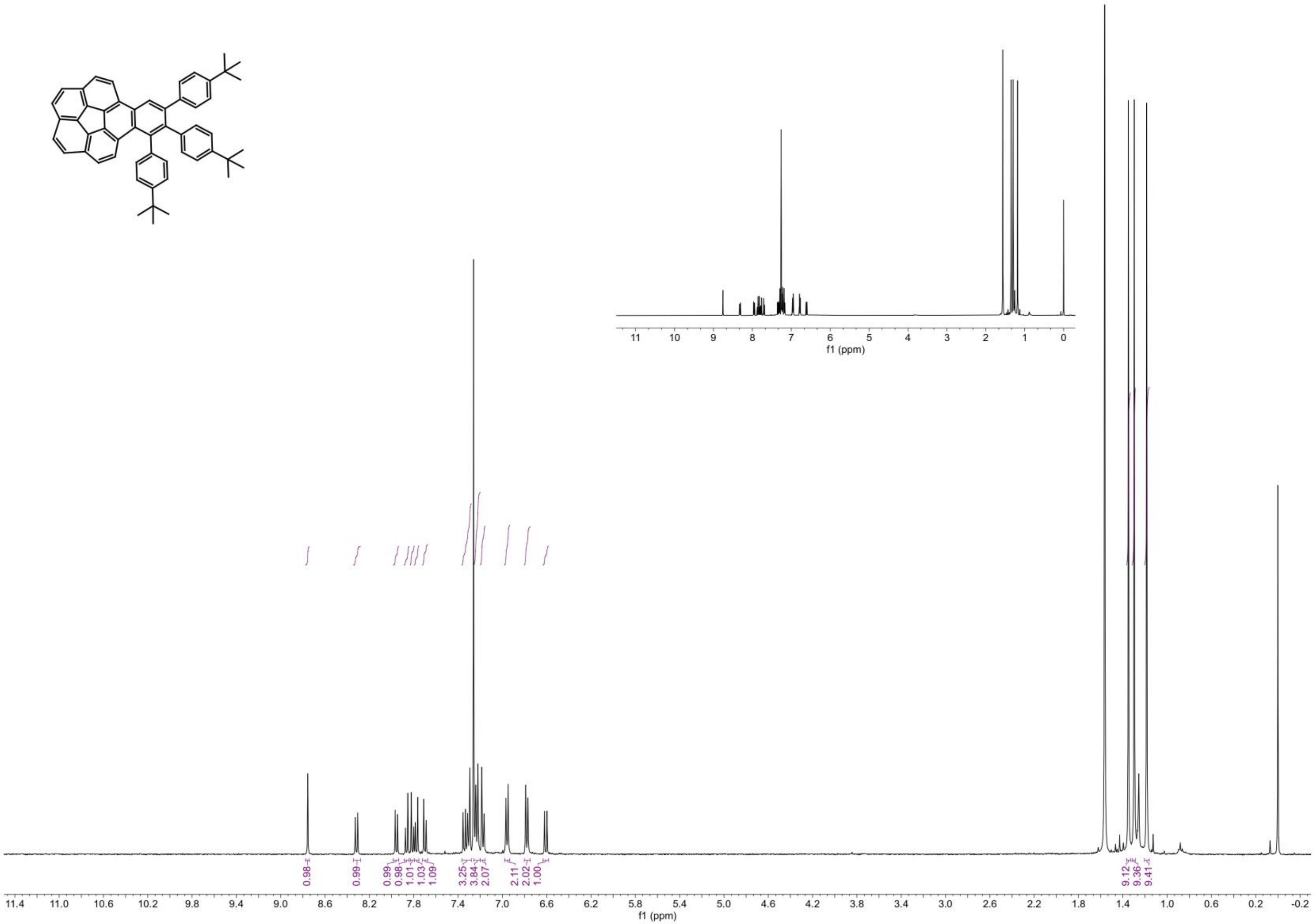

Supplementary Figure 58. NMR (400 MHz) of compound **9** in deuterated chloroform at room temperature.

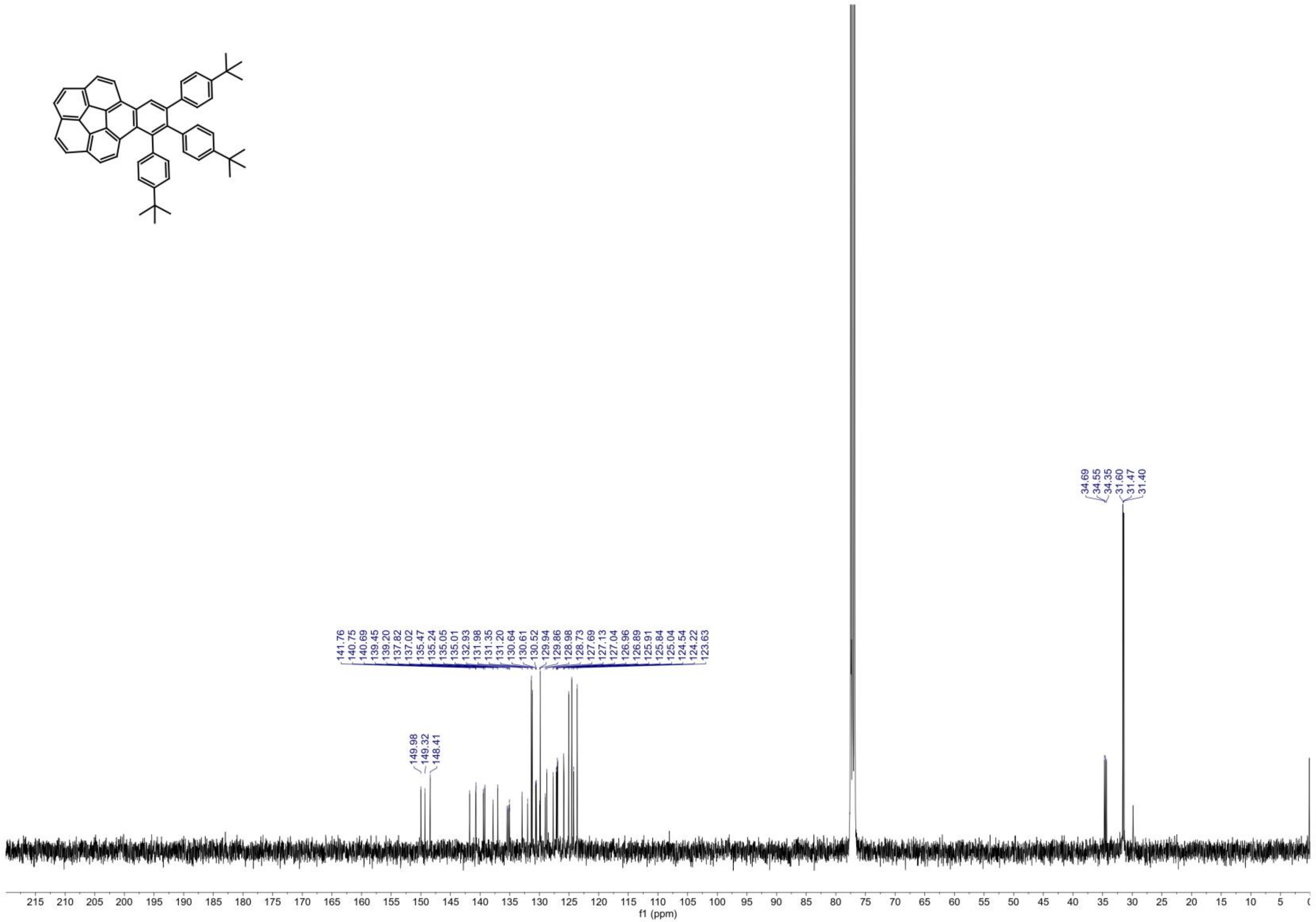

Supplementary Figure 59. NMR (101 MHz) of compound **9** in deuterated chloroform at room temperature.

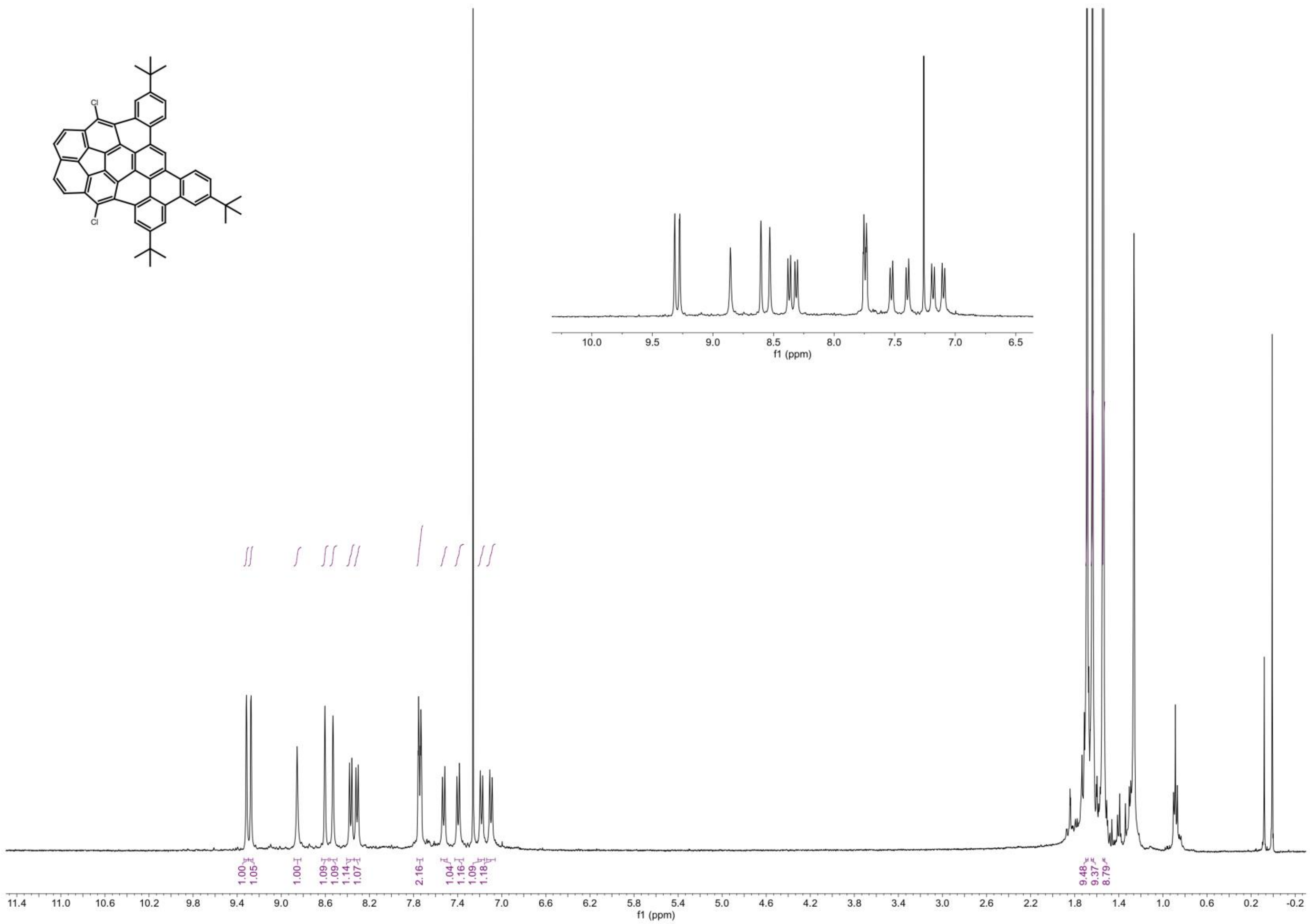

Supplementary Figure 60. NMR (400 MHz) of compound **12** in deuterated chloroform at room temperature.

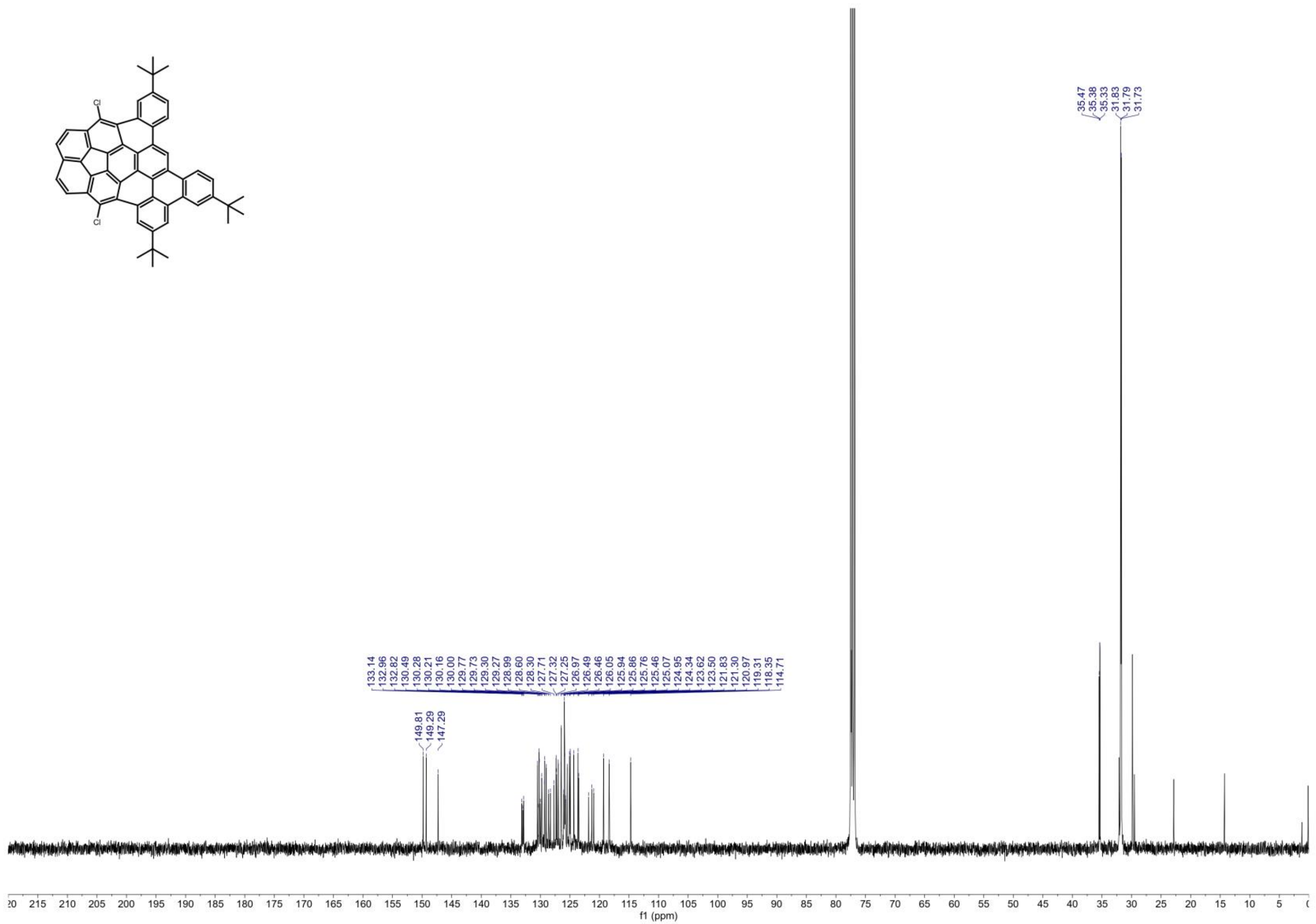

Supplementary Figure 61. NMR (101 MHz) of compound **12** in deuterated chloroform at room temperature.

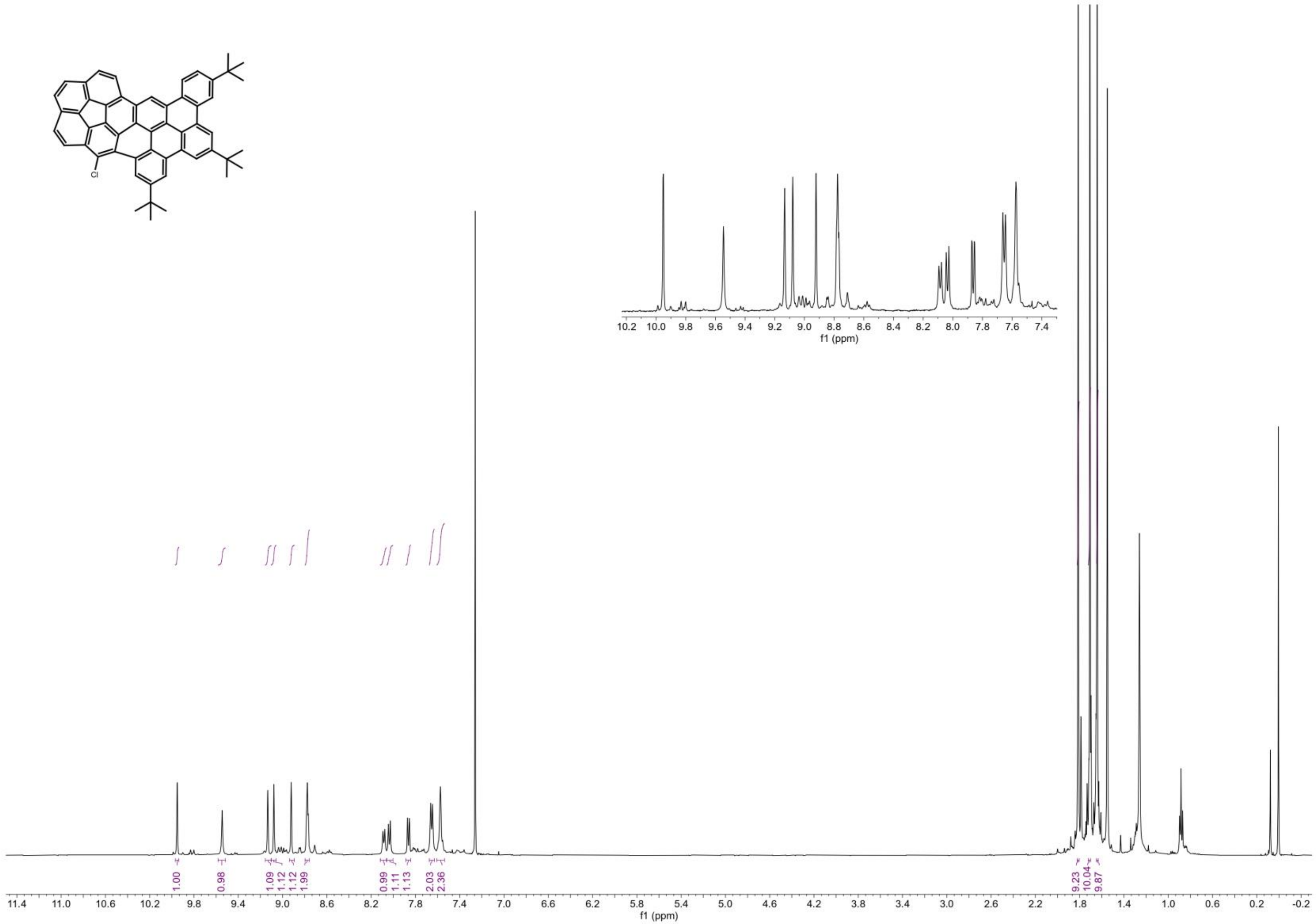

Supplementary Figure 62. NMR (500 MHz) of compound **11** in deuterated chloroform at room temperature.

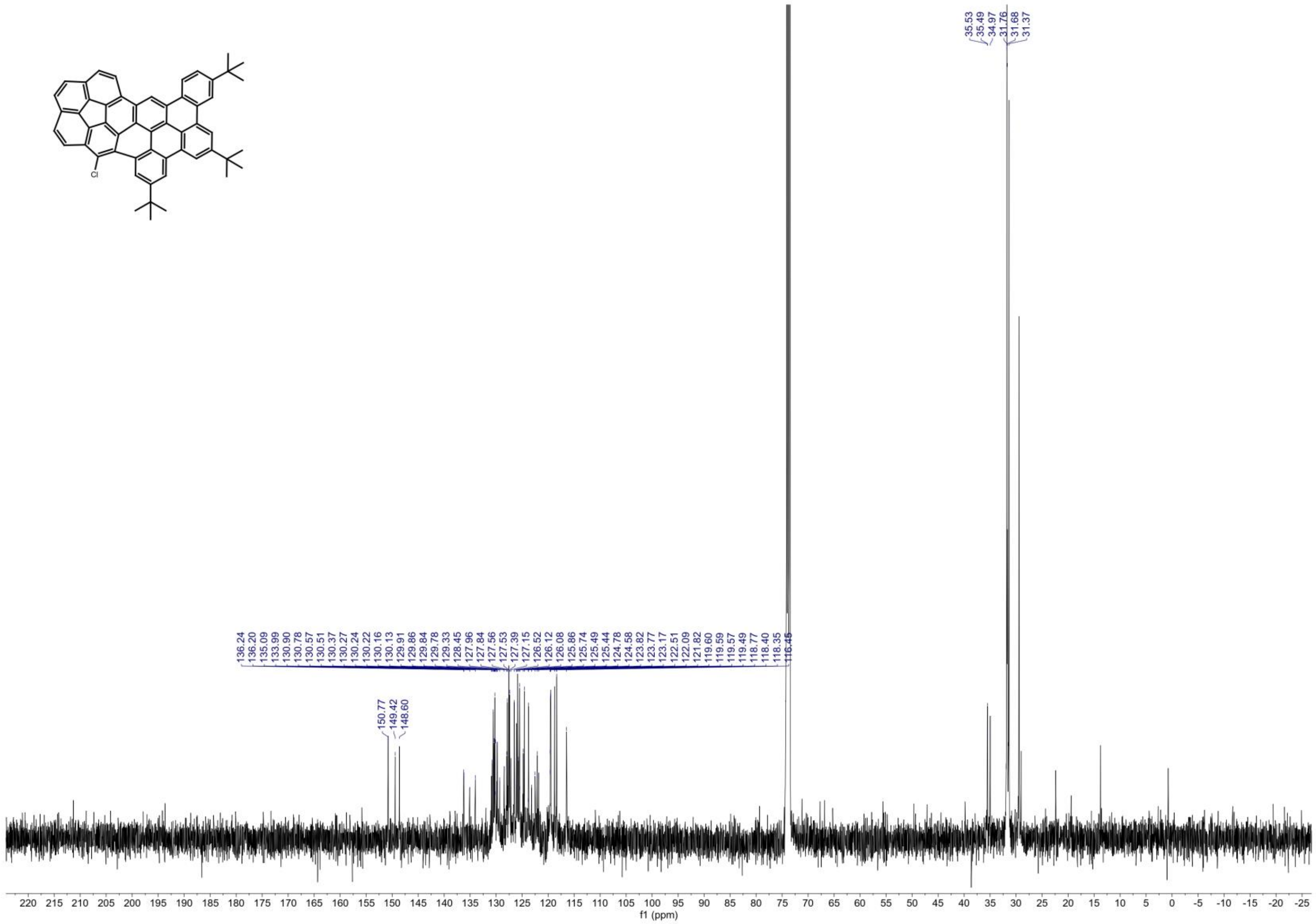

Supplementary Figure 63. NMR (100 MHz) of compound **11** in deuterated dichloromethane at room temperature.

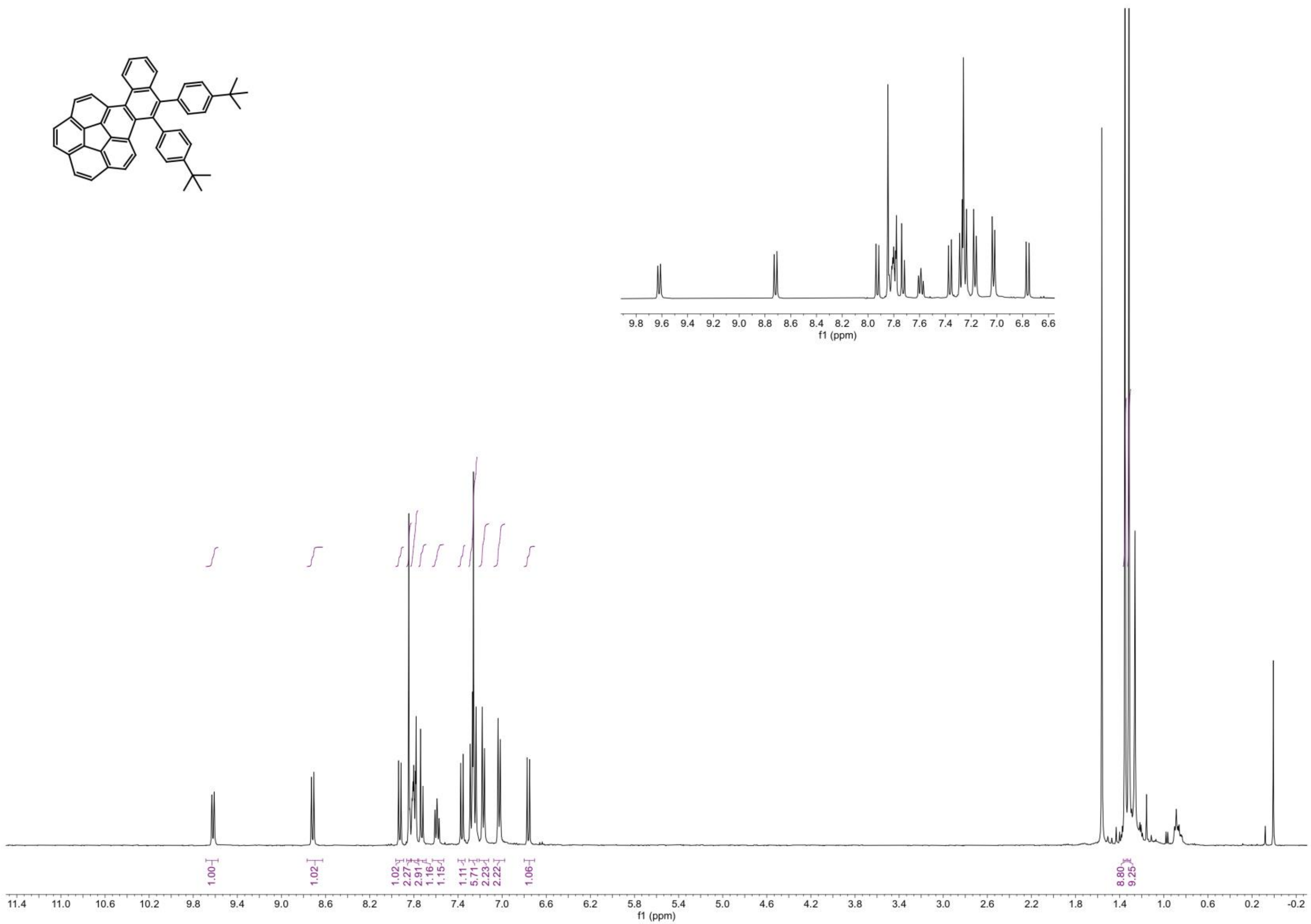

Supplementary Figure 64. NMR (400 MHz) of compound **15** in deuterated chloroform at room temperature.

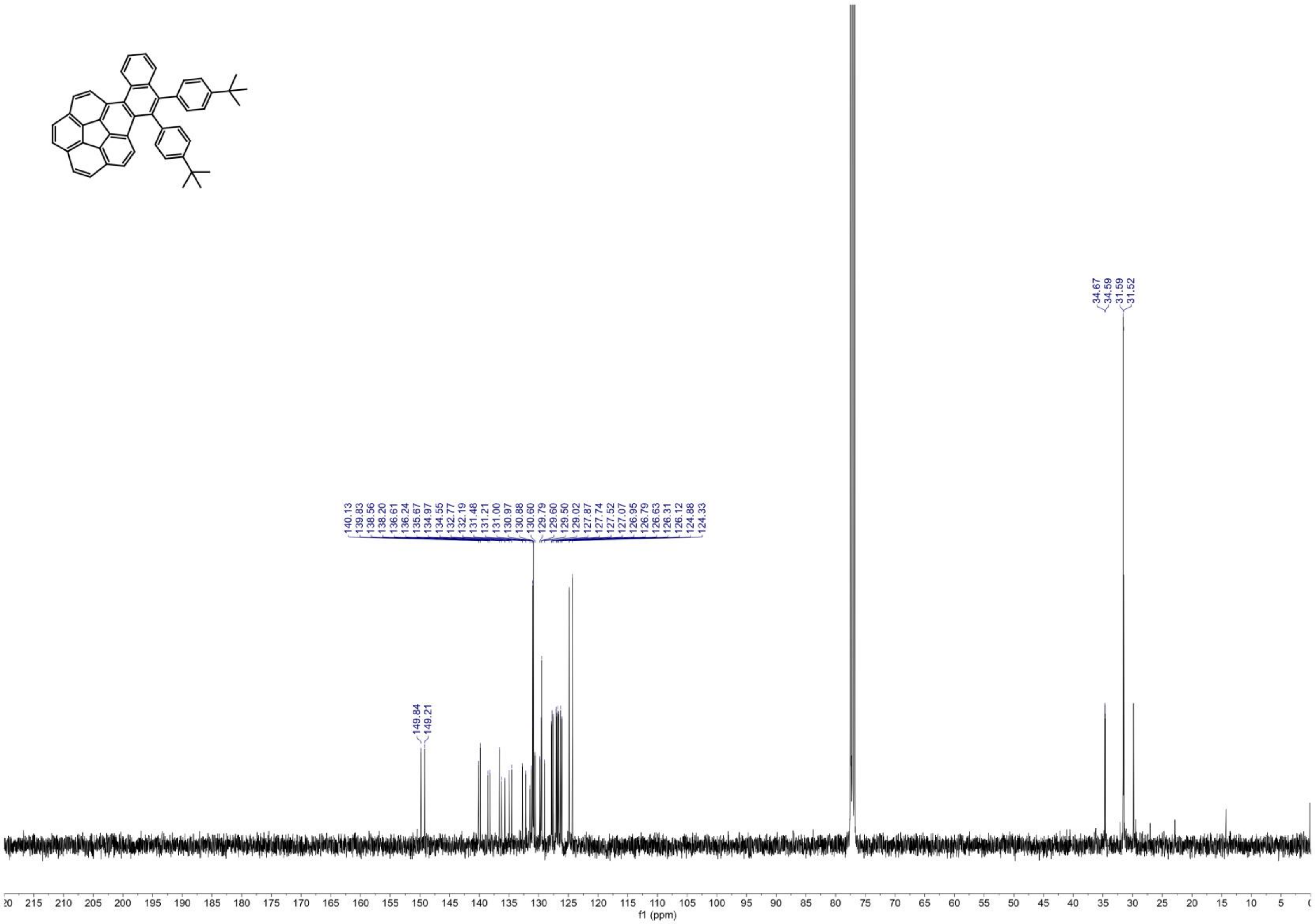

Supplementary Figure 65. NMR (101 MHz) of compound **15** in deuterated chloroform at room temperature.

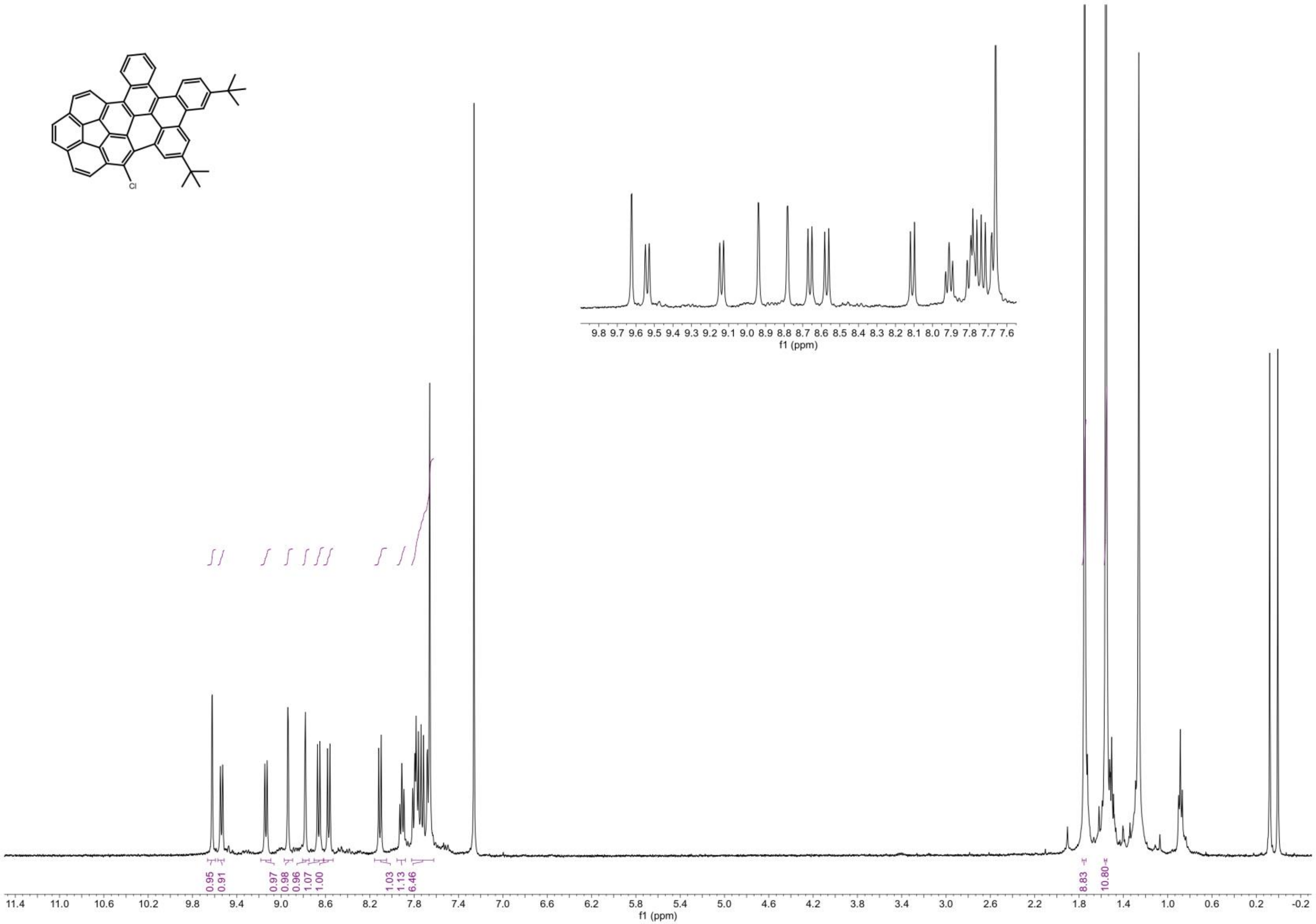

Supplementary Figure 66. NMR (400 MHz) of compound **16** in deuterated chloroform at room temperature.

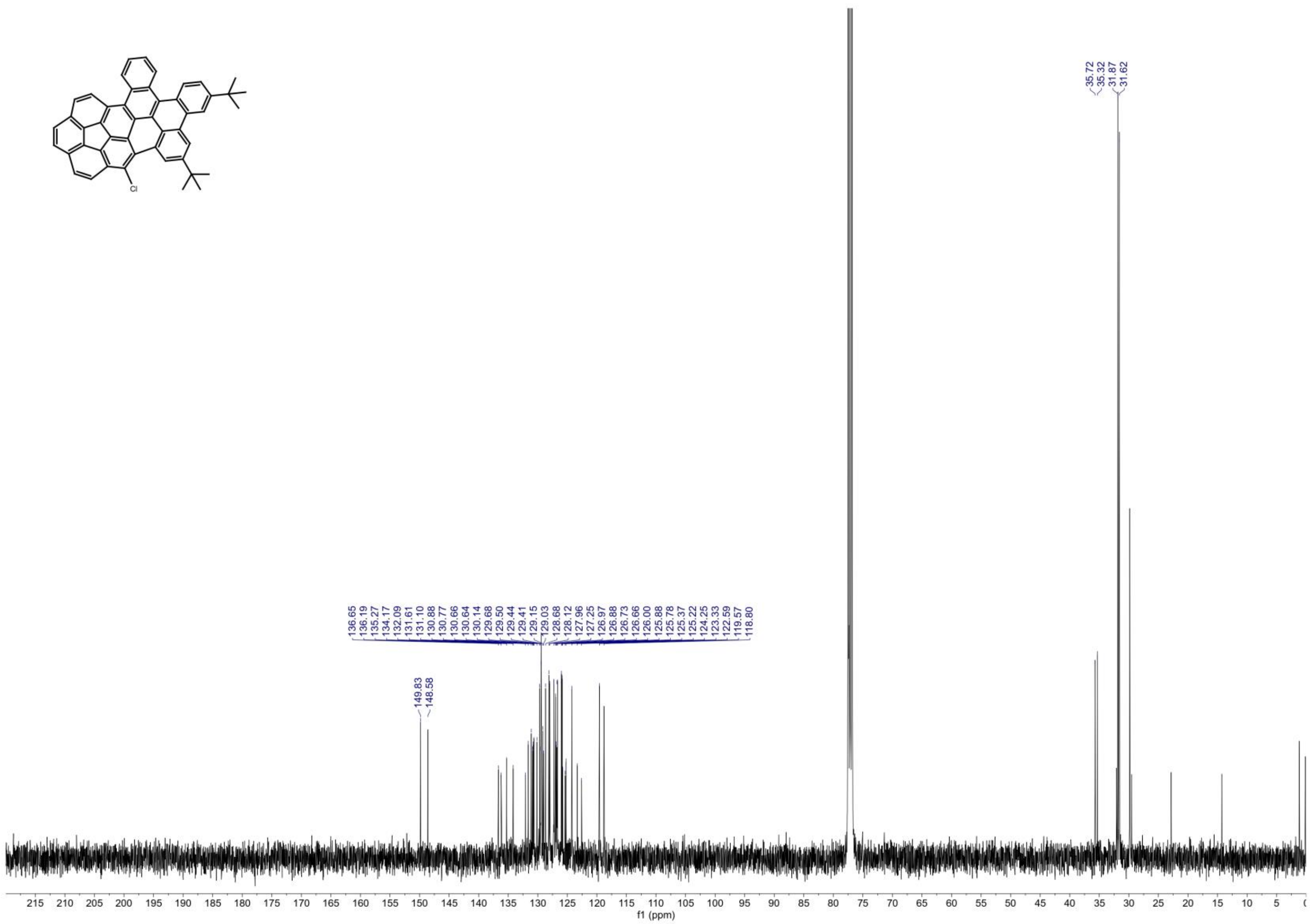

Supplementary Figure 67. NMR (101 MHz) of compound **16** in deuterated chloroform at room temperature.

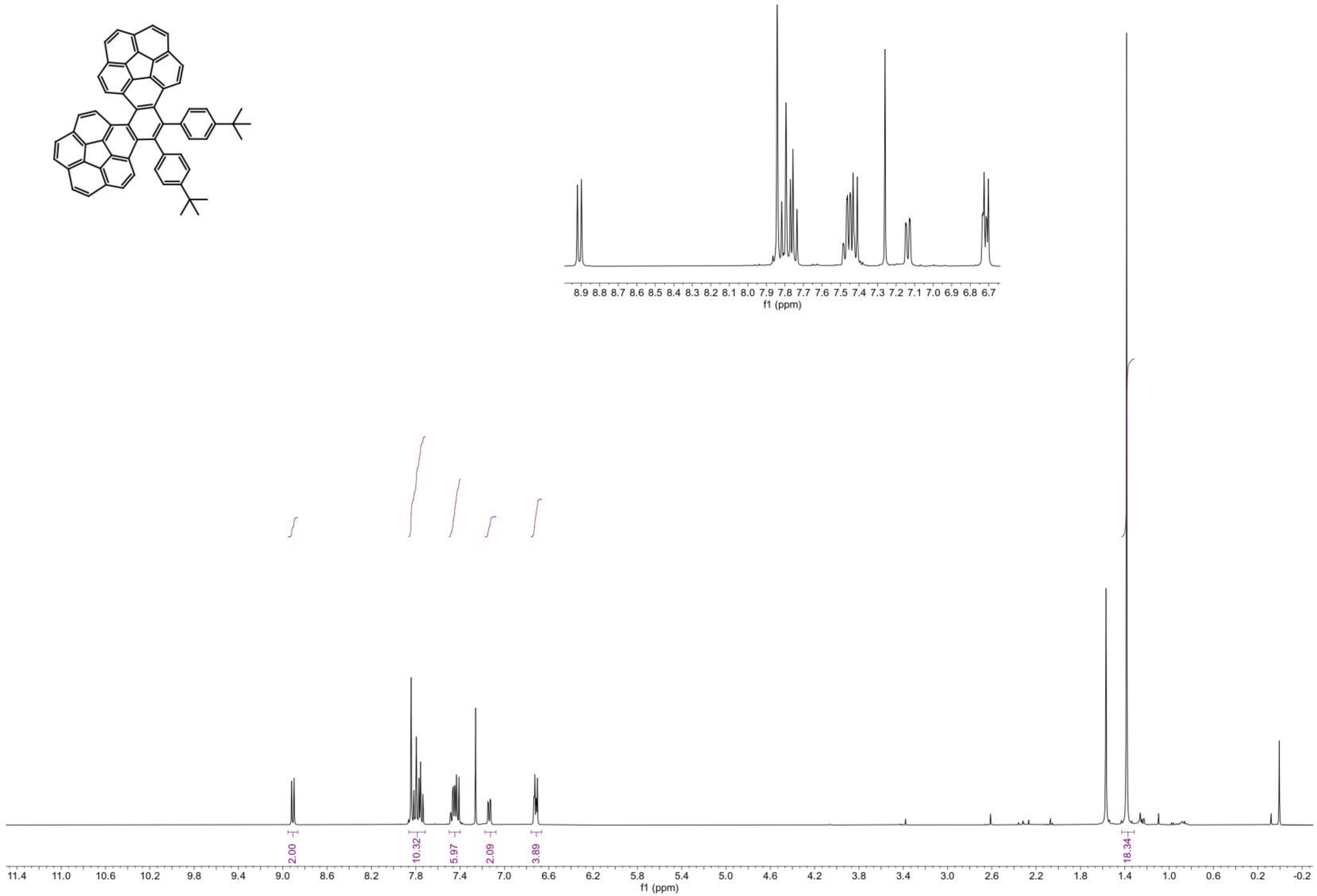

Supplementary Figure 68. NMR (400 MHz) of compound **17** in deuterated chloroform at room temperature.

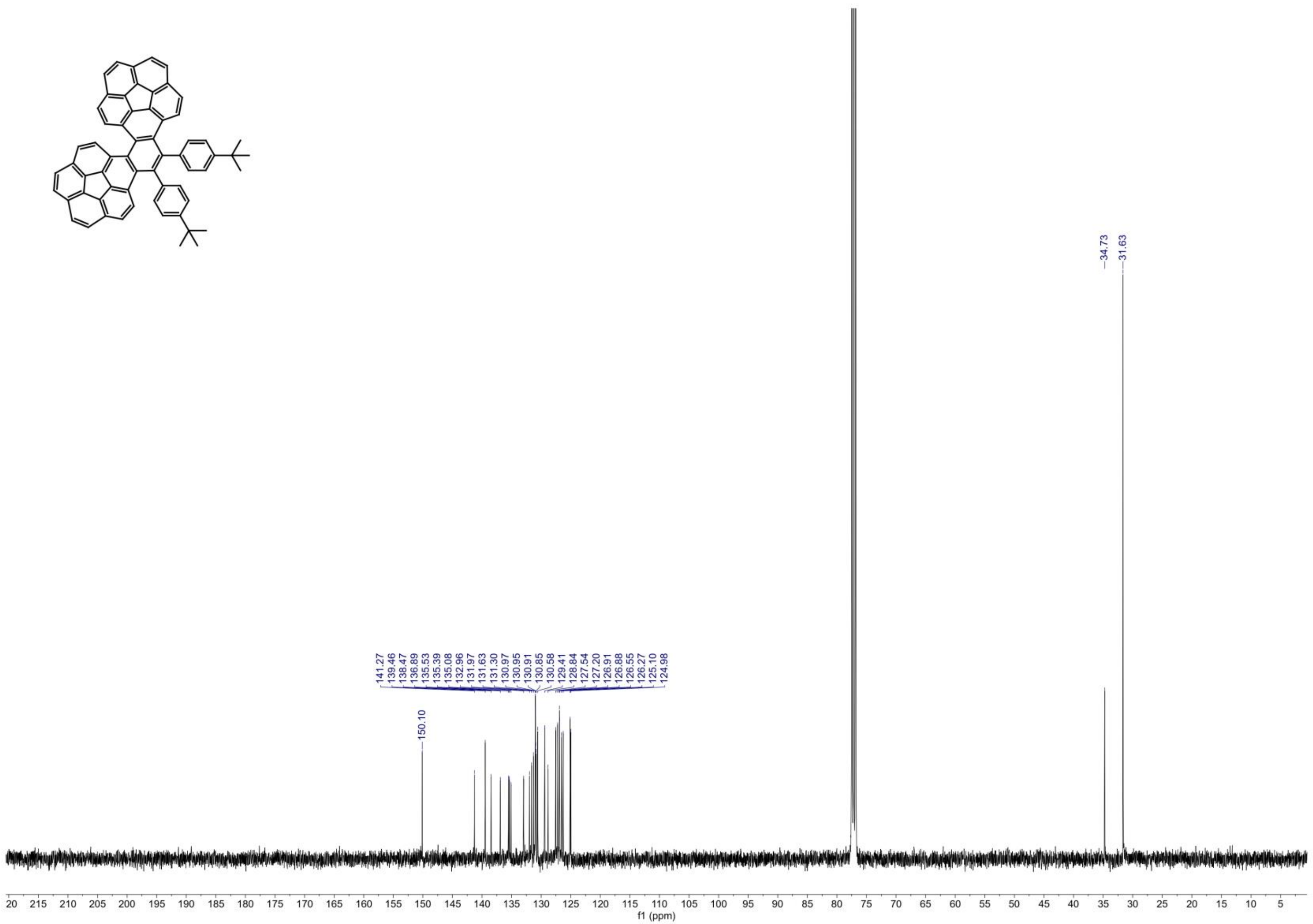

Supplementary Figure 69. NMR (101 MHz) of compound **17** in deuterated chloroform at room temperature.

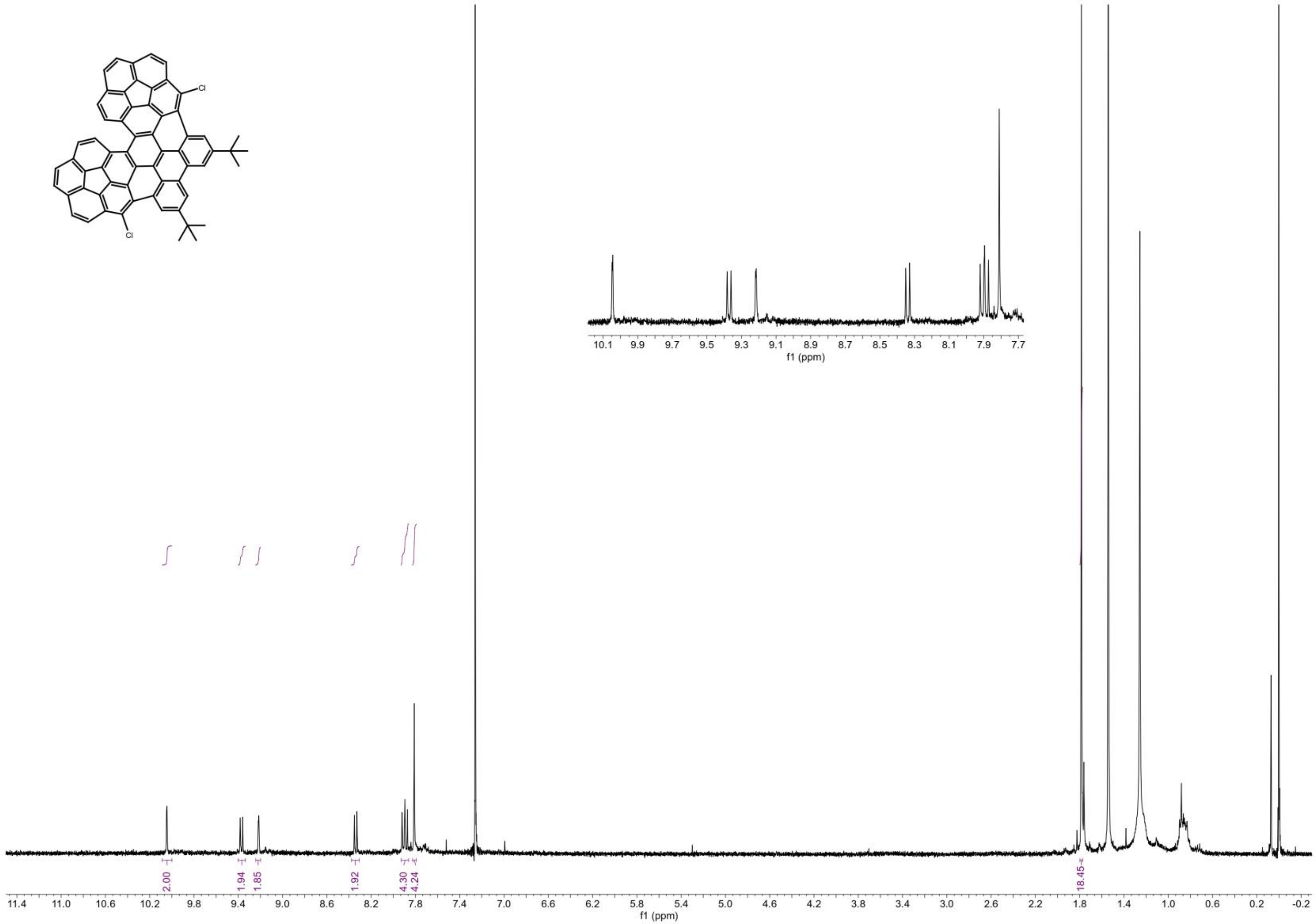

Supplementary Figure 70. NMR (400 MHz) of compound **18** in deuterated chloroform at room temperature.

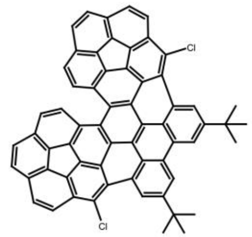

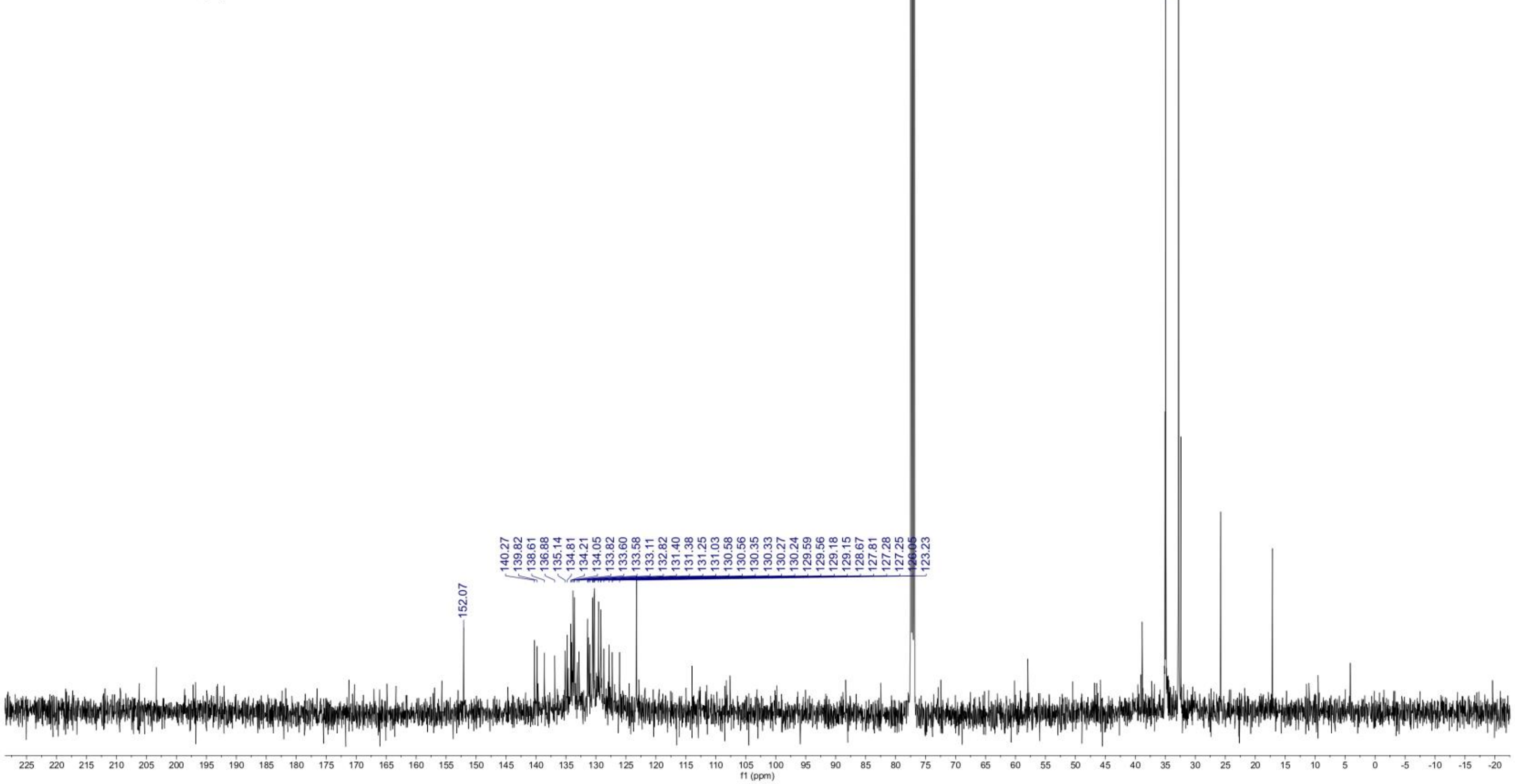

Supplementary Figure 71. NMR (100 MHz) of compound **18** in deuterated 1,1,2,2-tetrachloroethane at room temperature.

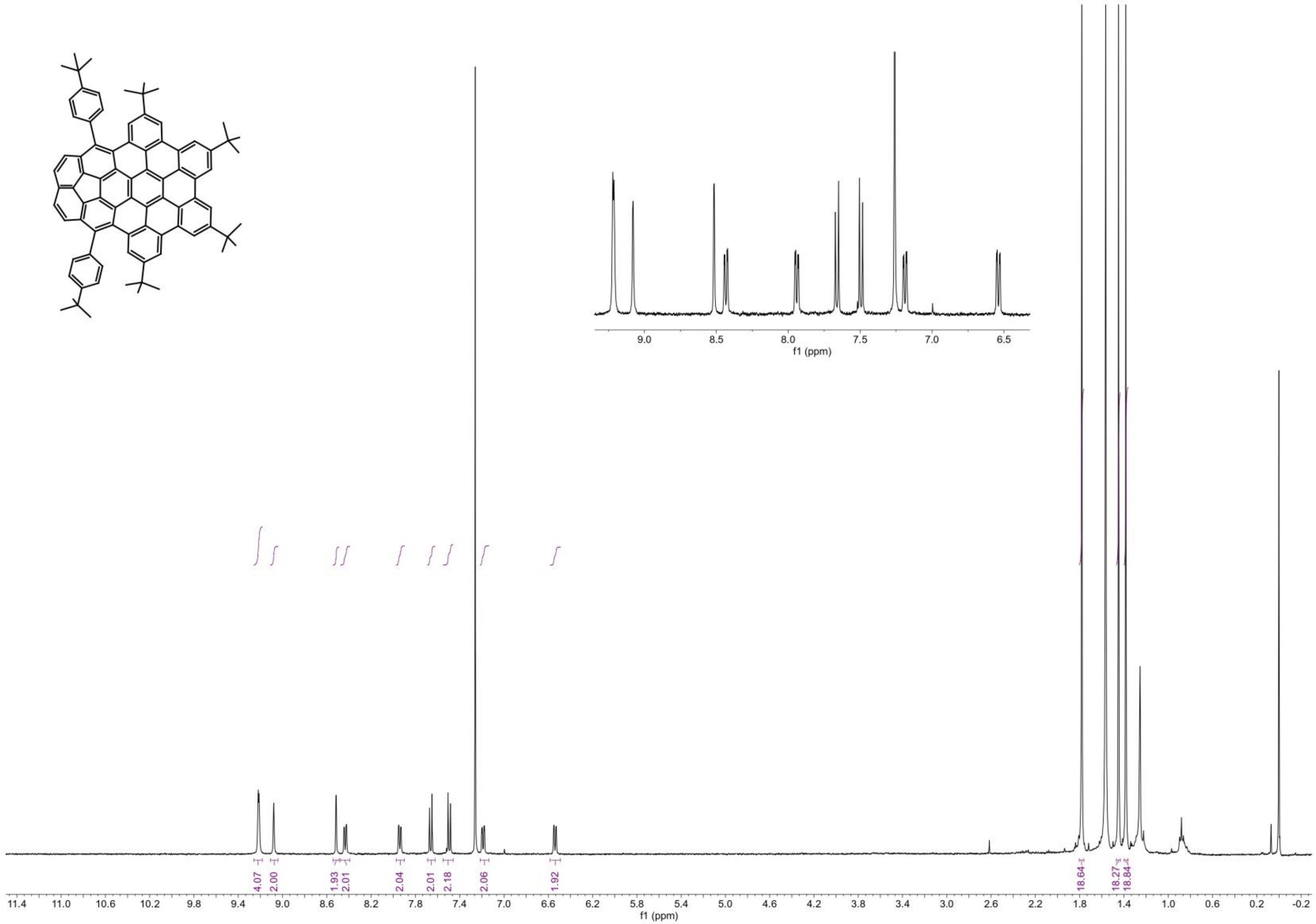

Supplementary Figure 72. NMR (400 MHz) of compound **20** in deuterated chloroform at room temperature.

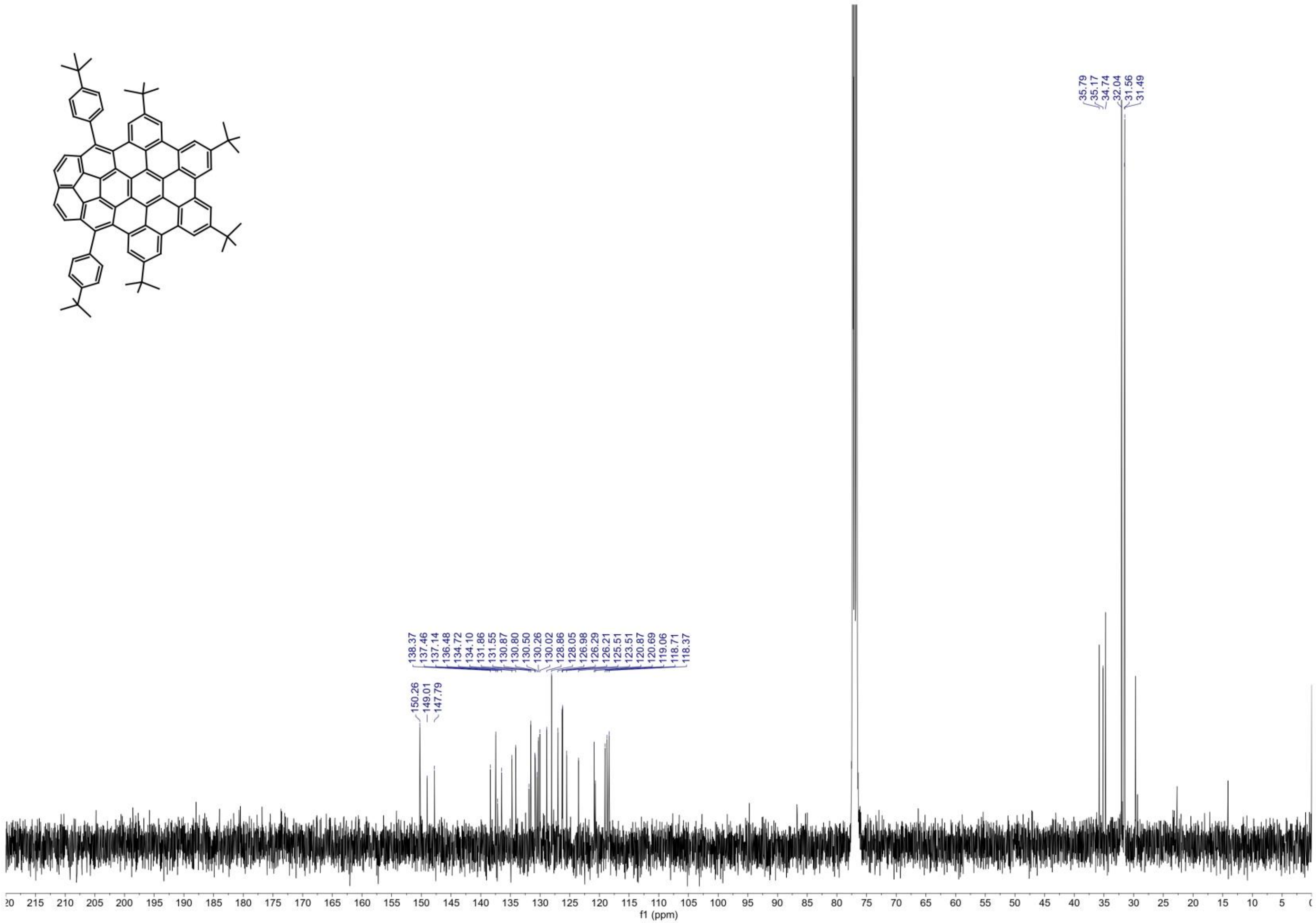

Supplementary Figure 73. NMR (101 MHz) of compound **20** in deuterated chloroform at room temperature.

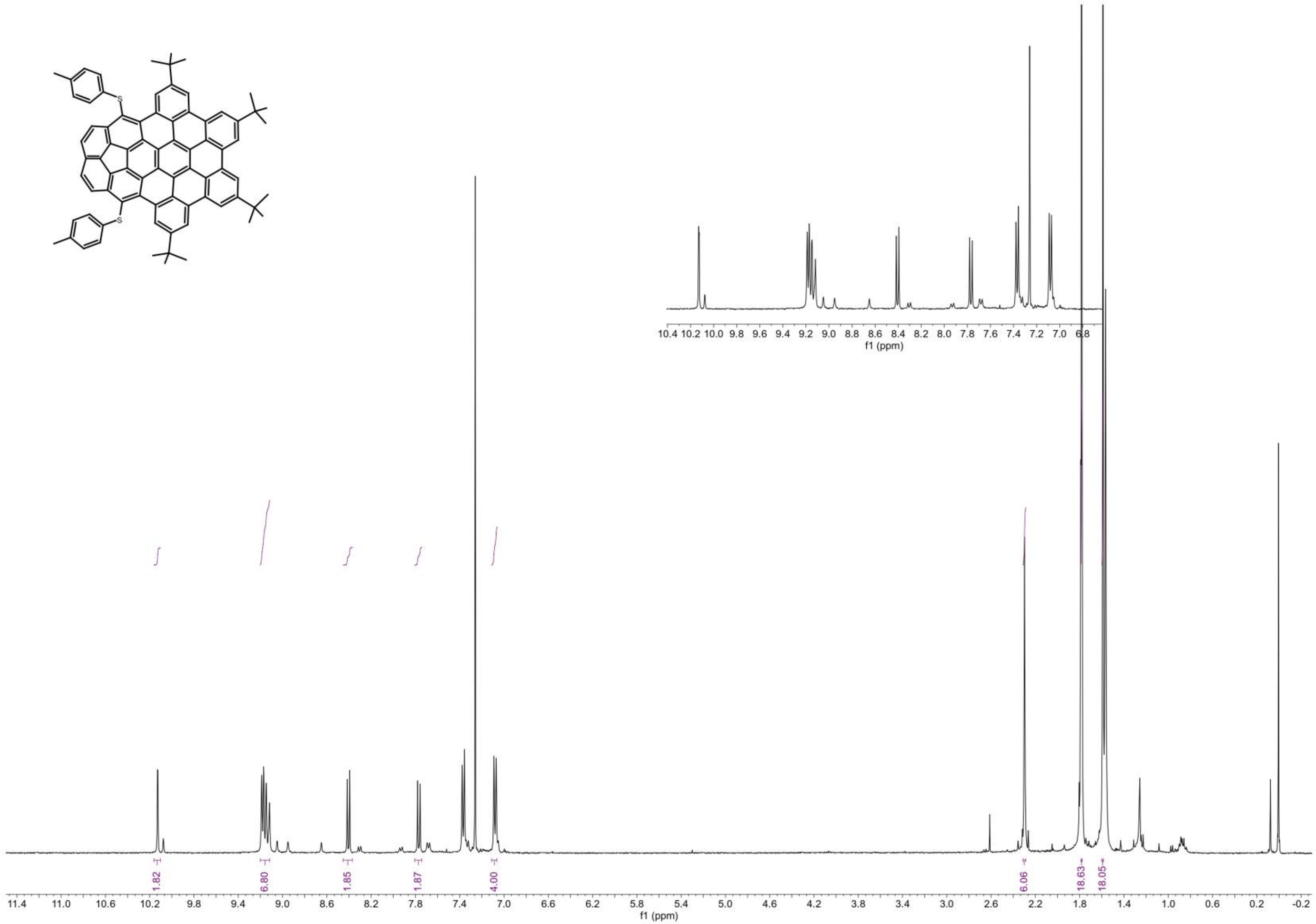

Supplementary Figure 74. NMR (400 MHz) of compound **21** in deuterated chloroform at room temperature.

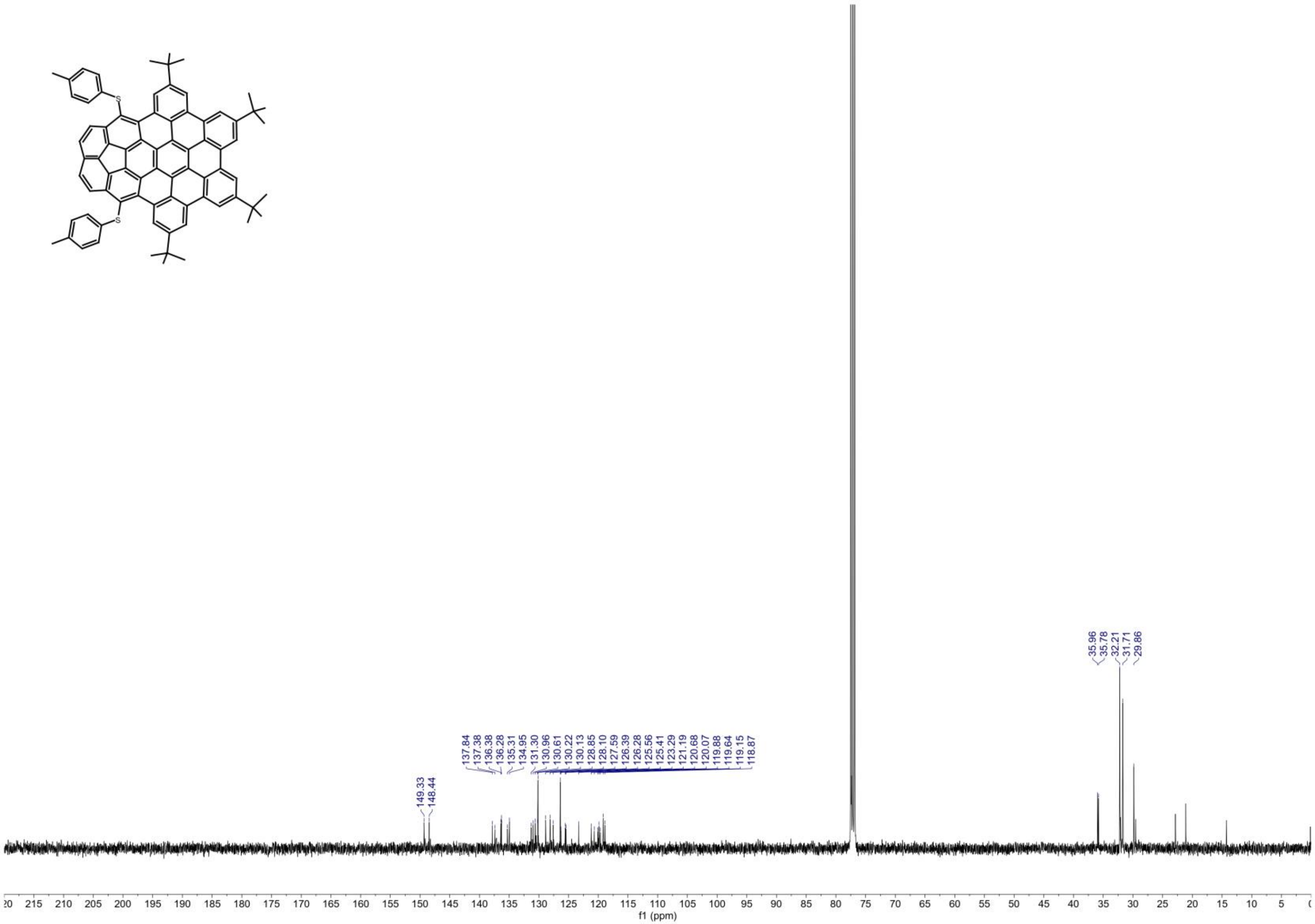

Supplementary Figure 75. NMR (101 MHz) of compound **21** in deuterated chloroform at room temperature.

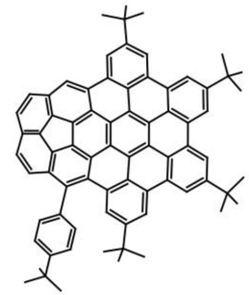

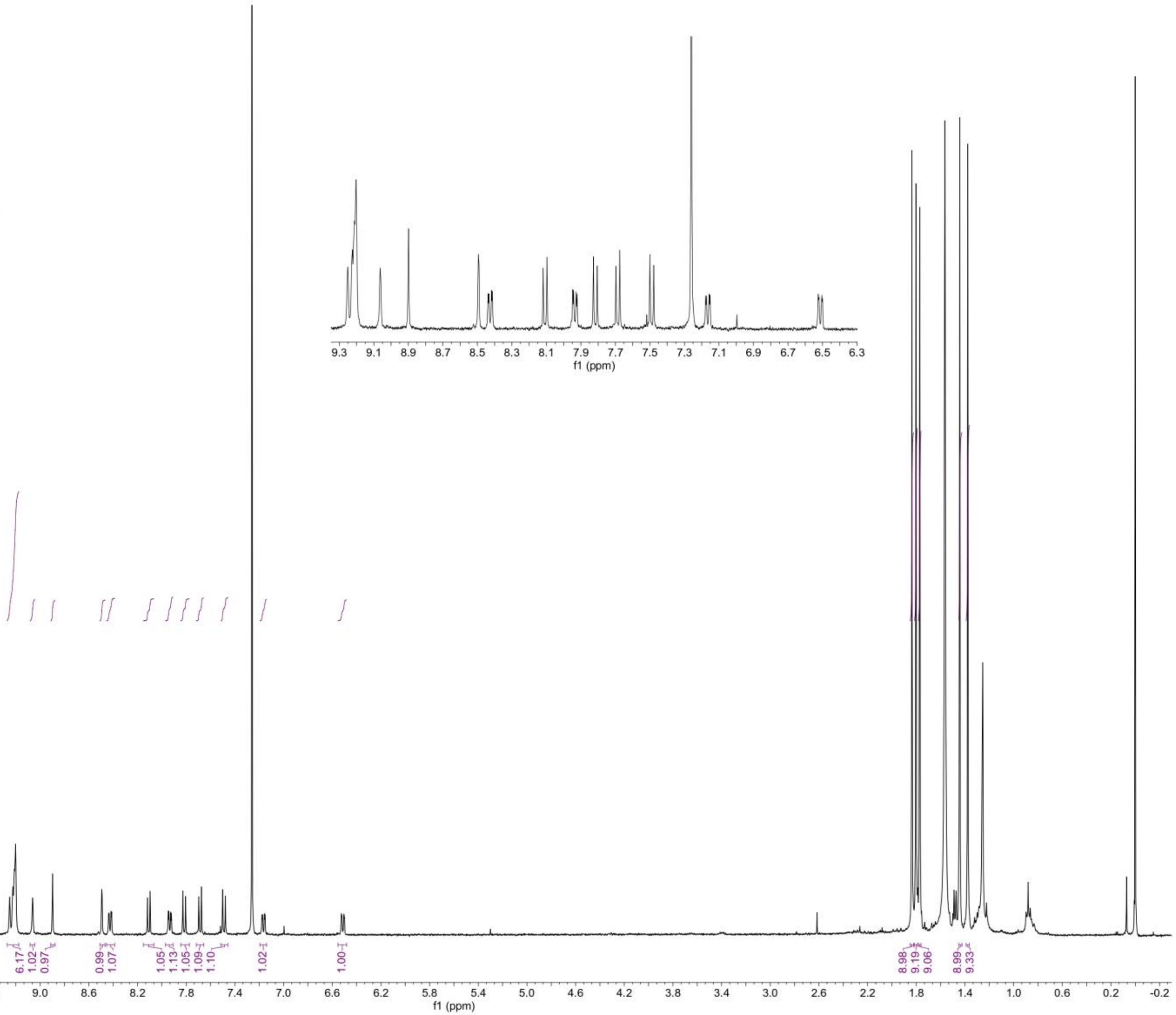

Supplementary Figure 76. NMR (400 MHz) of compound **22** in deuterated chloroform at room temperature.

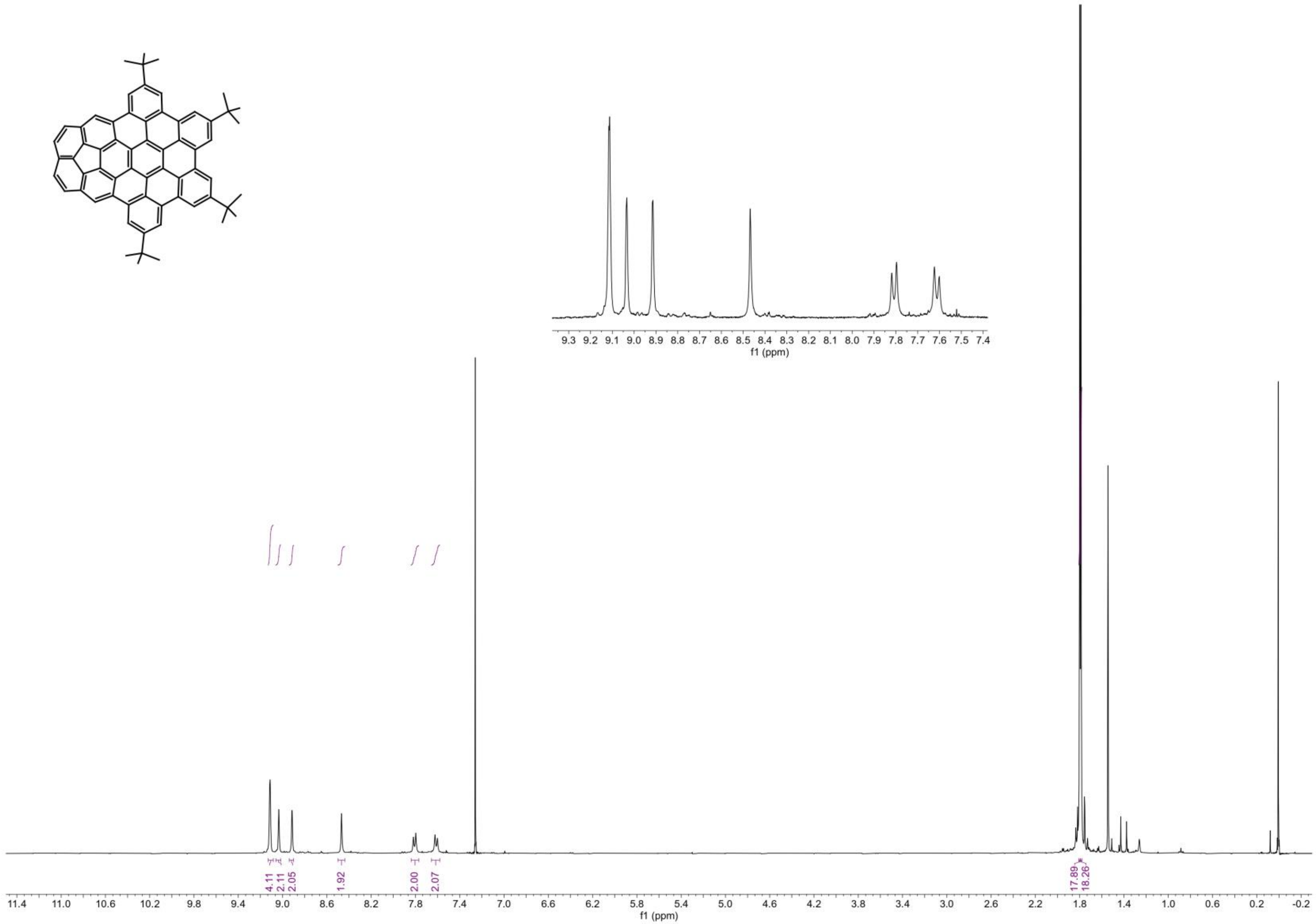

Supplementary Figure 77. NMR (400 MHz) of compound **23** in deuterated chloroform at room temperature.

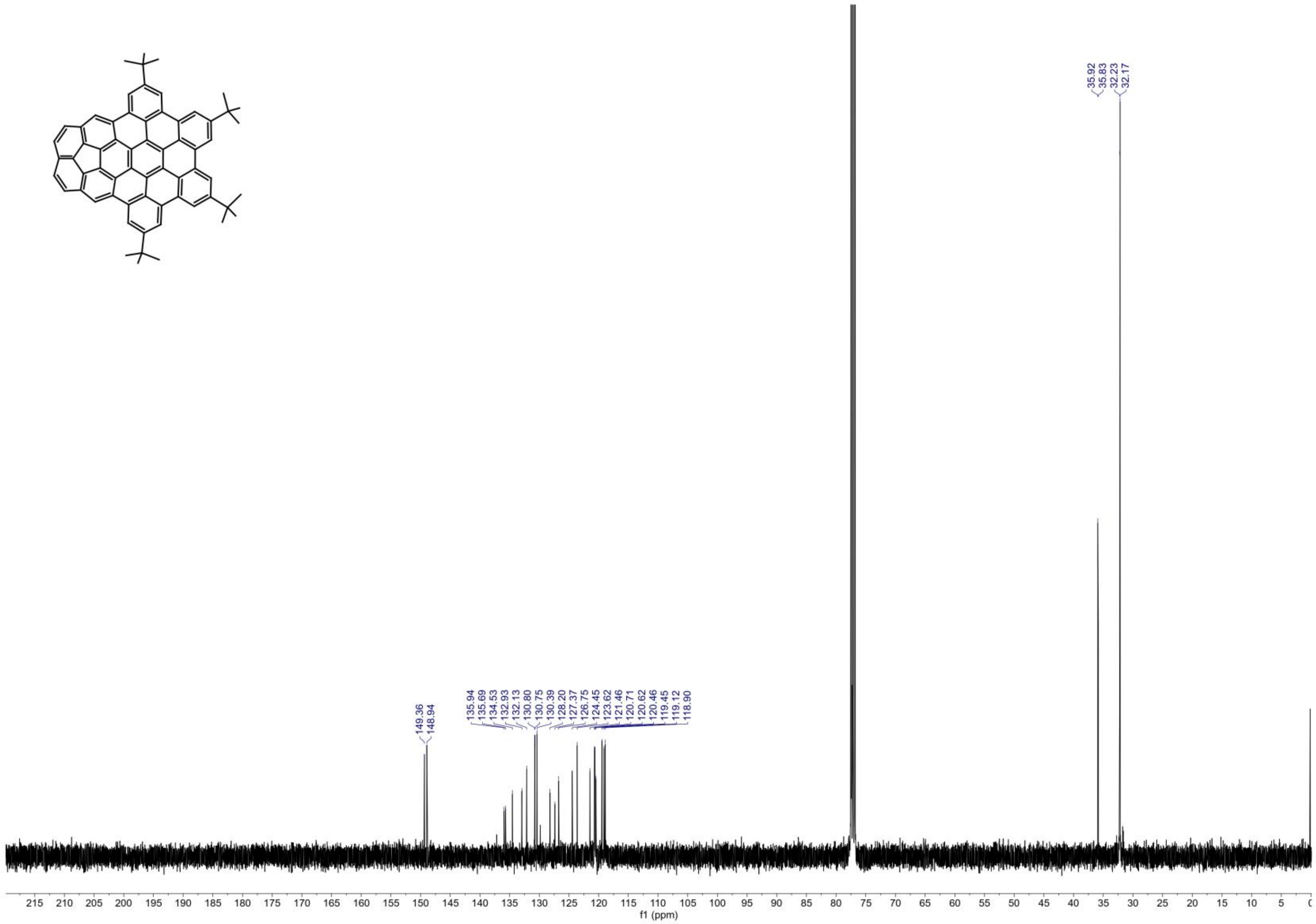

Supplementary Figure 78. NMR (100 MHz) of compound **23** in deuterated chloroform at room temperature.

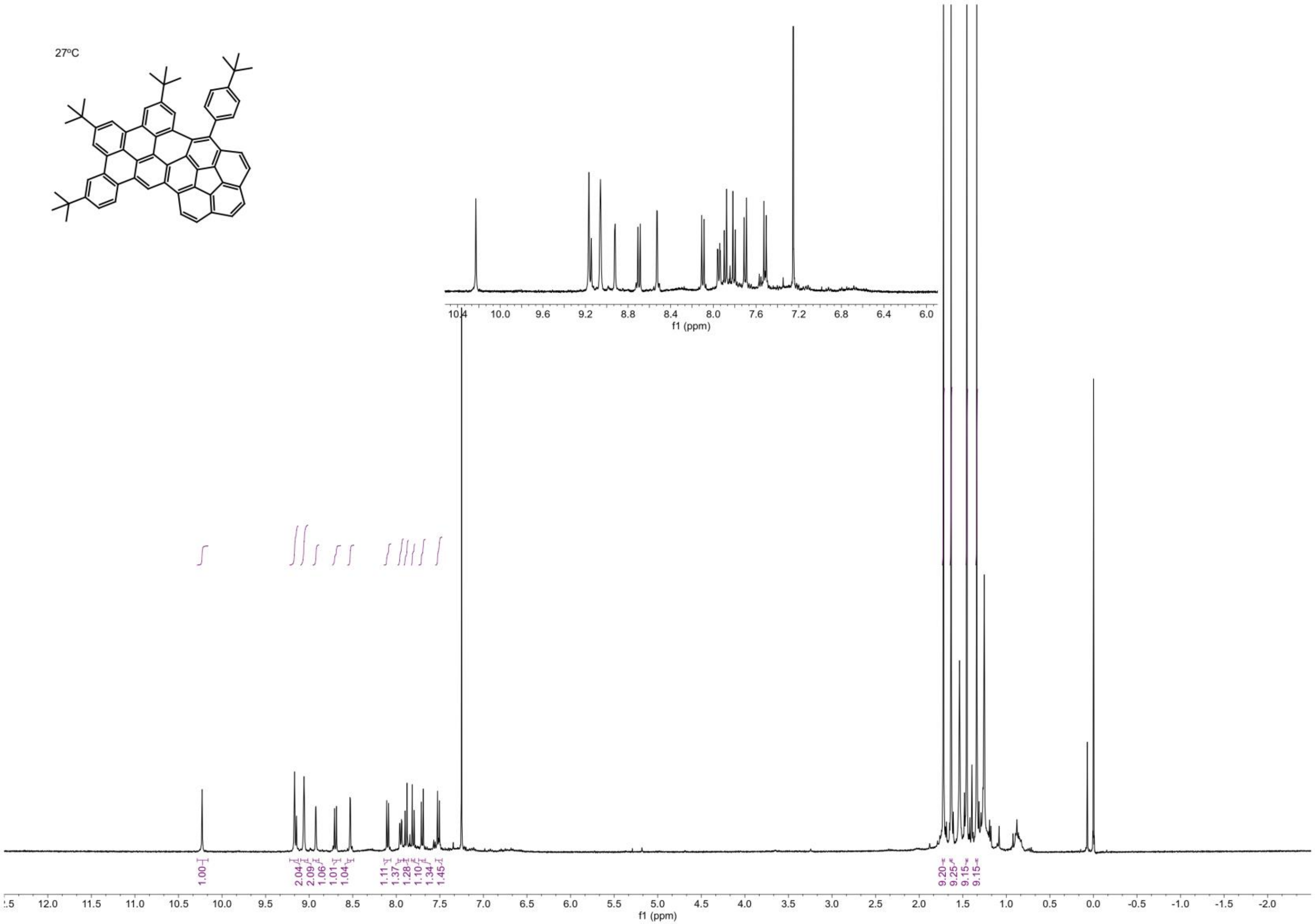

Supplementary Figure 79. NMR (400 MHz) of compound **24** in deuterated chloroform at 27 ºC.

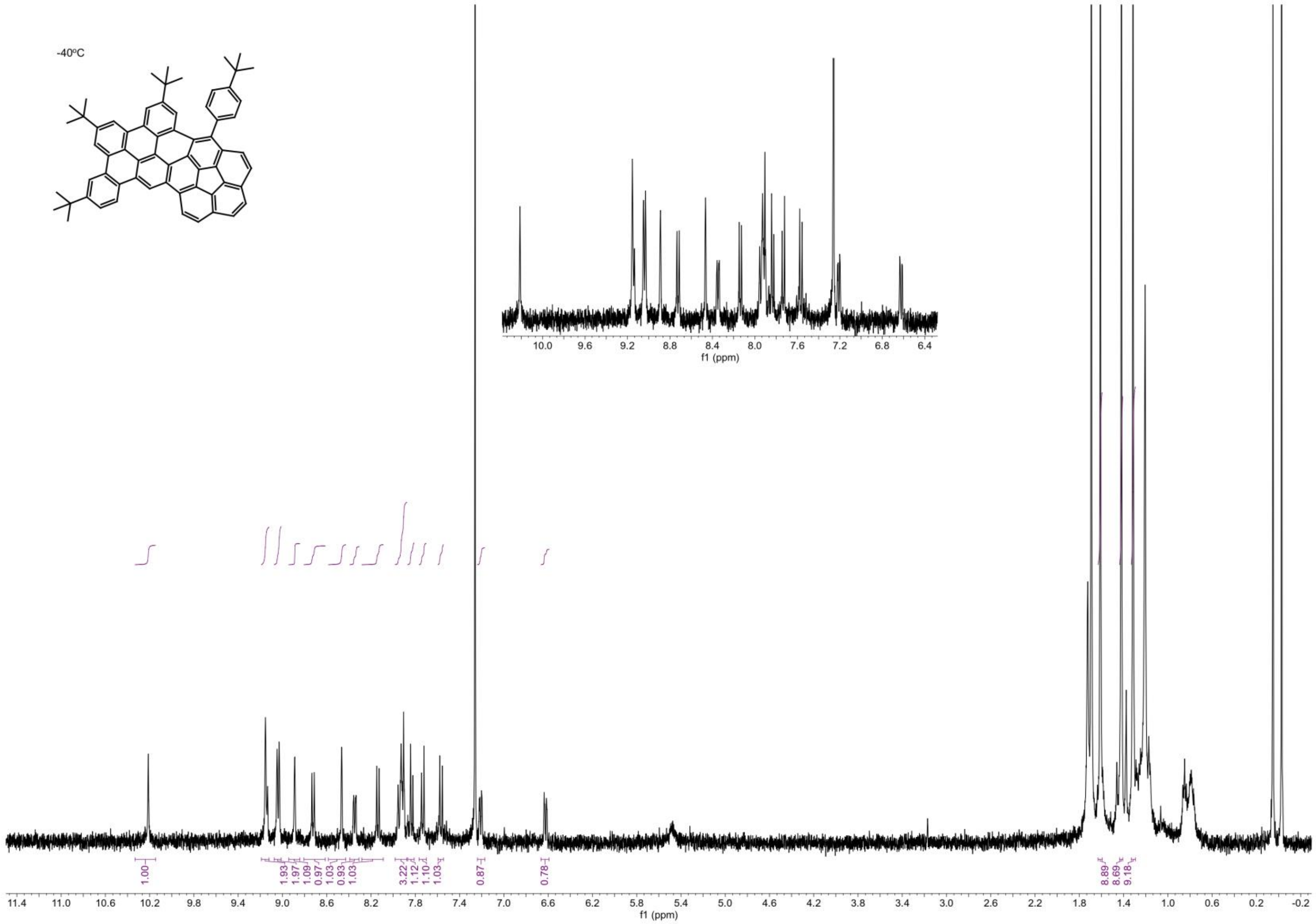

Supplementary Figure 80. NMR (400 MHz) of compound **24** in deuterated chloroform at -40 ºC.

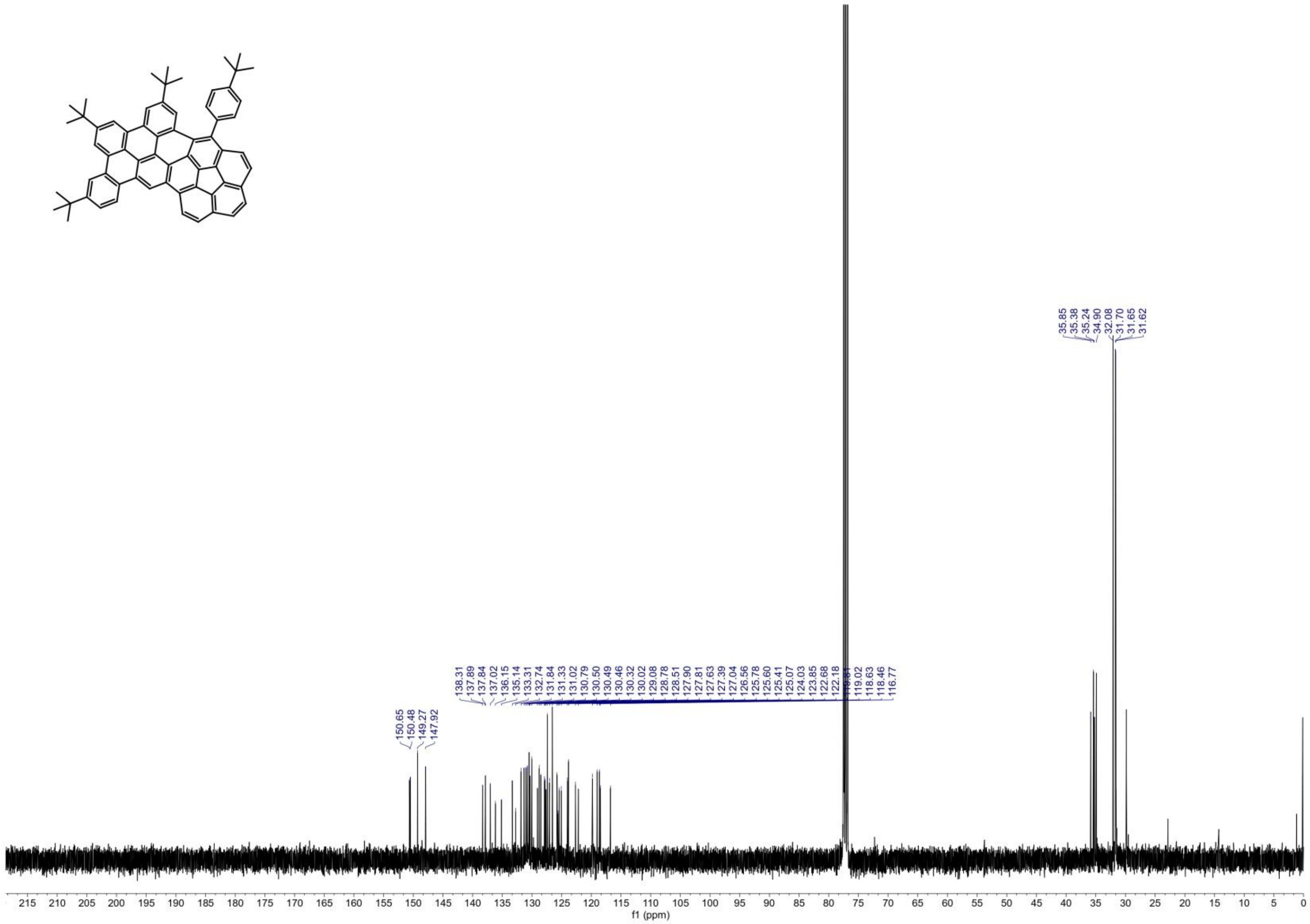

Supplementary Figure 81. NMR (100 MHz) of compound **24** in deuterated chloroform at room temperature.

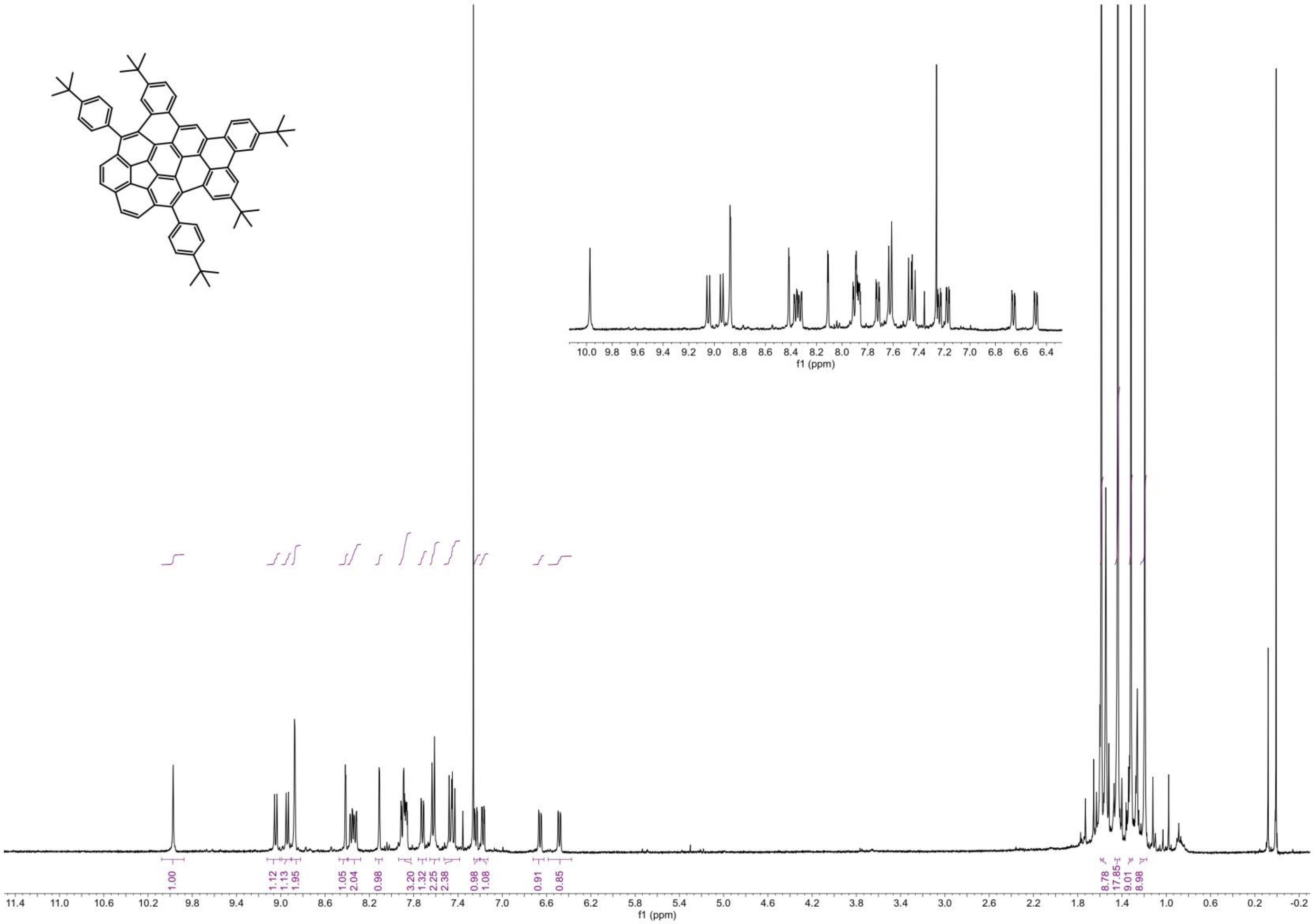

Supplementary Figure 82. NMR (400 MHz) of compound **25** in deuterated chloroform at room temperature.

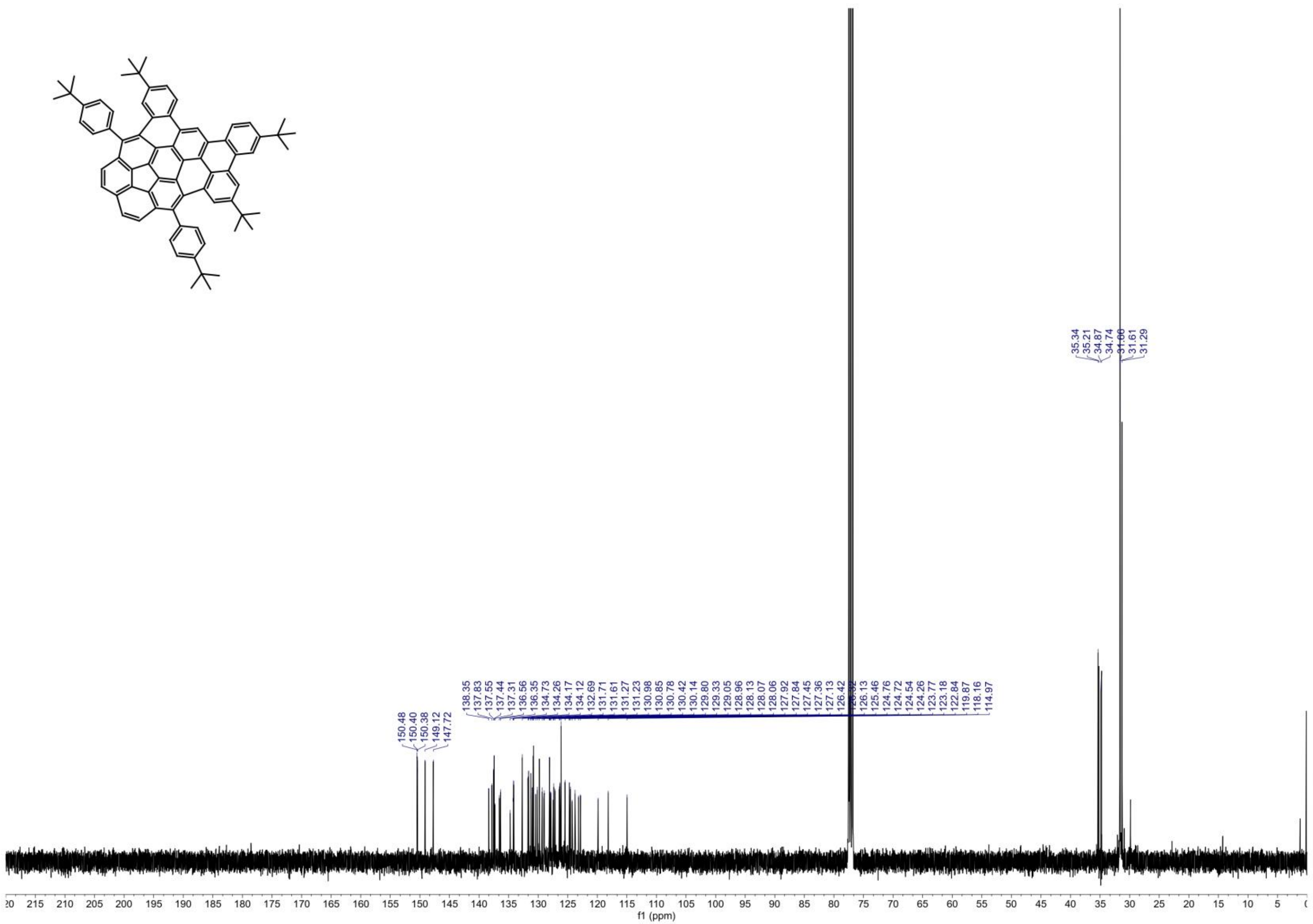

Supplementary Figure 83. NMR (100 MHz) of compound **25** in deuterated chloroform at room temperature.

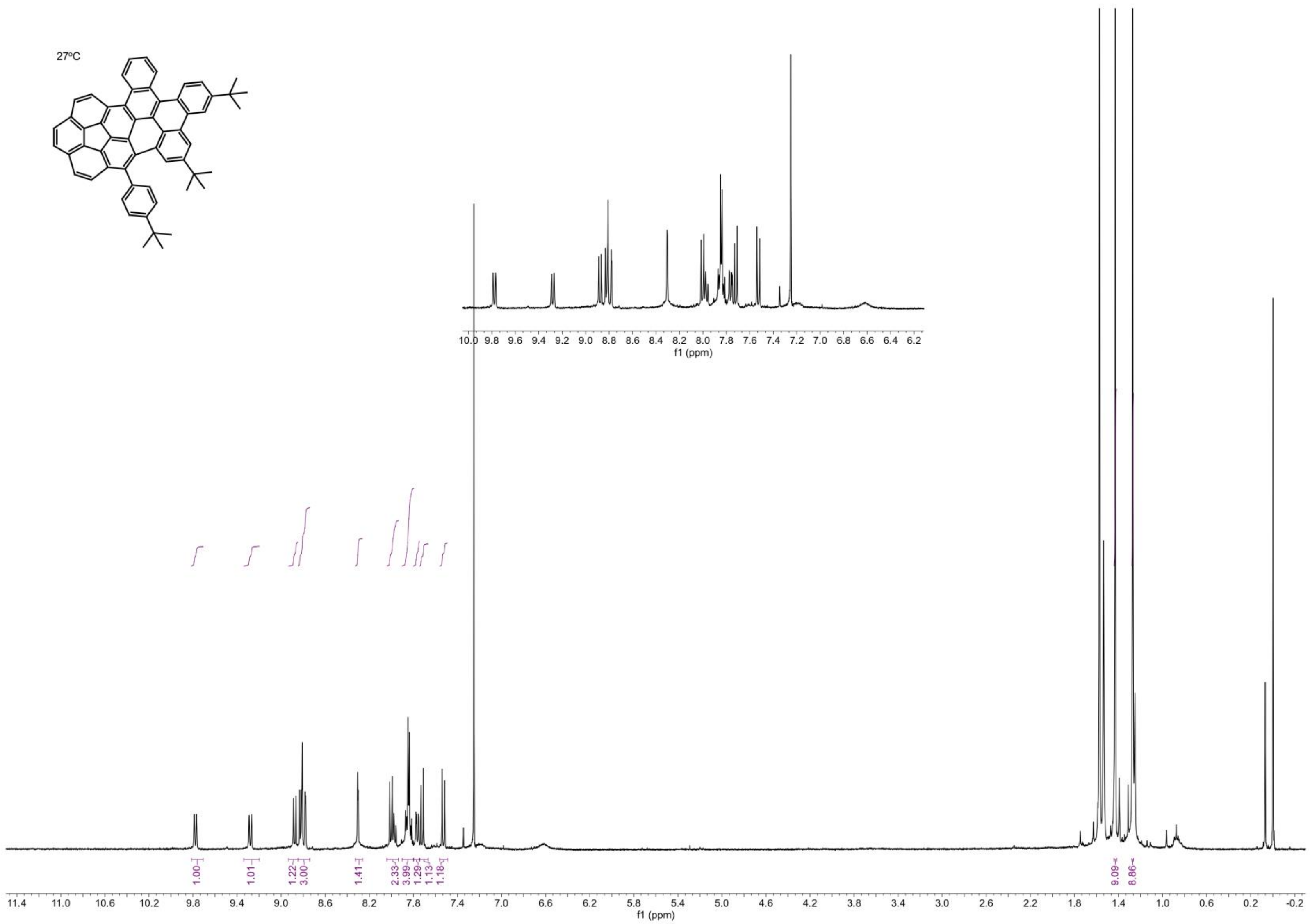

Supplementary Figure 84. NMR (400 MHz) of compound **26** in deuterated chloroform at 27 ºC.

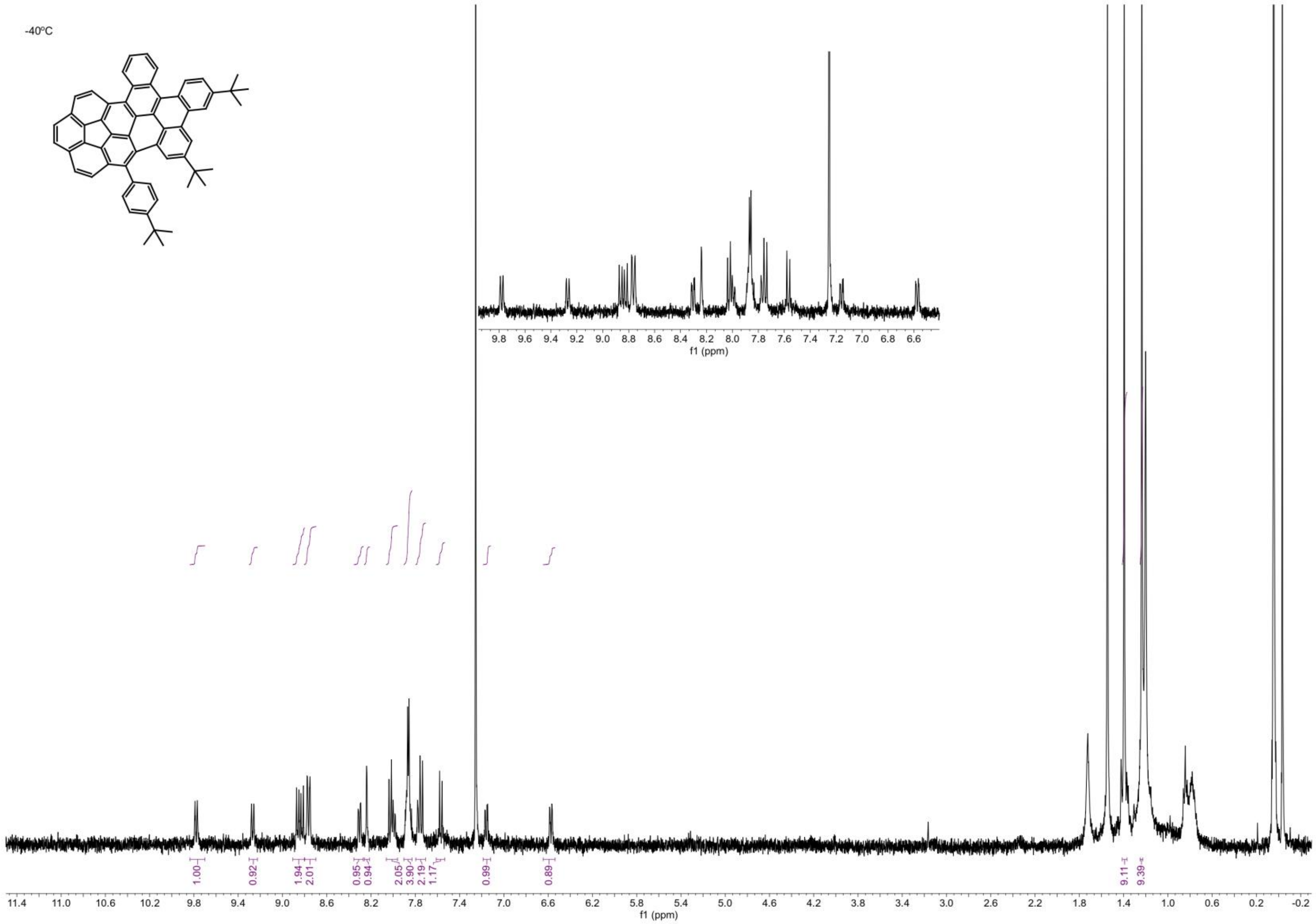

Supplementary Figure 85. NMR (400 MHz) of compound **26** in deuterated chloroform at -40 ºC.

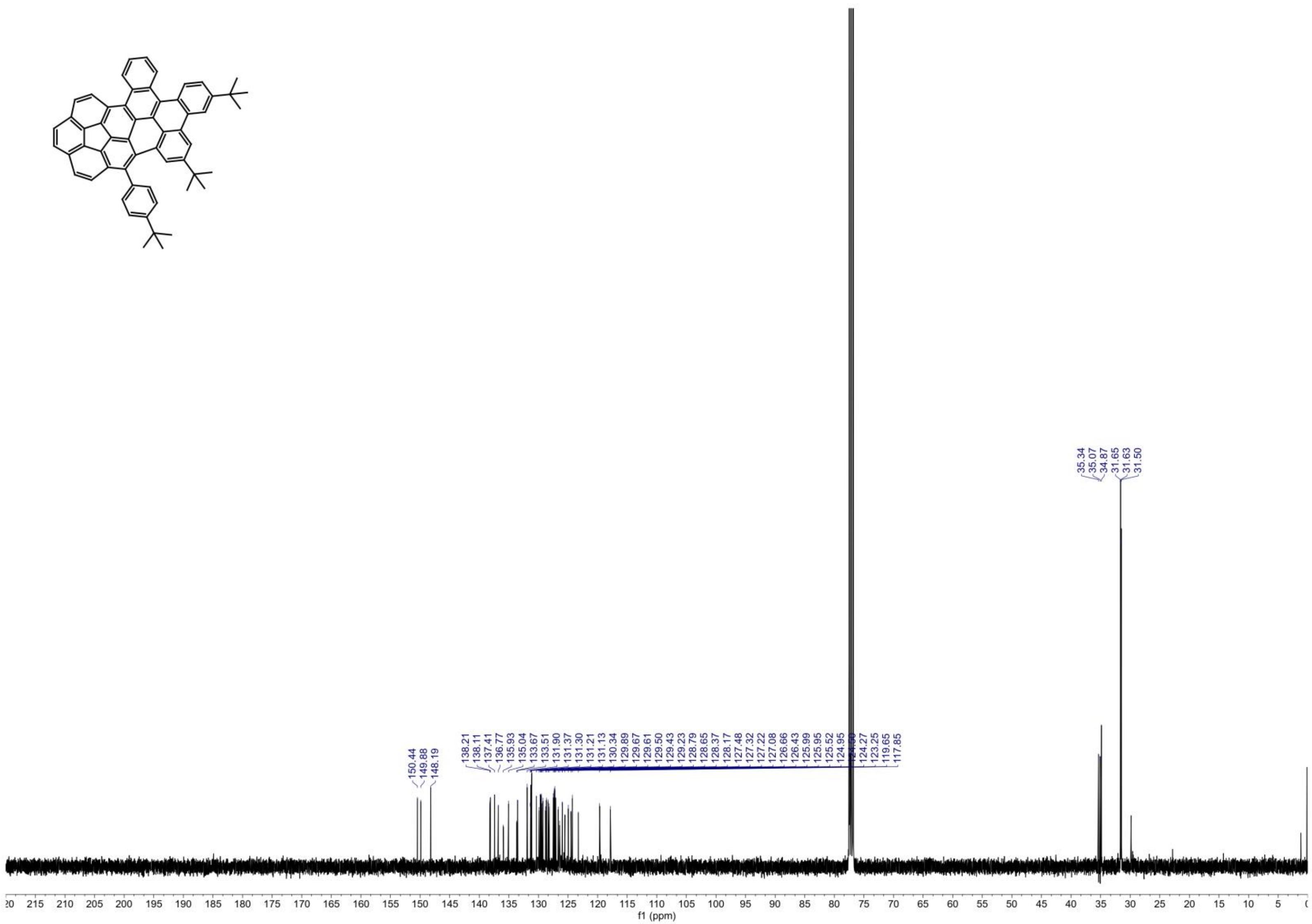

Supplementary Figure 86. NMR (100 MHz) of compound **26** in deuterated chloroform at room temperature.

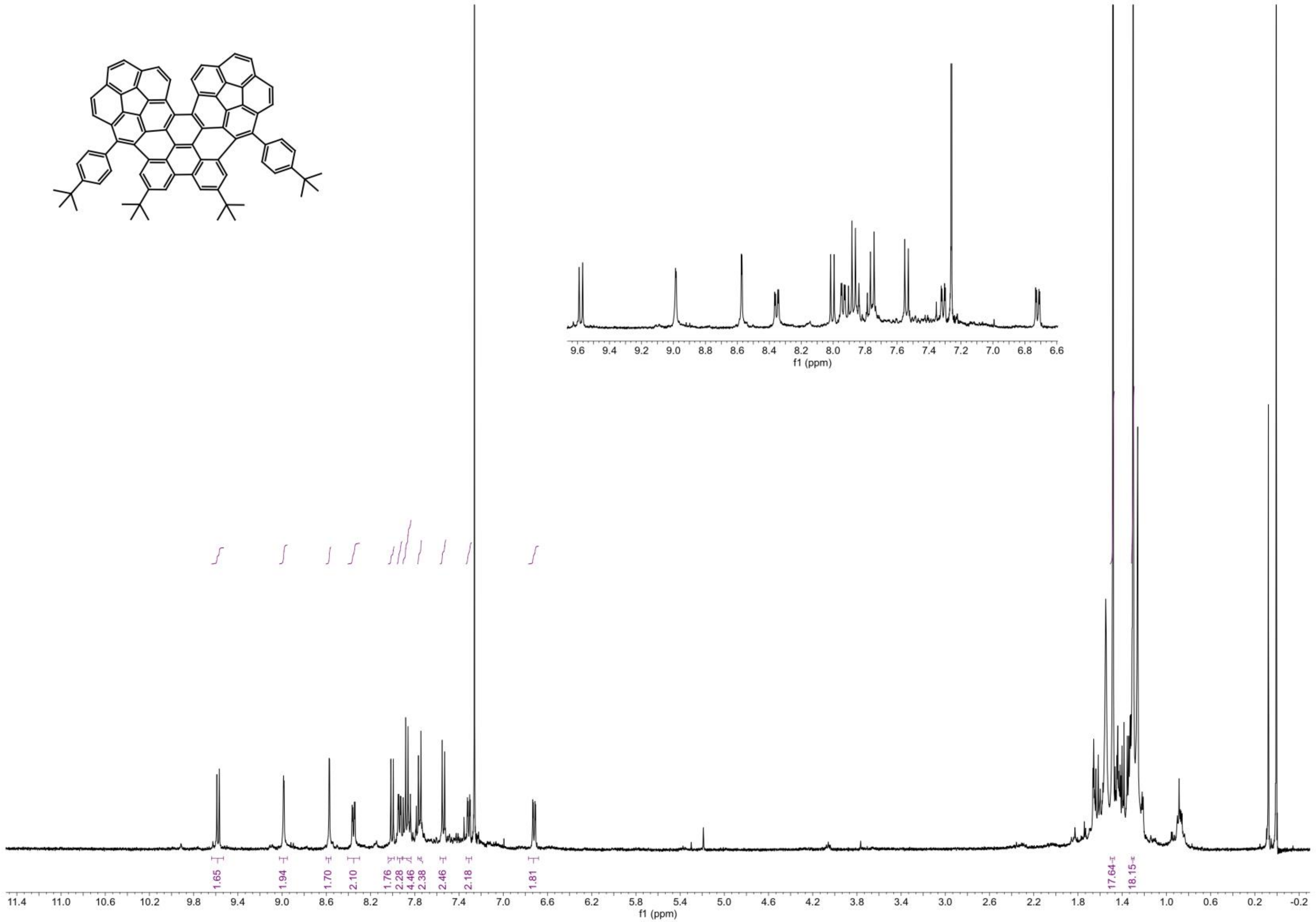

Supplementary Figure 87. NMR (400 MHz) of compound **27** in deuterated chloroform at room temperature.

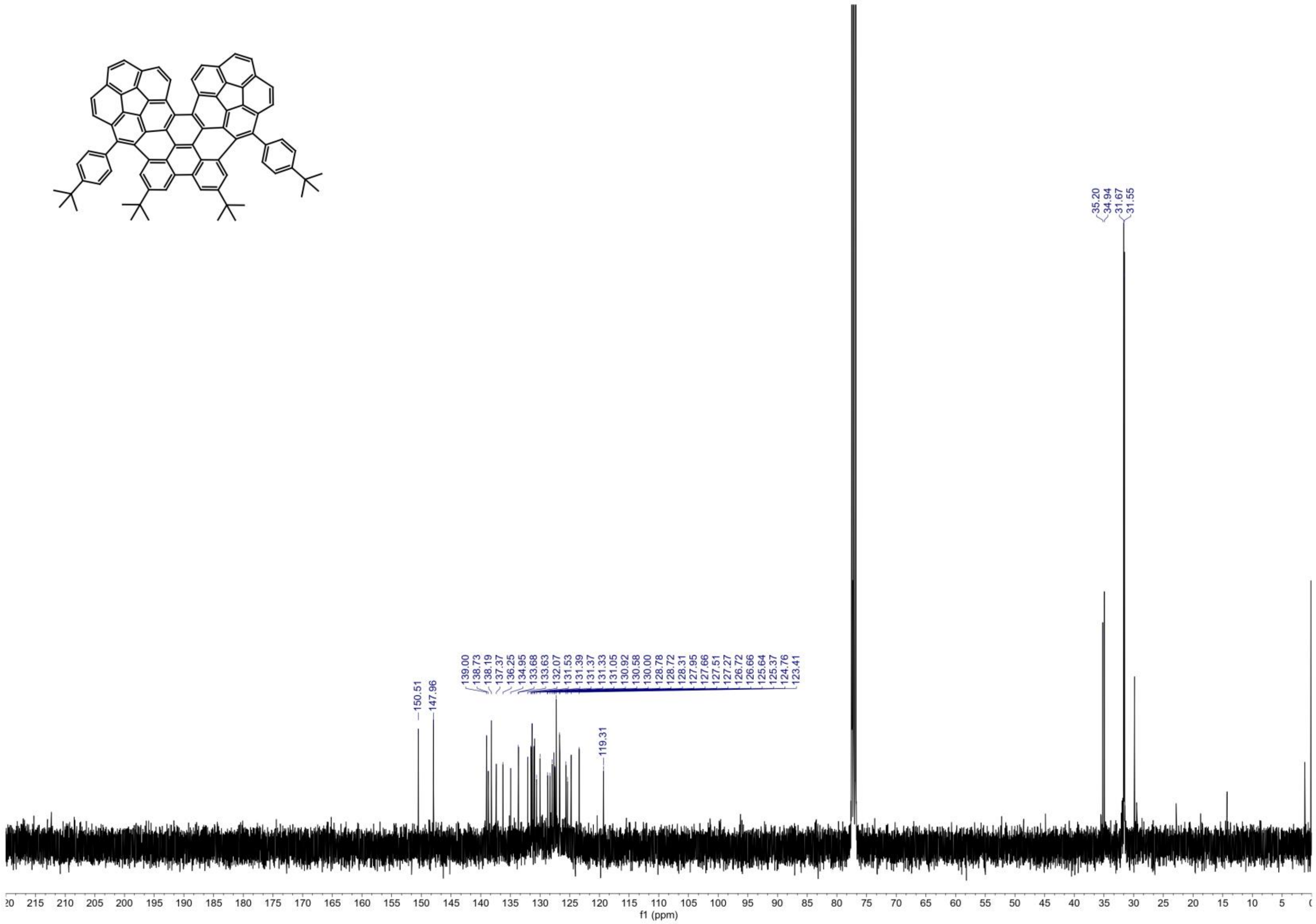

Supplementary Figure 88. NMR (100 MHz) of compound **27** in deuterated chloroform at room temperature.

Computational Details

All the calculations reported in this paper were obtained with the GAUSSIAN 09 suite of programs[2]. Electron correlation was partially taken into account using the B3LYP[3] functional in conjuction with the D3 dispersion correction suggested by Grimme et al.[4] and the double-ζ quality plus polarization functions def2-SVP[5] basis set for all atoms. All species were characterized by frequency calculations[6], and have positive definite Hessian matrices. This level is denoted B3LYP-D3/def2-SVP, which was proven to provide good results for strongly related systems[7]. Calculations of the absorption spectrum were accomplished using time-dependent density functional theory (TD-DFT)[8] at the B3LYP-D3/def2-SVP level using the optimized geometries. The assignment of the excitation energies to the experimental bands was performed on the basis of the energy values and oscillator strengths. The B3LYP Hamiltonian was chosen because it was proven to provide reasonable UV-vis spectra for a variety of chromophores[9].

The aromaticity of the considered species has been assessed by the computation of the NICS[10] values computed using the gauge invariant atomic orbital (GIAO) method[11] at the same DFT level. Ring currents were computed by means of the the Anisotropy of the Induced Current Density (AICD) method[12].

The condensed Fukui functions (f_k^-) were obtained according to eq 1:

$$f_k^- = q_k(N) - q_k(N-1) \qquad (eq.\ 1)$$

where q_k are the atomic charges at the kth atomic site and $q_k(N)$ and $q_k(N-1)$ are the electron populations on atom k for the N and $N-1$ electron species[13]. NBO atomic charges were used in the calculation of f_k^- by using the NBO6.0 program[14] at the same DFT level.

Supplementary References

1. Kissinger, P., Heineman, W. R. Laboratory Techniques in Electroanalytical Chemistry, Second Edition, Revised and Expanded; Taylor & Francis, 1996.

2. Gaussian 09, Revision D.01. Frisch, M. J., Trucks, G. W., Schlegel, H. B., Scuseria, G. E., Robb, M. A., Cheeseman, J. R., Scalmani, G., Barone, V., Petersson, G. A., Nakatsuji, H., Li, X., Caricato, M., Marenich, A. V., Bloino, J., Janesko, B. G., Gomperts, R., Mennucci, B., Hratchian, H. P., Ortiz, J. V., Izmaylov, A. F., Sonnenberg, J. L., Williams-Young, D., Ding, F., Lipparini, F., Egidi, F., Goings, J., Peng, B., Petrone, A., Henderson, T., Ranasinghe, D., Zakrzewski, V. G., Gao, J., Rega, N., Zheng, G., Liang, W., Hada, M., Ehara, M., Toyota, K., Fukuda, R., Hasegawa, J., Ishida, M., Nakajima, T., Honda, Y., Kitao, O., Nakai, H., Vreven, T., Throssell, K., Montgomery, J. A. Jr., Peralta, J. E., Ogliaro, F., Bearpark, M. J., Heyd, J. J., Brothers, E. N., Kudin, K. N., Staroverov, V. N., Keith, T. A., Kobayashi, R., Normand, J., Raghavachari, K., Rendell, A. P., Burant, J. C., Iyengar, S. S., Tomasi, J., Cossi, M., Millam, J. M., Klene, M., Adamo, C., Cammi, R., Ochterski, J. W., Martin, R. L., Morokuma, K., Farkas, O., Foresman, J. B. & Fox, D. J. Gaussian, Inc., Wallingford CT, 2016.

3. (a) Becke, A. D. Density-functional thermochemistry. III. The role of exact exchange. *J. Chem. Phys*. **98**, 5648-5652 (1993). (b) Lee, C., Yang, W. & Parr, R. G. Development of the Colle-Salvetti correlation-energy formula into a functional of the electron density. *Phys. Rev. B*. **37**, 785-789 (1998). (c) Vosko, S. H., Wilk, L. & Nusair, M. Accurate spin-dependent electron liquid correlation energies for local spin density calculations: a critical analysis. *Can. J. Phys*. **58**, 1200-1211 (1980).

4. Grimme, S., Antony, J., Ehrlich, S. & Krieg, H. A consistent and accurate ab initio parametrization of density functional dispersion correction (DFT-D) for the 94 elements H-Pu. *J. Chem. Phys*. **132**, 154104-154122 (2010).

5. Weigend, F. & Alhrichs, R. Balanced basis sets of split valence, triple zeta valence and quadruple zeta valence quality for H to Rn: Design and assessment of accuracy. *Phys. Chem. Chem. Phys*. **7**, 3297-3305 (2005).

6. McIver, J. W. & Komornicki, A. K. Structure of transition states in organic reactions. General theory and an application to the cyclobutene-butadiene isomerization using a semiempirical molecular orbital method. *J. Am. Chem. Soc*. **94**, 2625-2633 (1972).

7. See, for instance: (a) Zhou, Z., Fernández-García, J. M., Zhu, Y., Evans, P. J., Rodríguez, R., Crassous, J., Wei, Z., Fernández, I., Petrukina, M. A. & Martín, N. Site-Specific Reduction-Induced Hydrogenation of a Helical Bilayer Nanographene with K and Rb Metals: Electron Multiaddition and Selective Rb+ Complexation. *Angew. Chem. Int. Ed.* **61**, e202115747 (2022). (b) Zhou, Z., Zhu, Y., Fernández-García, J. M., Wei, Z., Fernández, I., Petrukhina, M. A. & Martín, N. Stepwise reduction of a corannulene-based helical molecular nanographene with Na metal. *Chem. Commun.* **58**, 5574-5577 (2022).

8. (a) Casida, M. E. Recent Developments and Applications of Modern Density Functional Theory; Elsevier: Amsterdam, 1996; Vol. 4. (b) Casida, M. E. & Chong, D. P. Recent Advances in Density Functional Methods; World Scientific: Singapore, 1995; Vol. 1, p 155.

9. For a review, see: Dreuw, A. & Head-Gordon, M. Single-Reference ab Initio Methods for the Calculation of Excited States of Large Molecules. *Chem. Rev.* **105**, 4009-4037 (2005).

10. Chen, Z., Wannere, C. S., Corminboeuf, C., Puchta, R. & Schleyer, P. v. R. Nucleus-Independent Chemical Shifts (NICS) as an Aromaticity Criterion. *Chem. Rev.* **105**, 3842-3888 (2005).

11. Wolinski, K., Hilton, J. F. & Pulay, P. Efficient implementation of the gauge-independent atomic orbital method for NMR chemical shift calculations. *J. Am. Chem. Soc.* **112**, 8251-8260 (1990).

12. (a) Herges, R. & Geuenich, D. Delocalization of Electrons in Molecules. *J. Phys. Chem. A* **105**, 3214-3220 (2001). (b) Geuenich, D., Hess, K., Köhler, F. & Herges, R. Anisotropy of the Induced Current Density (ACID), a General Method To Quantify and Visualize Electronic Delocalization. *Chem. Rev.* **105**, 3758-3772 (2005).

13. Parr, R. G., & Yang, W. Density-Functional Theory of Atoms and Molecules; Oxford University Press: New York, USA, 1989.

14. Glendening, E. D., Landis, R. & Weinhold, F. NBO 6.0: Natural bond orbital analysis program. *J. Comput. Chem.* **34**, 1429-1437 (2013).
