# Supplementary material for: Synthesis of precisely functionalizable curved nanographenes via graphitization-induced regioselective chlorination in a mechanochemical Scholl Reaction
Source: Nat Commun. 2023 Feb 13;14:803. doi: 10.1038/s41467-023-36470-8 (PMC9925806; doi:10.1038/s41467-023-36470-8)
Supplement: Supplementary file 3 — Description of Additional Supplementary Files [file 41467_2023_36470_MOESM3_ESM.pdf]

Description of Additional Supplementary Files

Supplementary Data 1: contains the cartesian coordinates of the structures.
