# Supplementary material for: Synthesis of precisely functionalizable curved nanographenes via graphitization-induced regioselective chlorination in a mechanochemical Scholl Reaction
Source: Nat Commun. 2023 Feb 13;14:803. doi: 10.1038/s41467-023-36470-8 (PMC9925806; doi:10.1038/s41467-023-36470-8)
Supplement: Supplementary file 4 — Supplementary Data 1 [file 41467_2023_36470_MOESM4_ESM.pdf]

Supplementary Data

Synthesis of Precisely Functionalizable Curved Nanographenes via Graphitization-Induced Regioselective Chlorination in a Mechanochemical Scholl Reaction

Jovana Stanojkovic, Ronny William, Zhongbo Zhang, Israel Fernández, Jingsong Zhou, Richard D. Webster, and Mihaiela C. Stuparu*

*Email:mstuparu@ntu.edu.sg

Cartesian coordinates (in Å) and total energies (in a.u., ZPVE included) of all the stationary points discussed in the text. All calculations have been performed at the B3LYP-D3/def2-SVP level.

4': E= -1496.947889

C	-4.053299000	-1.123251000	-0.657843000
C	-4.636976000	0.143438000	-0.399934000
C	-3.611384000	1.120846000	-0.480044000
C	-2.391189000	0.460888000	-0.790025000
C	-2.669470000	-0.927386000	-0.902234000
C	-1.719754000	-1.914825000	-0.651568000
C	-2.263276000	-3.222091000	-0.331984000
C	-3.620296000	-3.404294000	-0.096218000
C	-4.570509000	-2.307606000	-0.151287000
C	-5.869361000	-2.174847000	0.474095000
H	-6.376993000	-3.069570000	0.846448000
C	-6.445040000	-0.931536000	0.724238000
H	-7.384687000	-0.893252000	1.283168000
C	-5.776686000	0.305093000	0.376112000
C	-5.944860000	1.637248000	0.921230000
H	-6.847447000	1.870926000	1.493736000
C	-4.942172000	2.598564000	0.839663000
H	-5.090906000	3.554613000	1.350123000

C	-3.668325000	2.325688000	0.205918000
C	-2.383873000	2.968797000	0.390848000
C	-1.185553000	2.337391000	0.079222000
C	-1.139231000	0.986186000	-0.455836000
C	-0.093823000	-0.014135000	-0.453834000
C	1.380544000	0.136286000	-0.217774000
C	2.050258000	1.448276000	-0.136539000
C	2.890287000	1.767407000	0.946735000
C	3.528805000	3.002992000	1.011301000
H	4.171354000	3.230067000	1.867124000
C	3.366295000	3.962968000	-0.003686000
C	2.532594000	3.639303000	-1.084167000
H	2.389253000	4.364477000	-1.890070000
C	1.878833000	2.406875000	-1.149208000
C	4.405727000	-0.710822000	-0.575694000
C	3.381439000	-1.457464000	0.046109000
C	3.758227000	-2.588468000	0.796150000
C	5.102716000	-2.937541000	0.938165000
H	5.364901000	-3.817232000	1.533006000
C	6.120502000	-2.187560000	0.333069000
C	5.741902000	-1.069698000	-0.432197000
H	6.512594000	-0.473944000	-0.930698000
C	1.966713000	-1.094543000	-0.100522000
C	0.872521000	-2.132607000	-0.253834000
C	-0.393897000	-1.386899000	-0.524492000
H	-3.963869000	-4.390754000	0.229282000
H	-2.341547000	3.948455000	0.875933000
H	-1.571385000	-4.053945000	-0.188450000
H	2.988487000	-3.199670000	1.266938000
C	7.575269000	-2.556744000	0.482048000
H	7.705070000	-3.474066000	1.075231000
H	8.047411000	-2.719829000	-0.501364000
H	8.141870000	-1.750675000	0.978777000
C	4.065232000	5.296676000	0.084574000

H	3.905616000	5.902040000	-0.819895000
H	3.700982000	5.878867000	0.948122000
H	5.151886000	5.168499000	0.220565000
H	-0.255941000	2.847283000	0.331706000
H	1.229527000	2.181337000	-1.998412000
H	4.144550000	0.153480000	-1.188089000
H	3.037997000	1.031638000	1.739875000
O	0.988493000	-3.338048000	-0.165799000

7’: E= -1995.451054

C	-4.381985000	-1.156281000	-0.677700000
C	-5.173579000	0.000016000	-0.413219000
C	-4.381979000	1.156309000	-0.677689000
C	-3.105232000	0.714563000	-1.086756000
C	-3.105236000	-0.714539000	-1.086762000
C	-1.964842000	-1.444359000	-0.818306000
C	-2.126447000	-2.796209000	-0.349851000
C	-3.409139000	-3.233495000	0.042793000
C	-4.559210000	-2.370460000	-0.007905000
C	-5.775152000	-2.435932000	0.783015000
H	-6.037613000	-3.373864000	1.281665000
C	-6.551012000	-1.310801000	1.034790000
H	-7.397163000	-1.403591000	1.722211000
C	-6.198699000	0.000015000	0.522588000
C	-6.551005000	1.310826000	1.034801000
H	-7.397153000	1.403616000	1.722225000
C	-5.775138000	2.435956000	0.783035000
H	-6.037593000	3.373884000	1.281697000
C	-4.559196000	2.370483000	-0.007883000
C	-3.409118000	3.233510000	0.042826000
C	-2.126431000	2.796223000	-0.349831000
C	-1.964834000	1.444375000	-0.818296000
C	-0.727058000	0.715581000	-0.707438000

C	0.452068000	1.427370000	-0.391988000
C	0.386110000	2.864335000	-0.212364000
C	-0.875225000	3.558168000	-0.182564000
C	-0.879238000	4.942128000	0.009870000
H	-1.830075000	5.480185000	0.002196000
C	0.305030000	5.670811000	0.193221000
C	1.517652000	4.983026000	0.190072000
H	2.428987000	5.557116000	0.356948000
C	1.592389000	3.589399000	-0.006988000
C	2.875526000	2.869081000	0.021669000
C	2.894192000	1.442049000	-0.035809000
C	4.131069000	0.736312000	0.070732000
C	5.315552000	1.477043000	0.184083000
H	6.275234000	0.964643000	0.258937000
C	5.320316000	2.875964000	0.204108000
C	4.100432000	3.549671000	0.129924000
H	4.116712000	4.639238000	0.152151000
C	4.131067000	-0.736333000	0.070749000
C	2.894180000	-1.442066000	-0.035793000
C	1.665618000	-0.714887000	-0.222867000
C	1.665621000	0.714880000	-0.222869000
C	0.452060000	-1.427371000	-0.391991000
C	-0.727062000	-0.715573000	-0.707441000
C	0.386093000	-2.864335000	-0.212372000
C	-0.875246000	-3.558162000	-0.182586000
C	-0.879269000	-4.942124000	0.009830000
H	-1.830108000	-5.480175000	0.002141000
C	0.304994000	-5.670815000	0.193181000
C	1.517620000	-4.983036000	0.190056000
H	2.428948000	-5.557134000	0.356941000
C	1.592368000	-3.589407000	-0.006990000
C	2.875504000	-2.869092000	0.021689000
C	4.100411000	-3.549690000	0.129963000
H	4.116676000	-4.639258000	0.152185000

C	5.320293000	-2.875992000	0.204167000
C	5.315539000	-1.477067000	0.184119000
H	6.275231000	-0.964683000	0.258965000
H	-3.508847000	-4.205326000	0.532421000
H	-3.508822000	4.205339000	0.532460000
C	0.248543000	-7.166608000	0.384980000
H	-0.407999000	-7.435504000	1.229043000
H	-0.160350000	-7.664514000	-0.510537000
H	1.243634000	-7.592566000	0.581063000
C	6.626727000	-3.625985000	0.300478000
H	7.219667000	-3.284903000	1.165118000
H	6.470000000	-4.709854000	0.404138000
H	7.244733000	-3.460624000	-0.598361000
C	6.626770000	3.625950000	0.300216000
H	7.244128000	3.461490000	-0.599241000
H	6.470055000	4.709711000	0.405007000
H	7.220366000	3.284070000	1.164083000
C	0.248581000	7.166597000	0.385075000
H	-0.160516000	7.664522000	-0.510337000
H	-0.407790000	7.435447000	1.229287000
H	1.243701000	7.592575000	0.580965000

18’: E= -2146.497866

C	3.407759000	-0.902602000	-0.901373000
C	4.810460000	-1.015774000	-0.806133000
C	5.109727000	-2.334617000	-0.368296000
C	3.881687000	-3.029298000	-0.191259000
C	2.819329000	-2.151397000	-0.531770000
C	1.540189000	-2.257653000	-0.010615000
C	1.342935000	-3.434621000	0.811494000
H	0.369244000	-3.612526000	1.268977000
C	2.382207000	-4.299122000	1.148293000
H	2.170427000	-5.112823000	1.848136000

C	3.739567000	-4.071838000	0.717710000
C	4.979944000	-4.546864000	1.294736000
H	4.959875000	-5.407237000	1.970149000
C	6.180121000	-3.867172000	1.116777000
H	7.065033000	-4.216555000	1.657024000
C	6.259336000	-2.644487000	0.345443000
C	7.231300000	-1.569084000	0.419784000
H	8.195153000	-1.754789000	0.903060000
C	6.932455000	-0.276266000	0.010187000
H	7.669920000	0.512586000	0.186063000
C	5.629235000	0.078685000	-0.523372000
C	4.976357000	1.355895000	-0.534874000
H	5.578449000	2.247782000	-0.349533000
C	3.573141000	1.480833000	-0.612705000
C	2.759327000	0.293709000	-0.689182000
C	1.359011000	0.231418000	-0.344266000
C	0.703281000	-1.038116000	-0.116830000
C	-0.703365000	-1.038058000	0.116024000
C	-1.358975000	0.231502000	0.343730000
C	-0.686097000	1.475798000	0.165380000
C	0.686172000	1.475730000	-0.166027000
C	1.422581000	2.725024000	-0.282259000
C	0.725702000	3.960365000	-0.124905000
C	1.450241000	5.162072000	-0.209271000
H	0.938175000	6.115880000	-0.083712000
C	2.824984000	5.195394000	-0.449630000
C	3.500525000	3.981116000	-0.591225000
H	4.573003000	4.000268000	-0.791992000
C	2.840723000	2.748774000	-0.498072000
C	-1.422392000	2.725123000	0.281990000
C	-2.840475000	2.748954000	0.497986000
C	-3.500191000	3.981322000	0.591519000
H	-4.572589000	4.000394000	0.792752000
C	-2.824583000	5.195532000	0.449981000

C	-1.449878000	5.162173000	0.209258000
H	-0.937823000	6.116001000	0.083732000
C	-0.725434000	3.960451000	0.124461000
C	-2.759209000	0.293859000	0.688913000
C	-3.572937000	1.481010000	0.612684000
C	-4.976190000	1.356226000	0.534881000
H	-5.578097000	2.248221000	0.349442000
C	-5.629151000	0.079094000	0.523449000
C	-6.932462000	-0.275808000	-0.010005000
H	-7.669909000	0.513079000	-0.185805000
C	-7.231449000	-1.568610000	-0.419500000
H	-8.195400000	-1.754257000	-0.902604000
C	-6.259572000	-2.644115000	-0.345191000
C	-6.180520000	-3.866907000	-1.116359000
H	-7.065504000	-4.216339000	-1.656459000
C	-4.980435000	-4.546770000	-1.294303000
H	-4.960594000	-5.407263000	-1.969570000
C	-3.739927000	-4.071791000	-0.717511000
C	-2.382630000	-4.299279000	-1.148135000
H	-2.171002000	-5.113048000	-1.847943000
C	-1.343260000	-3.434811000	-0.811574000
H	-0.369675000	-3.612757000	-1.269246000
C	-1.540350000	-2.257632000	0.010278000
C	-2.819425000	-2.151213000	0.531560000
C	-3.881888000	-3.029068000	0.191280000
C	-5.109853000	-2.334275000	0.368380000
C	-4.810444000	-1.015438000	0.806177000
C	-3.407733000	-0.902390000	0.901246000
C	-3.548458000	6.516498000	0.543609000
H	-3.491503000	7.066915000	-0.410626000
H	-4.611798000	6.383496000	0.791559000
H	-3.099057000	7.163186000	1.315193000
C	3.548958000	6.516313000	-0.543162000
H	4.612802000	6.383144000	-0.788844000

H	3.101084000	7.162040000	-1.316460000
H	3.489955000	7.067847000	0.410284000
